# Parameter Extraction of Single, Double, and Triple‐Diode Photovoltaic Models Using the Weighted Leader Search Algorithm

**DOI:** 10.1002/gch2.202300355

**Published:** 2024-04-18

**Authors:** İpek Çetinbaş

**Affiliations:** ^1^ Department of Electrical and Electronics Engineering Faculty of Engineering and Architecture Eskişehir Osmangazi University Eskişehir 26480 Turkey

**Keywords:** double‐diode model, PV parameter extraction, single‐diode model, triple‐diode model, weighted leader search optimization

## Abstract

This study presents the parameter extraction of single, double, and triple‐diode photovoltaic (PV) models using the weighted leader search algorithm (WLS). The primary objective is to develop models that accurately reflect the characteristics of PV devices so that technical and economic benefits are maximized under all constraints. For this purpose, 24 models, 6 for two different PV cells, and 18 for six PV modules, whose experimental data are publicly available, are developed successfully. The second objective of this research is the selection of the most suitable algorithm for this problem. It is a significant challenge since the evaluation process requires using advanced statistical tools and techniques to determine the reliable selection. Therefore, seven brand‐new algorithms, including WLS, the spider wasp optimizer, the shrimp and goby association search, the reversible elementary cellular automata, the fennec fox optimization, the Kepler optimization, and the rime optimization algorithms, are tested. The WLS has yielded the smallest minimum, average, RMSE, and standard deviation among those. Its superiority is also verified by Friedman and Wilcoxon signed‐rank test based on 144 pairwise comparisons. In conclusion, it is demonstrated that the WLS is a superior algorithm in PV parameter extraction for developing accurate models.

## Graphical abstract

The photovoltaic (PV) parameter extraction problem' aim is to develop models that accurately reflect the characteristics of PV devices so that technical and economic benefits are maximized under all constraints. The PV parameter extraction is studied and seven brand‐new algorithms are used. The evaluation metrics, statistical tests, and sensitivity analysis show that the weighted leader search algorithm is a reliable alternative.

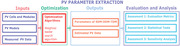

## 1 Introduction

### 1.1 Motivation

In the International Energy Agency's Renewables 2022 report, evaluations were made on the power mix consisting of renewable energy sources, nuclear, coal, oil, and natural gas. Projections for the years 2022–2027 were shared, as well. This report states that between 2017 and 2022, renewable energy sources will grow by approximately 2400 GW with an 85% increase and will be the source of electricity generation with the most significant volume in the power mix. It is thought that the share of renewable energy sources in the power mix will increase compared to other sources and will surpass the energy production from coal in electrical energy production in 2025, reach 38% in the power mix in 2027, and that electricity generated from solar and wind energy among renewable energy sources will provide 20% of global energy production in 2027. Among renewable energy sources, PV systems are expected to see significant developments in installed power and cumulative capacity. PV is expected to surpass the coal within the installed capacity in 2027 and increase by 1500 GW in cumulative capacity, surpassing natural gas and coal in 2026 and 2027, respectively.^[^ ^1^ ^]^

In the process of generating electrical energy, PV systems have a wide range of versatile uses, from individual small power applications to commercial, industrial applications, due to the advantages of being a clean and green energy source since it does not produce harmful greenhouse gases, utilizing free raw materials, silent operation, ease of installation, low operating cost, low maintenance, not creating noise pollution, safe operation and customizable structure.^[^ ^2^, ^3^, ^4^, ^5^, ^6^ ^]^ Due to these economic and environmental factors, PV power applications are a frequently preferred, promising, and modern technology being studied.^[^ ^7^, ^8^, ^9^ ^]^ However, the initial cost, that is, the investment cost, of PV systems, which offer a long‐term clean solution to energy problems by converting solar energy into electrical energy, is high. Optimal design and energy conversion efficiency are essential to maximize the benefits obtained from these systems.^[^ ^10^, ^11^ ^]^

The electrical properties and efficiency of PV cells can vary depending on the material they are made of. However, PV cells are also affected by meteorological conditions such as irradiance and temperature. This suggests that their behavior can show instant variations at any time in a day or a season. The variation of their performance due to the dependence on these factors is a common characteristic of all PV structures. However, manufacturers do not provide data for each PV cell or module variable condition. Only information taken at standard test conditions is shared in PV cell and module datasheets. With this data only, PV systems that will be exposed to meteorological and environmental effects and whose electrical energy produced is constantly changing cannot be modeled, energy production from those PV systems cannot be predicted, and many other calculations cannot be made with a realistic approach.^[^ ^12^ ^]^ Nevertheless, it is possible to accurately reflect the characteristics of PV systems, to imitate them, and to benefit from them at the maximum level with the correct modeling of these systems. These systems can be represented electrically with electrical equivalent circuits and modeled mathematically based on diode models.^[^ ^13^ ^]^ The extraction of the parameters of PV models is a new and challenging issue for solving the PV parameter extraction problem. With these parameters, reliable and highly accurate mathematical models are generated and used in many applications. Parameter extraction is a vibrant area of ongoing research. It is the increasing popularity of this problem that has paved the way for this study.

### 1.2 Literature Review and Research Gap

The recent papers on PV parameter extraction and its solution have been intensively reviewed. A detailed literature consisting of 30 valuable papers, including meta‐heuristic algorithms, is given in **Table** **1**. Based on the literature review, namely, the INFO algorithm,^[^ ^14^ ^]^ atomic orbital search algorithm,^[^ ^15^ ^]^ improved electromagnetism‐like mechanism algorithm,^[^ ^16^ ^]^ northern goshawk optimization algorithm,^[^ ^17^ ^]^ improved queuing search optimization algorithm based on differential evaluation,^[^ ^18^ ^]^ fractional Henon chaotic Harris Hawks optimization,^[^ ^19^ ^]^ hunter‐prey algorithm,^[^ ^20^ ^]^ robust niching optimization,^[^ ^21^ ^]^ wild horse optimizer,^[^ ^20^ ^]^ heap‐based optimizer,^[^ ^22^ ^]^ modified stochastic fractal search algorithm,^[^ ^23^ ^]^ circle search algorithm,^[^ ^24^ ^]^ orthogonal learning gradient‐based optimization,^[^ ^25^ ^]^ improved rao‐1 algorithm,^[^ ^26^ ^]^ improved political optimization algorithm,^[^ ^27^ ^]^ memory‐based improved gorilla troops optimizer,^[^ ^28^ ^]^ adaptive fractional‐order Archimedes optimization algorithm,^[^ ^29^ ^]^ multistrategy cuckoo search algorithm,^[^ ^30^ ^]^ whale optimizer with Nelder‐Mead simplex,^[^ ^31^ ^]^ Runge‐Kutta optimizer,^[^ ^32^ ^]^ turbulent flow of water‐based optimization,^[^ ^33^ ^]^ supply demand optimization,^[^ ^34^ ^]^ enhanced chaotic JAYA algorithm,^[^ ^35^ ^]^ modified teaching–learning based optimization,^[^ ^36^ ^]^ enhanced marine predators algorithm,^[^ ^37^ ^]^ hybrid African vultures–grey wolf optimizer,^[^ ^38^ ^]^ niche particle swarm optimization in parallel computing,^[^ ^39^ ^]^ simulated annealing optimization,^[^ ^40^ ^]^ enhanced ant lion optimizer,^[^ ^41^ ^]^ enhanced Lévy flight bat algorithm,^[^ ^42^ ^]^ and teaching–learning‐based artificial bee colony^[^ ^43^ ^]^ algorithms have been used for PV parameter extraction. The results of the parameter extraction problem solved with these meta‐heuristic algorithms are presented under two categories: the simulation and the sensitivity analysis. Both categories include algorithm information, PV brand, type description, number of series‐connected PV cells, PV test conditions, PV model information, and RMSE results. The RMSE is a popular metric used to measure the accuracy of a predictive model. Therefore, the RMSE formulas employed by the featured studies were collected in Table 1 for evaluation purposes. After a careful examination, one can see that there is a lack of uniformity in the definition of this popular metric. Instead, it is quite possible to see a range of formulas with new definitions and different calculation techniques besides the well‐known definition. However, in this article, the classic RMSE definition that was formulated in Section 2.4. is used throughout this study.

**Table 1:** Detailed literature review with simulation and sensitivity analysis results.

Algorithm	Simulation					Sensitivity				
		PV test conditions					PV test conditions			
	PV brand, type, and number of series‐connected PV cells (*N_s_*)	*T* [°C]	*G* [W/m^2^]	PV model	RMSE	PV brand, type, and number of cells in series (*N_s_*)	*T* [°C]	*G* [W/m^2^]	PV model	RMSE
INFO algorithm^[^ ^14^ ^]^	R.T.C France silicon *N_s_* = 1	33	1000	SDM DDM	9.8602E‐04 9.8248E‐04	BP 3170B Poly‐crystalline *N_s_* = 72	25	1000 800 600 400 200	SDM	3.8756E‐03 2.9243E‐03 2.2987E‐03 1.3578E‐03 6.8005E‐04
	Schutten Solar STM6‐40/36 Mono‐crystalline *N_s_* = 36	51	N/A	SDM DDM	1.7298E‐03 1.6949E‐03		0 25 50 75	1000	SDM	1.3595E‐02 3.8032E‐03 3.7733E‐03 3.6826E‐03
	Photowatt PWP 201 Poly‐crystalline *N_s_* = 36	45	1000	SDM DDM	2.3860E‐03 2.3860E‐03					
	Schutten Solar STP6‐120/36 Poly‐crystalline *N_s_* = 36	55	N/A	SDM DDM	1.6601E‐02 1.6601E‐02					
Atomic orbital search algorithm^[^ ^15^ ^]^	R.T.C France Silicon *N_s_* = 1	33	1000	SDM DDM TDM	7.7527E‐04 7.6059E‐04 7.9500E‐04	–				
	PVM752 GaAs Thin‐film *N_s_* = 1	25	1000	SDM DDM TDM	1.6184E‐04 1.7804E‐03 3.9041E‐04					
Improved electromagnetism‐like mechanism algorithm^[^ ^16^ ^]^	Schutten Solar STP6‐120/36 Poly‐crystalline *N_s_* = 36	55	N/A	SDM DDM TDM	1.1771E‐02 1.1339E‐02 1.2606E‐02	Shell SM‐55 Mono‐crystalline *N_s_* = 36	25	1000 800 600 400 200	SDM	1.2800E‐04 2.6500E‐04 2.4400E‐04 3.8829E‐04 2.4394E‐04
	Shell SM‐55 Mono‐crystalline *N_s_* = 36	25	1000	SDM DDM TDM	1.2800E‐04 4.5600E‐04 2.1100E‐03		20 40 60	1000	SDM	1.4088E‐04 1.4002E‐04 1.6529E‐04
	PVM 752 GaAs Thin‐film *N_s_* = 1	25	1000	SDM DDM TDM	1.2942E‐04 5.1286E‐05 1.6398E‐04					
Northern goshawk optimization algorithm^[^ ^17^ ^]^	Photowatt PWP 201 Poly‐crystalline *N_s_* = 36	25	1000	TDM	1.346E‐05	–				
	Kyocera‐KC200GT Multi‐crystalline *N_s_* = 54	25	1000	TDM	9.4174E‐05					
	Canadian Solar CS6K‐280 M Mono‐crystalline *N_s_* = 60	N/A	N/A	TDM	1.9520E‐04					
Improved queuing search optimization algorithm based on differential evaluation^[^ ^18^ ^]^	R.T.C France Silicon *N_s_* = 1	33	1000	SDM DDM	9.8602E‐04 9.8248E‐04	Siemens ST40 Thin‐film	25	1000 800 600 400 200	SDM	7.3409E‐04 7.7390E‐04 6.7403E‐04 6.3072E‐04 4.7720E‐04
	Photowatt PWP 201 Poly‐crystalline *N_s_* = 36	45	1000	SDM	2.4250E‐03		25 40 55 70	1000	SDM	7.3409E‐04 1.3214E‐03 1.8232E‐03 7.7771E‐04
	Schutten Solar STM6‐40/36 Mono‐crystalline *N_s_* = 36	51	N/A	SDM	1.7298E‐03	Shell SM‐55 Mono‐crystalline *N_s_* = 36	25	1000 800 600 400 200	SDM	1.1462E‐03 6.6857E‐04 8.2394E‐04 7.0760E‐04 3.2068E‐04
	Schutten Solar STP6‐120/36 Poly‐crystalline *N_s_* = 36	55	N/A	SDM	1.6600E‐02		25 40 70	1000	SDM	1.1462E‐03 3.7888E‐03 3.7803E‐03
Fractional Henon chaotic Harris Hawks optimization^[^ ^19^ ^]^	R.T.C France Silicon *N_s_* = 1	33	1000	SDM DDM TDM	3.8300E‐04 3.9400E‐04 2.4500E‐04					
	Photowatt PWP 201 Poly‐crystalline *N_s_* = 36	45	1000	SDM DDM TDM	7.4800E‐06 9.0300E‐06 7.8200E‐06					
Hunter‐prey algorithm^[^ ^20^ ^]^	R.T.C France Silicon *N_s_* = 1	33	1000	TDM	7.5674E‐04	Siemens ST40 Thin‐film	25	1000 800 600 400 200	TDM	3.7673E‐03 3.4500E‐03 9.9177E‐03 5.3642E‐03 1.9759E‐03
	Photowatt PWP 201 Poly‐crystalline *N_s_* = 36	45	1000	TDM	2.0465E‐03		25 40 55 70	1000	TDM	2.8801E‐2 7.0888E‐03 3.2023E‐03 6.4328E‐03
Robust niching optimization^[^ ^21^ ^]^	R.T.C France Silicon *N_s_* = 1	33	1000	SDM DDM TDM	9.7600E‐04 9.7200E‐04 9.7100E‐04	–				
	Shell SM‐55 Mono‐crystalline *N_s_* = 36	25	1000	SDM	3.3528E‐04					
	Kyocera‐KC200GT Multi‐crystalline *N_s_* = 54	25	1000	SDM	1.8213E‐02					
	SW255 Poly‐crystalline *N_s_* = 36	25	1000	SDM	1.4527E‐02					
Wild horse optimizer^[^ ^20^ ^]^	R.T.C France Silicon *N_s_* = 1	33	1000	TDM	7.5119E‐04	Siemens ST40 Thin‐film	25	1000 800 600 400 200	TDM	3.7673E‐03 3.4500E‐03 9.9177E‐03 5.3642E‐03 1.9759E‐03
	Photowatt PWP 201 Poly‐crystalline *N_s_* = 36	45	1000	TDM	2.0465E‐03		25 40 55 70	1000	TDM	2.8801E‐02 7.0888E‐03 3.1833E‐03 6.4328E‐03
Heap‐based optimizer^[^ ^22^ ^]^	R.T.C France Silicon *N_s_* = 1	33	1000	SDM DDM TDM	9.8869E‐04 1.0497E‐03 1.0705E‐03	–				
Modified stochastic fractal search algorithm^[^ ^23^ ^]^	R.T.C France Silicon *N_s_* = 1	33	1000	SDM DDM	9.8602E‐04 9.8248E‐04	Shell SM‐55 Mono‐crystalline *N_s_* = 36	25	1000 800 600 400 200	SDM	1.2147E‐02 2.4563E‐02 1.1178E‐02 6.0530E‐03 2.4990E‐03
	Photowatt PWP 201 Poly‐crystalline *N_s_* = 36	45	1000	SDM	2.4250E‐03		50 60	1000	SDM	1.0802E‐02 9.8330E‐03
							25	1000 800 600 400 200	DDM	1.2265E‐02 2.4601E‐02 1.1163E‐02 6.0020E‐03 2.4990E‐03
							50 60	1000	DDM	1.0259E‐02 9.7410E‐03
						Siemens ST40 Thin‐film	25	1000 800 600 400 200	SDM	1.8839E‐02 1.4542E‐02 1.2510E‐02 7.8730E‐03 7.6760E‐03
							50 60	1000	SDM	1.7495E‐02 1.9635E‐02
							25	1000 800 600 400 200	DDM	1.1863E‐02 1.3258E‐02 1.1097E‐02 6.9210E‐03 6.9190E‐03
							50 60	1000	DDM	1.5520E‐02 1.7964E‐02
Circle search algorithm^[^ ^24^ ^]^	Kyocera‐KC200GT Multi‐crystalline *N_s_* = 54	25	1000	TDM	3.2384E‐02	–				
	MSX‐60 Multi‐crystalline *N_s_* = 36	25	1000	TDM	1.3012E‐02					
	Canadian Solar CS6K‐280 M Monocrystalline *N_s_* = 60	25	1000	TDM	3.9602E‐02					
Orthogonal learning gradient‐based optimization^[^ ^25^ ^]^	R.T.C France Silicon *N_s_* = 1	33	1000	SDM DDM TDM	9.8602E‐04 9.8248E‐04 9.8249E‐04	Siemens ST40 Thin‐film	25	1000 800 600 400 200	SDM	7.3410E‐04 7.7390E‐04 6.7390E‐04 6.3072E‐04 4.7720E‐04
							25 40 50 70	1000	SDM	7.3409E‐04 1.3214E‐03 1.8232E‐03 7.7771E‐04
							25	1000 800 600 400 200	DDM	7.3410E‐04 7.7390E‐04 6.7403E‐04 6.3072E‐04 4.5593E‐04
							25 40 55 70	1000	DDM	7.3432E‐04 1.3214E‐03 1.6012E‐03 7.7771E‐04
						Kyocera KC200GT Multi‐crystalline *N_s_* = 54	25	1000 800 600 400 200	SDM	1.4083E‐03 1.3450E‐03 1.3529E‐03 1.3504E‐03 1.4353E‐03
							25 50 75	1000	SDM	1.4392E‐03 3.6831E‐03 2.8055E‐03
							25	1000 800 600 400 200	DDM	1.4540E‐03 1.3028E‐03 1.3534E‐03 1.3748E‐03 14355E‐03
							25 50 75	1000	DDM	1.3976E‐03 1.4438E‐03 2.0834E‐03
Improved rao‐1 algorithm^[^ ^26^ ^]^	R.T.C France Silicon *N_s_* = 1	33	1000	SDM DDM	9.8602E‐04 9.8233E‐04	–				
	Schutten Solar STM6‐40/36 Monocrystalline *N_s_* = 36	51	N/A	SDM	1.7321E‐03					
	Schutten Solar STP6‐120/36 Polycrystalline *N_s_* = 36	55	N/A	SDM	1.6602E‐02					
	Photowatt PWP 201 Poly‐crystalline *N_s_* = 36	45	1000	SDM	2.4251E‐03					
Improved political optimization algorithm^[^ ^27^ ^]^	R.T.C France Silicon *N_s_* = 1	33	1000	SDM DDM TDM	9.8602E‐04 9.8248E‐04 9.8248E‐04	Siemens ST40 Thin‐film	25	1000 800 600 400 200	SDM	7.3410E‐04 7.7390E‐04 6.7403E‐04 6.3072E‐04 4.7720E‐04
							25 40 50 70	1000	SDM	7.3409E‐04 1.3214E‐03 1.8232E‐03 7.7771E‐04
							25	1000 800 600 400 200	DDM	7.3479E‐04 7.8325E‐04 6.7656E‐04 6.3207E‐04 4.5950E‐04
							25 40 50 70	1000	DDM	7.4513E‐04 1.3263E‐03 1.6116E‐03 7.7776E‐04
						Shell SM‐55 Monocrystalline *N_s_* = 36	25	1000 800 600 400 200	SDM	1.1462E‐03 6.6858E‐03 8.2395E‐04 7.0760E‐04 5.2054E‐04
							25 40 60	1000	SDM	1.1462E‐03 3.7881E‐03 3.7803E‐03
							25	1000 800 600 400 200	DDM	1.1671E‐03 6.6857E‐04 8.2394E‐04 6.0706E‐04 5.2054E‐04
							25 40 60	1000	DDM	1.1489E‐03 3.7888E‐03 3.7803E‐03
						Kyocera‐KC200GT Multi‐crystalline *N_s_* = 54	25	1000 800 600 400 200	SDM	1.6689E‐03 2.4384E‐03 1.2976E‐03 1.4262E‐03 1.4221E‐03
							25 50 75	1000	SDM	1.5390E‐03 2.7465E‐03 4.4729E‐03
							25	1000 800 600 400 200	DDM	2.0811E‐03 1.5793E‐03 1.3039E‐03 1.4262E‐03 1.4167E‐03
							25 50 75	1000	DDM	2.0811E‐03 2.7474E‐03 4.4729E‐03
Memory‐based improved gorilla troops optimizer^[^ ^28^ ^]^	R.T.C France Silicon *N_s_* = 1	33	1000	SDM DDM TDM	9.8602E‐04 9.8248E‐04 9.8248E‐04	Kyocera‐KC200GT Multi‐crystalline *N_s_* = 54	25	1000 800 600 400 200	SDM	1.5390E‐03 1.6309E‐03 1.2976E‐03 1.4261E‐03 1.4184E‐03
	Photowatt PWP 201 Poly‐crystalline *N_s_* = 36	45	1000	SDM	2.4250E‐03		25 50 75	1000	SDM	4.4729E03 2.7465E‐03 1.5390E‐03
	Schutten Solar STM6‐40/36 Mono‐crystalline *N_s_* = 36	51	N/A	SDM	1.7298E‐03	Shell SM‐55 Mono‐crystalline *N_s_* = 36	25	1000 800 600 400 200	SDM	1.1462E‐03 6.6857E‐04 8.2394E‐04 7.0760E‐04 3.2068E‐04
	Schutten Solar STP6‐120/36 Poly‐crystalline *N_s_* = 36	55	N/A	SDM	1.6600E‐03		25 40 60	1000	SDM	1.1462E‐03 3.7888E‐03 3.7803E‐03
						Siemens ST40 Thin‐film	25	1000 800 600 400 200	SDM	7.3409E‐04 7.7390E‐04 6.7403E‐04 6.3072E‐04 4.4772E‐04
							25 40 55 70	1000	SDM	7.3409E‐04 1.3214E‐03 1.8232E‐03 7.7771E‐04
Adaptive fractional‐order Archimedes optimization algorithm ^[^ ^29^ ^]^	R.T.C France Silicon *N_s_* = 1	33	1000	SDM DDM	7.7301E‐04 7.5767E‐04	Canadian Solar CS6P‐240P Mono‐crystalline *N_s_* = 60	25 45.92 51.91 43.95 40.05 37.32	1000 673.5 580.3 347.8 246.65 109.2	DDM	4.9497E‐03 2.8487E‐02 2.6738E‐02 1.5142E‐02 1.2599E‐02 3.5601E‐03
	SLP080 Multi‐crystalline *N_s_* = 36	25	1000	SDM DDM	2.1682E‐02 2.1652E‐02		45 60 75	1000	DDM	1.9842E‐03 2.9045E‐05 1.5386E‐06
Multi‐strategy cuckoo search algorithm^[^ ^30^ ^]^	R.T.C France Silicon *N_s_* = 1	33	1000	SDM DDM	9.8602E‐04 9.8248E‐04	–				
	Schutten Solar STM6‐40/36 Mono‐crystalline *N_s_* = 36	51	N/A	SDM	1.7298E‐03					
	Schutten Solar STP6‐120/36 Poly‐crystalline *N_s_* = 36	55	N/A	SDM	1.6601E‐02					
	Photowatt PWP 201 Poly‐crystalline *N_s_* = 36	45	1000	SDM	2.4251E‐03					
Whale optimizer with Nelder‐Mead simplex^[^ ^31^ ^]^	R.T.C France Silicon *N_s_* = 1	33	1000	SDM DDM TDM	9.8602E‐04 9.8248E‐04 9.8248E‐04	Siemens ST40 Thin‐film	25	1000 800 600 400 200	SDM	7.3410E‐04 7.7390E‐04 6.7403E‐04 6.3072E‐04 4.7720E‐04
	Photowatt PWP 201 Poly‐crystalline *N_s_* = 36	45	1000	SDM	2.4251E‐03		25 40 50 70	1000	SDM	7.3409E‐04 1.3214E‐03 1.8232E‐03 7.7710E‐04
							25	1000 800 600 400 200	DDM	7.3409E‐04 7.7390E‐04 6.7403E‐04 6.3072E‐04 4.5449E‐04
							25 40 50 70	1000	DDM	7.3409E‐04 1.3214E‐03 1.5510E‐03 7.7771E‐04
						Shell SM‐55 Mono‐crystalline *N_s_* = 36	25	1000 800 600 400 200	SDM	7.0760E‐04 1.1462E‐03 6.6858E‐04 8.2395E‐04 7.0760E‐04
							25 40 60	1000	SDM	1.1462E‐03 3.7881E‐03 3.7803E‐03
							25	1000 800 600 400 200	DDM	1.1462E‐03 6.6857E‐04 8.2394E‐04 5.9930E‐04 5.2054E‐04
							25 40 60	1000	DDM	1.1462E‐03 3.7888E‐03 3.7803E‐03
						Kyocera‐KC200GT Multi‐crystalline *N_s_* = 54	25	1000 800 600 400 200	SDM	1.5390E‐03 1.6309E‐03 1.2976E‐03 1.4262E‐03 1.4184E‐03
							25 50 75	1000	SDM	1.5390E‐03 2.7465E‐03 4.4729E‐03
							25	1000 800 600 400 200	DDM	1.5389E‐03 1.5058E‐03 1.2976E‐03 1.4262E‐03 1.4092E‐03
							25 50 75	1000	DDM	1.5389E‐03 2.7465E‐03 4.4729E‐03
Rung‐Kutta optimizer^[^ ^32^ ^]^	R.T.C France Silicon *N_s_* = 1	33	1000	SDM DDM	7.7301E‐04 7.4653E‐04	Kyocera‐KC200GT Multi‐crystalline *N_s_* = 54	25 45 75	1000	DDM	3.3228E‐03 3.2480E‐03 3.1905E‐03
						NU‐(Q250W2) Mono‐crystalline Silicon *N_s_* = 60	25 55 70	1000	DDM	2.4395E‐04 3.7118E‐04 2.0770E‐04
						Pytagoras Solar Large PVGU Window N/A *N_s_* = 90	25 40 60	1000	DDM	2.8506E‐03 2.6777E‐03 2.5875E‐03
Turbulent flow of water‐based optimization^[^ ^33^ ^]^	R.T.C France Silicon *N_s_* = 1	33	1000	SDM DDM TDM	2.5278‐E05 2.5100E‐05 2.5100E‐05	–				
Supply demand optimization^[^ ^34^ ^]^	Schutten Solar STM6‐40/36 Mono‐crystalline *N_s_* = 36	51	N/A	SDM DDM TDM	1.7300E‐03 1.7252E‐03 1.7018E‐03	Kyocera‐KC200GT Multi‐crystalline *N_s_* = 54	25	1000 800 600 400 200	SDM	6.3665E‐04 6.4548E‐04 6.3254E‐04 2.4302E‐04 2.3845E‐04
	Schutten Solar STP6‐120/36 Poly‐crystalline *N_s_* = 36	55	N/A	SDM DDM TDM	1.6601E‐02 1.6601E‐02 1.6620E‐02		25 50 75	1000	SDM	6.3665E‐04 1.5622E‐03 3.9212E‐02
	Photowatt PWP 201 Poly‐crystalline *N_s_* = 36	45	1000	SDM DDM TDM	2.4250E‐03 2.4250E‐03 1.8320E‐03					
Enhanced chaotic JAYA algorithm^[^ ^35^ ^]^	R.T.C France Silicon *N_s_* = 1	33	1000	SDM DDM	9.8602E‐04 9.8269E‐04	–				
	Schutten Solar STP6‐120/36 Poly‐crystalline *N_s_* = 36	55	N/A	SDM	1.6285E‐02					
	Schutten Solar STM6‐40/36 Mono‐crystalline *N_s_* = 36	51	N/A	SDM	1.7248E‐03					
Modified teaching–learning based optimization^[^ ^36^ ^]^	R.T.C France Silicon *N_s_* = 1	33	1000	SDM DDM	9.8602E‐04 9.8248E‐04	Siemens ST40 Thin‐film	25	1000 800 600 400 200	SDM	7.3409E‐04 7.7390E‐04 6.7403E‐04 6.3072E‐04 4.7720E‐04
	Photowatt PWP 201 Poly‐crystalline *N_s_* = 36	45	1000	SDM	2.4250E‐03		40 55 70	1000	SDM	1.3214E‐03 1.8232E‐03 7.7771E‐04
	Schutten Solar STM6‐40/36 Mono‐crystalline *N_s_* = 36	51	N/A	SDM	1.7298E‐03	Shell SM‐55 Monocrystalline *N_s_* = 36	25	1000 800 600 400 200	SDM	1.1462E‐03 6.6857E‐04 8.2394E‐04 7.0760E‐04 3.2068E‐04
	Schutten Solar STP6‐120/36 Poly‐crystalline *N_s_* = 36	55	N/A	SDM	1.6600E‐02		40 60	1000	SDM	3.7888E‐03 3.7803E‐03
Enhanced marine predators algorithm^[^ ^37^ ^]^	R.T.C France Silicon *N_s_* = 1	33	1000	SDM DDM	7.7301E‐04 7.4396E‐04	Canadian Solar CS69‐240P Poly‐crystalline *N_s_* = 60	45.92 51.91 43.95 40.05 37.32	673.5 580.3 347.8 246.65 109.2	DDM	2.8491E‐02 2.6738E‐02 1.5227E‐02 1.2647E‐02 3.5610E‐03
Hybrid African vultures–grey wolf optimizer^[^ ^38^ ^]^	Kyocera‐KC200GT Multicrystalline *N_s_* = 54	25	1000	TDM	8.4750E‐13	–				
	MSX‐60 Multi‐crystalline *N_s_* = 36	25	1000	TDM	7.412E‐12					
Niche particle swarm optimization in parallel computing ^[^ ^39^ ^]^	R.T.C France Silicon *N_s_* = 1	33	1000	SDM DDM	9.8856E‐04 9.8208E‐04	–				
	Photowatt PWP 201 Poly‐crystalline *N_s_* = 36	45	1000	SDM	2.4251E‐03					
Simulated annealing optimization^[^ ^40^ ^]^	R.T.C France Silicon *N_s_* = 1	33	1000	SDM	1.5727E‐17	–				
	Photowatt PWP 201 Poly‐crystalline *N_s_* = 36	45	1000	SDM	6.8284E‐17					
	Schutten Solar STP6‐120/36 Poly‐crystalline *N_s_* = 36	55	N/A	SDM	3.7376E‐16					
	Schutten Solar STM6‐40/36 Mono‐crystalline *N_s_* = 36	51	N/A	SDM	4.9651E‐17					
Enhanced ant lion optimizer^[^ ^41^ ^]^	R.T.C France Silicon *N_s_* = 1	33	1000	SDM DDM	9.8602E‐04 9.8247E‐04	Siemens ST40 Thin‐film	25	1000 800 600 400	SDM	7.3410E‐04 7.7391E‐04 6.7404E‐04 6.3072E‐04
	Photowatt PWP 201 Poly‐crystalline *N_s_* = 36	45	1000	SDM	2.4248E‐03		40 50 70	1000	SDM	1.3214E‐03 1.8233E‐03 7.7772E‐04
							25	1000 800 600 400	DDM	7.3410E‐04 7.7391E‐04 6.7404E‐04 6.3072E‐04
							40 50 70	1000	DDM	1.3214E‐03 1.5732E‐03 7.7772E‐04
						Shell SM‐55 Mono‐crystalline *N_s_* = 36	25	1000 800 600 400	SDM	1.1462E‐03 6.6858E‐04 8.2395E‐04 7.0661E‐04
							40 60	1000	SDM	3.7888E‐03 3.7804E‐03
							25	1000 800 600 400	DDM	1.1462E‐03 6.6858E‐04 8.2395E‐04 5.9929E‐04
							40 60	1000	DDM	3.7888E‐03 3.7804E‐03
						Kyocera‐KC200GT Multi‐crystalline *N_s_* = 54	25	1000 800 600 400	SDM	1.5390E‐03 1.6310E‐03 1.2977E‐03 1.4262E‐03
							40 60	1000	SDM	2.7465E‐03 4.4729E‐03
							25	1000 800 600 400	DDM	1.5389E‐03 1.5052E‐03 1.2977E‐03 1.4262E‐03
							40 60	1000	DDM	2.7465E‐03 4.4729E‐03
Enhanced Lévy flight bat algorithm^[^ ^42^ ^]^	R.T.C France Silicon *N_s_* = 1	33	1000	SDM DDM	9.8602E‐04 9.8248E‐04	–				
	Schutten Solar STM6‐40/36 Mono‐crystalline *N_s_* = 36	51	N/A	SDM DDM	1.7298E‐03 1.6884E‐03					
	PVM 752 GaAs Thin‐film *N_s_* = 1	25	1000	SDM DDM	2.2780E‐04 1.2488E‐04					
Teaching–learning‐based artificial bee colony ^[^ ^43^ ^]^	R.T.C France Silicon *N_s_* = 1	33	1000	SDM DDM	9.8602E‐04 9.8414E‐04	–				
	Photowatt PWP 201 Poly‐crystalline *N_s_* = 36	45	1000	SDM	2.4250E‐03					

It can be concluded that, when the detailed literature review is given in Table 1, there is no dominant and leading algorithm that produces the most successful results on a cell and module basis, also on a PV model basis. The no‐free‐lunch theorem explains and supports this issue. According to this theorem, there is no single/unique algorithm that solves or is capable of solving all optimization problems by reaching the best/global result. An algorithm may produce superior results compared to its competitors on a specific problem and perform unrivaled. However, there is no guarantee that it will achieve the same success on different issues other than that particular problem.^[^ ^44^ ^]^ Parameter extraction of PV models to produce reliable and accurate mathematical models is a current and challenging issue. Based on the no‐free‐lunch theorem and the results of our literature survey, no single algorithm is unrivaled in all problems for all cells and modules. PV parameter extraction is a vibrant area of ongoing research. Therefore, in this paper, the PV parameter extraction problem is studied. In this study, parameter extraction is performed with the WLS, the spider wasp optimizer (SWO), the shrimp and goby association search algorithm (SGA), the reversible elementary cellular automata algorithm (RECAA), the fennec fox optimization (FFA), the Kepler optimization algorithm (KOA), and the rime optimization algorithm (RIME) algorithms, and the results are analyzed in detail.

### 1.3 Contributions

The contributions of this article are given below.

- The PV parameter extraction problem is studied, and WLS, SWO, SGA, RECAA, FFA, and RIME algorithms, except KOA, are used for the first time in this study.
- Two PV cells and six PV modules are selected. Various PV structures including silicon, mono‐crystalline, polycrystalline, multicrystalline, and thin film were used to create a large test area for testing the algorithms.
- The parameter extraction performance of the algorithms is tested with the experimental measurement data of these PV cells and modules modeled as single‐diode model (SDM), double diode model (DDM), and triple‐diode model (TDM).
- The success of the algorithms was evaluated with evaluation metrics and two statistical tests, the Friedman test and the Wilcoxon signed‐rank test. These statistical tests proved the statistical significance and superiority of the WLS algorithm over the other algorithms.
- The successful sensitivity analysis showed that the WLS algorithm is a reliable alternative for PV parameter extraction.

### 1.4 Organization of the Article

The article is presented in five main sections. Section 1 is the introduction. In this section, the motivation for this work, a detailed literature review of the problem, our contributions, and the organization of the article are given. In Section 2, the PV parameter extraction problem is defined, PV models and objective functions are given. Section 3 describes the solution tools for this problem. The WLS algorithm is explained in detail through its mathematical background. SWO, SGA, RECAA, FFA, KOA, and RIME algorithms are summarized. The results obtained by solving the defined problem with the selected algorithms are presented in Section 4. In this section, the results are evaluated and compared with evaluation metrics and statistical methods. In addition, the consistency of the results is reinforced with sensitivity analysis. In the last section, Section 5, the conclusions are presented.

## 2 Definition of the PV Parameter Extraction Problem

In this article, the PV parameter extraction problem is studied. SDM, DDM and TDM models of PV cells and modules and the objective function of the problem are given in the subsections.

### 2.1 Equivalent Circuit Structure of SDM

**Figure 1:**
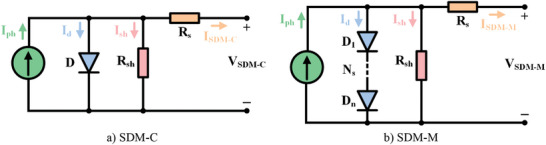
The equivalent circuit structure of SDM.

PV cells and modules are often modeled with SDM due to its simple structure. An SDM based on PV cell (SDM‐C) is shown in **Figure** **1**a. This equivalent circuit model consists of, from left to right, a current source (*I*_ph_) representing the DC current generated due to radiation, a diode (*D*) modeling the PN junction, a shunt resistance (*R*_sh_) corresponding to the power losses and a series resistance (*R*_s_). The shunt resistance reflects the leakage currents in the PN junction and the series resistance reflects the electrode resistance and the resistance effects of the contact surfaces of the components. The PV cell current for SDM‐C according to Kirchhoff current law, diode current, junction thermal voltage, shunt resistance current, and PV cell current are given in Equations (1)–(5), respectively. Similarly, an SDM based on PV module (SDM‐M) is given in Figure 1b. SDM‐M has only series connected cells. The PV module current is given in Equation (6). Solving of the problem for SDM means estimation of five parameters, namely *I*_ph_, *I*_o_, *α*, *R*_s_, and *R*_sh_.^[^ ^45^, ^46^, ^47^ ^]^

(1) $$I_{\mathrm{SDM}-C}=I_{\mathrm{ph}}-I_{d}-I_{\mathrm{sh}}$$

(2) $$I_{d}=I_{o}\left[e^{\left(\frac{V_{\mathrm{SDM}-C}+R_{s}\;I_{\mathrm{SDM}-C}}{\alpha \;V_{t}}\right)}-1\right]$$

(3) $$V_{t}=\frac{k\;T_{j}}{q}$$

(4) $$I_{\mathrm{sh}}=\frac{V_{\mathrm{SDM}-C}+R_{s}\;I_{\mathrm{SDM}-C}}{R_{\mathrm{sh}}}$$

(5) $$\begin{array}{c} I_{\mathrm{SDM}-C}=I_{\mathrm{ph}}-I_{o}\left[e^{\left(\frac{V_{\mathrm{SDM}-C}+R_{s}\;I_{\mathrm{SDM}-C}}{\alpha \;V_{t}}\right)}-1\right]-\frac{V_{\mathrm{SDM}-C}+R_{s}\;I_{\mathrm{SDM}-C}}{R_{\mathrm{sh}}} \end{array}$$

(6) $$\begin{array}{ccc} I_{\mathrm{SDM}-M} & = & I_{\mathrm{ph}}-I_{o}\left[e^{\left(\frac{V_{\mathrm{SDM}-M}+N_{s}\;R_{s}\;I_{\mathrm{SDM}-M}}{\alpha \;V_{t}\;N_{s}}\right)}-1\right]\\ & & -\;\frac{V_{\mathrm{SDM}-M}+N_{s}\;R_{s}\;I_{\mathrm{SDM}-M}}{R_{\mathrm{sh}}\;N_{s}} \end{array}$$

where, *I*_SDM − *C*_ and *V*_SDM − *C*_ are the output current and voltage of PV cell, respectively, *I*_ph_, *I*_d_, *I*_sh_, and *I*_o_ are the photogenerated current, diode current, shunt resistance current, and diode reverse saturation current, respectively, *R*_s_ and *R*_sh_ the series and shunt resistances, *α* is the ideality factor of diode, *V*_t_ is the junction thermal voltage, *k* is the Boltzmann constant, *T*_j_ is the junction temperature in Kelvin, *q* is the electron charge, *I*_SDM − M_ and *V*_SDM − M_ are the output current and voltage of PV module, respectively, and *N*_s_ is the number of series‐connected PV cells.

### 2.2 Equivalent Circuit Structure of DDM

**Figure 2:**
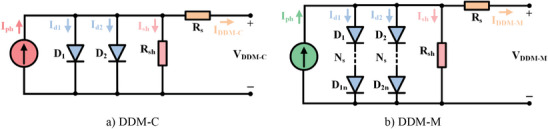
The equivalent circuit structure of DDM.

In addition to modeling with SDM, for a more realistic solar circuit, PV cells and modules are modeled with DDM. A DDM based on PV cell (DDM‐C) is shown in **Figure** **2**a. While there is one diode in SDM, an additional diode (*D*_2_) is used in parallel with this diode in this equivalent circuit model. The purpose of this diode is to take into account the effect of recombination losses and to reflect the non‐ideal effect of solar cells. The PV cell current for DDM‐C according to Kirchhoff current law, 1^st^ diode current, 2^nd^ diode current, shunt resistance current, and PV cell current are given in Equations (7)–(11) respectively. A DDM based on PV module (DDM‐M) is given in Figure 2b. The PV module current is given in Equation (12). There is no parallel connected cell in DDM‐M. Solving of problem for DDM means seven parameters to be estimated, namely *I*_ph_, *I*_o1_, *I*_o2_, *α*_1_, *α*_2_, *R*_s_, and *R*_sh_.^[^ ^32^, ^48^, ^49^ ^]^

(7) $$I_{\mathrm{DDM}-C}=I_{\mathrm{ph}}-I_{d1}-I_{d2}-I_{\mathrm{sh}}$$

(8) $$I_{d1}=I_{o1}\left(e^{\left(\frac{V_{\mathrm{DDM}-C}+R_{s}\;I_{\mathrm{DDM}-C}}{\alpha_{1}\;V_{t}}\right)}-1\right)$$

(9) $$I_{d2}=I_{o2}\left(e^{\left(\frac{V_{\mathrm{DDM}-C}+R_{s}\;I_{\mathrm{DDM}-C}}{\alpha_{2}\;V_{t}}\right)}-1\right)$$

(10) $$I_{\mathrm{sh}}=\frac{V_{\mathrm{DDM}-C}+R_{s}\;I_{\mathrm{DDM}-C}}{R_{\mathrm{sh}}}$$

(11) $$\begin{array}{ccc} I_{\mathrm{DDM}-C} & = & I_{\mathrm{ph}}-I_{o1}\left[e^{\left(\frac{V_{\mathrm{DDM}-C}+R_{s}\;I_{\mathrm{DDM}-C}}{\alpha_{1}\;V_{t}}\right)}-1\right]\\ & & -\;I_{o2}\left[e^{\left(\frac{V_{\mathrm{DDM}-C}+R_{s}\;I_{\mathrm{DDM}-C}}{\alpha_{2}\;V_{t}}\right)}-1\right]-\frac{V_{\mathrm{DDM}-C}+R_{s}\;I_{\mathrm{DDM}-C}}{R_{\mathrm{sh}}} \end{array}$$

(12) $$\begin{array}{ccc} I_{\mathrm{DDM}-M} & = & I_{\mathrm{ph}}-I_{o1}\left[e^{\left(\frac{V_{\mathrm{DDM}-M}+N_{s}\;R_{s}\;I_{\mathrm{DDM}-M}}{\alpha_{1}\;V_{t}\;N_{s}}\right)}-1\right]\\ & & -\;I_{o2}\left[e^{\left(\frac{V_{\mathrm{DDM}-M}+N_{s}\;R_{s}\;I_{\mathrm{DDM}-M}}{\alpha_{2}\;V_{t}\;N_{s}}\right)}-1\right]\\ & & -\;\frac{V_{\mathrm{DDM}-M}+N_{s}\;R_{s}\;I_{\mathrm{DDM}-M}}{R_{\mathrm{sh}}\;N_{s}} \end{array}$$

where, *I*_DDM − *C*_ and *V*_DDM − *C*_ are the output current and voltage of PV cell, respectively, *I*_d1_ and *I*_d2_ are the 1^st^ and 2^nd^ diode currents, *I*_o1_ and *I*_o2_ are the diode saturation currents, *α*_1_ and *α*_2_ are the 1^st^ and 2^nd^ diode ideality factors, and *I*_DDM − *M*_ and *V*_DDM − *M*_ are the output current and voltage of PV module, respectively.

### 2.3 Equivalent Circuit Structure of TDM

**Figure 3:**
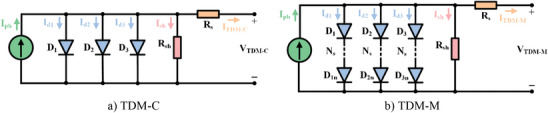
The equivalent circuit structure of TDM.

A TDM based on PV cell (TDM‐C), which is popular in industrial applications, is shown in **Figure** **3**a. While there are two diodes in the DDM, in this equivalent circuit model, a third diode (*D*_3_) is added in parallel to those two diodes. In this model, the defect area/region is taken into account to reflect the effect of leakage and recombination losses in this region. The PV cell current for TDM‐C according to Kirchhoff current law, 1^st^ diode current, 2^nd^ diode current, 3^rd^ diode current, shunt resistance current, and PV cell current are given in Equations (13)–(18), respectively. A TDM based on PV module (TDM‐M) is given in Figure 3b. The PV module current is given in Equation (19). It is assumed that only series connected cells are included in TDM‐M and no parallel connected cells are included. There are nine parameters to be estimated for TDM, namely *I*_ph_, *I*_o1_, *I*_o2_, *I*_o3_, *α*_1_, *α*_2_, *α*_3_, *R*_s_, and *R*_sh_.^[^ ^50^, ^51^, ^52^ ^]^

(13) $$I_{\mathrm{TDM}-C}=I_{\mathrm{ph}}\;-I_{d1}-I_{d2}-I_{d3}-I_{\mathrm{sh}}$$

(14) $$I_{d1}=I_{o1}\left(e^{\left(\frac{V_{\mathrm{TDM}-C}+R_{s}\;I_{\mathrm{TDM}-C}}{\alpha_{1}\;V_{t}}\right)}-1\right)$$

(15) $$I_{d2}=I_{o2}\left(e^{\left(\frac{V_{\mathrm{TDM}-C}+R_{s}\;I_{\mathrm{TDM}-C}}{\alpha_{2}\;V_{t}}\right)}-1\right)$$

(16) $$I_{d3}=I_{o3}\left(e^{\left(\frac{V_{\mathrm{TDM}-C}+R_{s}\;I_{\mathrm{TDM}-C}}{\alpha_{3}\;V_{t}}\right)}-1\right)$$

(17) $$I_{\mathrm{sh}}=\frac{V_{\mathrm{TDM}-C}+R_{s}\;I_{\mathrm{TDM}-C}}{R_{\mathrm{sh}}}$$

(18) $$\begin{array}{ccc} I_{\mathrm{TDM}-C} & = & I_{\mathrm{ph}}-I_{o1}\left[e^{\left(\frac{V_{\mathrm{TDM}-C}+R_{s}\;I_{\mathrm{TDM}-C}}{\alpha_{1}\;V_{t}}\right)}-1\right]\\ & & -\;I_{o2}\left[e^{\left(\frac{V_{\mathrm{TDM}-C}+R_{s}\;I_{\mathrm{TDM}-C}}{\alpha_{2}\;V_{t}}\right)}-1\right]\\ & & -\;I_{o3}\left[e^{\left(\frac{V_{\mathrm{TDM}-C}+R_{s}\;I_{\mathrm{TDM}-C}}{\alpha_{3}\;V_{t}}\right)}-1\right]-\frac{V_{\mathrm{TDM}-C}+R_{s}\;I_{\mathrm{TDM}-C}}{R_{\mathrm{sh}}} \end{array}$$

(19) $$\begin{array}{ccc} I_{\mathrm{TDM}-M} & = & I_{\mathrm{ph}}-I_{o1}\left[e^{\left(\frac{V_{\mathrm{TDM}-M}+N_{s}\;R_{s}\;I_{\mathrm{TDM}-M}}{\alpha_{1}\;V_{t}\;N_{s}}\right)}-1\right]\\ & & -\;I_{o2}\left[e^{\left(\frac{V_{\mathrm{TDM}-M}+N_{s}\;R_{s}\;I_{\mathrm{TDM}-M}}{\alpha_{2}\;V_{t}\;N_{s}}\right)}-1\right]\\ & & -\;I_{o3}\left[e^{\left(\frac{V_{\mathrm{TDM}-M}+N_{s}\;R_{s}\;I_{\mathrm{TDM}-M}}{\alpha_{3}\;V_{t}\;N_{s}}\right)}-1\right]\\ & & -\;\frac{V_{\mathrm{TDM}-M}+N_{s}\;R_{s}\;I_{\mathrm{TDM}-M}}{R_{\mathrm{sh}}\;N_{s}} \end{array}$$

where, *I*_TDM − *C*_ and *V*_TDM − *C*_ are the output current and voltage of PV cell, respectively, *I*_d3_ is the 3^rd^ diode current, *I*_*o*3_ is the diode reverse saturation current, *α*_3_ is the 3^rd^ diode ideality factor, and *I*_TDM − *M*_ and *V*_TDM − *M*_ are the output current and voltage of PV module, respectively.

### 2.4 Objective Function

The problem of this article is the PV parameter extraction and the solution tools of this optimization problem are WLS, SWO, SGA, RECAA, FFA, KOA, and RIME algorithms. PV cells and modules are modeled with SDM, DDM and TDM equivalent circuit models and it is aimed to obtain the optimal parameters with these seven algorithms. For this reason, the SDM, DDM and TDM functions and decision variables of PV cells and modules are given in Equation (20). In PV parameter extraction, experimentally measured current and voltage data of PV cells and modules under certain irradiance and temperature conditions are used. As seen in Equation (20), the difference between the current data estimated by our algorithms and the experimentally measured current data is expressed as a function of each model. Hence, the root mean square error (RMSE), which is the objective function of our problem, is given in Equation (21). The optimization process aims to minimize the difference between the estimated and the actual experimental data in order to extract the parameters.

(20) $$\begin{array}{c} f\left(x\right)=\left\{\begin{array}{c} f\left(x\right)=\left(I_{e}\right)-\left(I_{m}\right)\\ f_{\mathrm{SDM}-C}\left(x\right)=\left(I_{\mathrm{ph}}-I_{o}\left[e^{\left(\frac{V_{\mathrm{SDM}-C}+R_{s}\;I_{\mathrm{SDM}-C}}{\alpha \;V_{t}}\right)}-1\right]-\frac{V_{\mathrm{SDM}-C}+R_{s}\;I_{\mathrm{SDM}-C}}{R_{\mathrm{sh}}}\right)-\left(I_{\mathrm{SDM}-C_{m}}\right)\\ f_{\mathrm{SDM}-M}\left(x\right)=\left(I_{\mathrm{ph}}-I_{o}\left[e^{\left(\frac{V_{\mathrm{SDM}-M}+N_{s}\;R_{s}\;I_{\mathrm{SDM}-M}}{\alpha \;V_{t}\;N_{s}}\right)}-1\right]-\frac{V_{\mathrm{SDM}-M}+N_{s}\;R_{s}\;I_{\mathrm{SDM}-M}}{R_{sh}\;N_{s}}\right)-\left(I_{\mathrm{SDM}-M_{m}}\right)\\ f_{\mathrm{DDM}-C}\left(x\right)=\left(I_{\mathrm{ph}}-I_{o1}\left[e^{\left(\frac{V_{\mathrm{DDM}-C}+R_{s}\;I_{\mathrm{DDM}-C}}{\alpha_{1}\;V_{t}}\right)}-1\right]-I_{o2}\left[e^{\left(\frac{V_{\mathrm{DDM}-C}+R_{s}\;I_{\mathrm{DDM}-C}}{\alpha_{2}\;V_{t}}\right)}-1\right]-\frac{V_{\mathrm{DDM}-C}+R_{s}\;I_{\mathrm{DDM}-C}}{R_{\mathrm{sh}}}\right)-\left(I_{\mathrm{DDM}-C_{m}}\right)\\ f_{\mathrm{DDM}-M}\left(x\right)=\left(I_{\mathrm{ph}}-I_{o1}\left[e^{\left(\frac{V_{\mathrm{DDM}-M}+N_{s}\;R_{s}\;I_{\mathrm{DDM}-M}}{\alpha_{1}\;V_{t}\;N_{s}}\right)}-1\right]-I_{o2}\left[e^{\left(\frac{V_{\mathrm{DDM}-M}+N_{s}\;R_{s}\;I_{\mathrm{DDM}-M}}{\alpha_{2}\;V_{t}\;N_{s}}\right)}-1\right]-\frac{V_{\mathrm{DDM}-M}+N_{s}\;R_{s}\;I_{\mathrm{DDM}-M}}{R_{\mathrm{sh}}\;N_{s}}\right)-\left(I_{\mathrm{DDM}-M_{m}}\right)\\ f_{\mathrm{TDM}-C}\left(x\right)=\begin{array}{c} \left(I_{\mathrm{ph}}-I_{o1}\left[e^{\left(\frac{V_{\mathrm{TDM}-C}+R_{s}\;I_{\mathrm{TDM}-C}}{\alpha_{1}\;V_{t}}\right)}-1\right]-I_{o2}\left[e^{\left(\frac{V_{\mathrm{TDM}-C}+R_{s}\;I_{\mathrm{TDM}-C}}{\alpha_{2}\;V_{t}}\right)}-1\right]-\cdots \right.\\ \left.I_{o3}\left[e^{\left(\frac{V_{\mathrm{TDM}-C}+R_{s}\;I_{\mathrm{TDM}-C}}{\alpha_{3}\;V_{t}}\right)}-1\right]-\frac{V_{\mathrm{TDM}-C}+R_{s}\;I_{\mathrm{TDM}-C}}{R_{\mathrm{sh}}}\right)-\left(I_{\mathrm{TDM}-C_{m}}\right) \end{array}\\ f_{\mathrm{TDM}-M}\left(x\right)=\begin{array}{c} \left(I_{\mathrm{ph}}-I_{o1}\left[e^{\left(\frac{V_{\mathrm{TDM}-M}+N_{s}\;R_{s}\;I_{\mathrm{TDM}-M}}{\alpha_{1}\;V_{t}\;N_{s}}\right)}-1\right]-I_{o2}\left[e^{\left(\frac{V_{\mathrm{TDM}-M}+N_{s}\;R_{s}\;I_{\mathrm{TDM}-M}}{\alpha_{2}\;V_{t}\;N_{s}}\right)}-1\right]-\cdots \right.\\ I_{o3}\left[e^{\left(\frac{V_{\mathrm{TDM}-M}+N_{s}\;R_{s}\;I_{\mathrm{TDM}-M}}{\alpha_{3}\;V_{t}\;N_{s}}\right)}-1\right]\left.-\frac{V_{\mathrm{TDM}-M}+N_{s}\;R_{s}\;I_{\mathrm{TDM}-M}}{R_{\mathrm{sh}}\;N_{s}}\right)-\left(I_{\mathrm{TDM}-M_{m}}\right) \end{array} \end{array}\right.\\ x=\left\{\begin{array}{c} x_{\mathrm{SDM}-C}=I_{\mathrm{ph}},\;I_{o},\;\alpha ,\;R_{s},\;R_{\mathrm{sh}}\\ x_{\mathrm{SDM}-M}=I_{\mathrm{ph}},\;I_{o},\;\alpha ,\;R_{s},\;R_{\mathrm{sh}}\\ x_{\mathrm{DDM}-C}=I_{\mathrm{ph}},\;I_{o1},\;I_{o2},\;\alpha_{1},\;\alpha_{2},\;R_{s},\;R_{\mathrm{sh}}\\ x_{\mathrm{DDM}-M}=I_{\mathrm{ph}},\;I_{o1},\;I_{o2},\;\alpha_{1},\;\alpha_{2},\;R_{s},\;R_{\mathrm{sh}}\\ x_{\mathrm{TDM}-C}=I_{\mathrm{ph}},\;I_{o1},\;I_{o2},I_{o3},\;\alpha_{1},\;\alpha_{2},\alpha_{3},\;R_{s},\;R_{\mathrm{sh}}\\ x_{\mathrm{TDM}-M}=I_{\mathrm{ph}},\;I_{o1},\;I_{o2},I_{o3},\;\alpha_{1},\;\alpha_{2},\alpha_{3},\;R_{s},\;R_{\mathrm{sh}} \end{array}\right. \end{array}$$

(21) $$\mathrm{RMSE}\;=\sqrt{\frac{1}{K}\sum_{k=1}^{K}f{\left(x\right)}^{2}}$$

where, *f*(*x*) is the current function expressing the difference between the estimated and experimentally measured current, *I*_e_ is the estimated current, *I*_m_ is the measured current, *f*_SDM − *C*_(*x*), *f*_SDM − *M*_(*x*), *f*_DDM − *C*_(*x*), *f*_DDM − *M*_(*x*), *f*_TDM − *C*_(*x*), and *f*_TDM − *M*_(*x*) are the current functions of SDM‐C, SDM‐M, DDM‐C, DDM‐M, TDM‐C, and TDM‐M, respectively, $I_{\mathrm{SDM}-C_{m}}$, $I_{\mathrm{SDM}-M_{m}}$, $I_{\mathrm{DDM}-C_{m}}$, $I_{\mathrm{DDM}-M_{m}}$, $I_{\mathrm{TDM}-C_{m}}$, and $I_{\mathrm{TDM}-M_{m}}$ are the measured currents of SDM‐C, SDM‐M, DDM‐C, DDM‐M, TDM‐C, and TDM‐M, respectively, *x* is the decision variable, and the RMSE is the objective function.

## 3 Weighted Leader Search Algorithm for Defined Parameter Extraction Problem

A detailed description of the WLS algorithm used to solve the PV parameter extraction optimization problem and brief summaries of the SWO, SGA, RECAA, FFA, KOA, and RIME are given in the following subsections.

### 3.1 WLS

**Figure 4:**
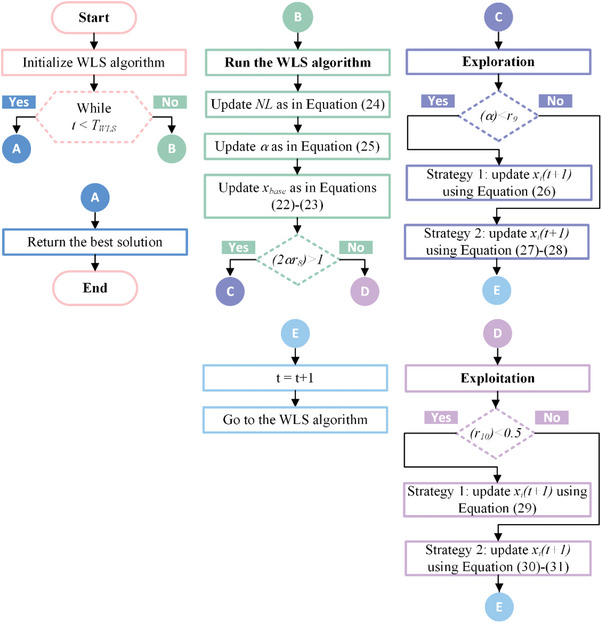
Flowchart of WLS algorithm.

The WLS algorithm was proposed by Wang et al. in 2023. The mathematical background and operating principles of this metaheuristic algorithm are presented under three subheadings: weighted‐leader mechanism and initialization phase, exploration phase, and exploitation phase. In addition, the flow diagram of the WLS algorithm is given in **Figure** **4** and the pseudocode is given in **Algorithm** **1**.

Algorithm 1 **Pseudocode of WLS algorithm.**

**Table:** 

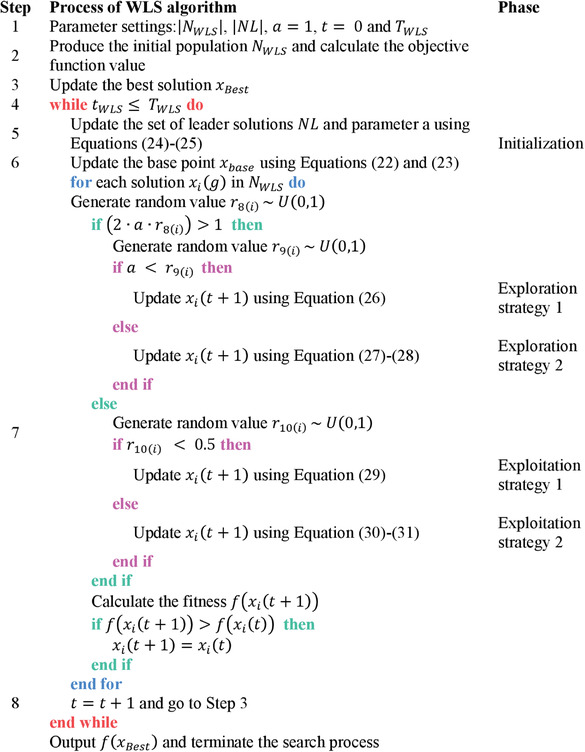

#### 3.1.1 Weighted‐Leader Mechanism and Initialization Phase

In stochastic search concept, initiation starts through the current optimal point/base point. From this point, which is randomly selected and searched, the global optimum solution is gradually converged. If the difference between the current optimal point and the local optimum is very small, early convergence may occur. To overcome this problem, the WLS algorithm proposes a weighted‐leader mechanism that uses a set of leader solutions. This mechanism aims to increase the diversity of the base point. The base point obtained from weighted maximum likelihood estimation and weight coefficient for leader solutions are given in Equations (22) and (23), set of selected leader solutions' capacity and the control parameter associated with current iteration and maximum iteration are given in Equations (24)‐ and (25), respectively.

In the WLS algorithm, the base point (*x*_base_) is calculated in five steps using Equations (22)–(25). For this, the initial population is first created at *t* = 0. Then, the initial solution *x_i_* (0), which denotes the *i^th^* solution in the *t^th^* generation, is generated. For each initial solution (*x_i_* (0), the objective function (*f*(*x*)) is evaluated. In the third stage, the results found by the population are ranked from the smallest to the largest. In the next stage, the leading solutions are studied. The first one of the sorted function values are taken as the leader solutions (|*NL*|). These solutions represent *x*_L*i*_. In the last (fifth stage), *x*_base_(0) is calculated using Equations (22)–(25).

(22) $$x_{\mathrm{base}}\left(t\right)=\begin{array}{cc} \left(1/\left|NL\right|\right)\sum_{1}^{\left|\mathrm{NL}\right|}\omega_{i}\;x_{Li}\left(t\right), & x_{Li}\left(t\right)\;\varepsilon \;NL \end{array}$$

(23) $$\omega_{i}=\ln\left(\left|\mathrm{NL}\right|+1\right)/\left[\sum_{1}^{\left|\mathrm{NL}\right|}\ln\left(\left|\mathrm{NL}\right|+1-\ln\left(i\right)\right)\right]\;$$

(24) $$\left|\mathrm{NL}\right|=\max\left\lfloor 1,\;\left[\left(\left|N_{\mathrm{WLS}}\cdot \alpha \right|\right)/K\right]\right\rfloor \;$$

(25) $$\alpha =1-t/T_{\mathrm{WLS}}$$

where, *x*_base_(*t*) is the base point in the *t* ^th^ generation, *NL* is the set of selected leader solutions, |*NL*| is the set of selected leader solutions’ capacity, *ω*_*i*_ is the weight coefficient for the *i^th^* leader solution, *x*_L*i*_(*t*) is the *i^th^* leader solution in the *t^th^* generation, *N*_WLS_ is the population of the algorithm, |*N*_WLS_| is the population size of the algorithm, *α* is the control parameter of WLS, *K* is the scale parameter of WLS with a value of 5, *t* is the current iteration, and *T*_WLS_ is the number of maximum iterations.

#### 3.1.2 Exploration Phase

The WLS algorithm uses two exploration strategies, boundary information, and Lévy distribution, and calculates *x_i_* (*t* + 1), which denotes the *i* ^th^ solution in the (*t* + 1)^th^ generation. The first exploration strategy, which is managed by Equation (26) and using the boundary information, focuses on increasing the diversity of the distribution of the base point. And with Equation (26), an exploration activity is performed around the weighted base point. The second exploration strategy is carried out with Equation (27), where the Lévy distribution is used to generate random steps. The Lévy distribution is given in Equation (28).

(26) $$x_{i}\left(t+1\right)=\begin{array}{c} x_{\mathrm{base}}\left(t\right)+r_{2\left(i\right)}\cdot \left[c_{i}\cdot x_{i}\left(t\right)-x_{\mathrm{base}}\left(t\right)\right]+2\cdot \left[r_{3\left(i\right)}-0.5\right]\cdot \left[r_{4\left(i\right)}\cdot \left(\mathrm{LB}+r_{5\left(i\right)}\cdot \left(\mathrm{UB}-\mathrm{LB}\right)\right)\right]\\ \left\{\begin{array}{c} x_{i}\left(t\right)\;\varepsilon \;N_{\mathrm{WLS}}\;\\ r_{2\left(i\right)\;}∼\;N\left(0,1\right)\\ r_{1\left(i\right)},\;r_{3\left(i\right)},\;r_{4\left(i\right)},\;r_{5\left(i\right)}\;∼\;U\left(0,1\right)\;\\ c_{i}=2\cdot r_{1\left(i\right)} \end{array}\right. \end{array}\;$$

(27) $$x_{i}\;\left(t+1\right)=\begin{array}{cc} x_{\mathrm{base}}\left(t\right)+\left[c_{i}\cdot x_{i}\left(t\right)-x_{\mathrm{base}}\left(t\right)\right]\;⊗LF\left(D\right), & x_{i}\left(t\right)\;\in N_{WLS} \end{array}$$

(28) $$LF\;\left(D\right)=\begin{array}{c} \mu /{\left|\bar{v}\right|}^{1/\beta }\\ \left\{\begin{array}{c} \mu \;∼\;N\left(0,I_{1\times D}\right)\cdot \sigma \\ \bar{v}\;∼\;N\left(0,I_{1\times D}\right)\\ \sigma ={\left[\left(\Gamma \left(1+\beta \right)\;\sin\left(\left(\pi \;\beta \right)/2\right)\right)/\left(\beta \cdot \Gamma \left(\left(1+\beta \right)/2\right)\cdot 2^{\left(\beta -1\right)/2}\right)\right]}^{1/\beta } \end{array}\right. \end{array}\;$$

where, *x_i_* (*t* + 1) is the *i^th^* solution in the (*t* + 1)^*th*^ generation, *c_i_* is the random number uniformly distributed in the range of 0 to 2, *x_i_* (*t*) is the *i^th^* solution in the *t^th^* generation, *LB* is lower bound of the defined problem, *UB* is upper bound of the defined problem, *r*_1(*i*)_, *r*_2(*i*)_, *r*_3(*i*)_, *r*_4(*i*)_, and *r*_5(*i*)_ are the random numbers uniformly distributed in the range of 0 to 1, *LF* is lévy distribution, and *D* is dimension/size of the problem.

#### 3.1.3 Exploitation Phase

The WLS algorithm uses two exploitation strategies consisting of randomly selected solutions and the solutions that are generated according to the descent direction of the objective function and calculates *x_i_* (*t* + 1), which denotes the *i^th^* solution in the (*t* + 1)^th^ generation. The first exploitation strategy using randomly selected solutions is given in Equation (29). In this equation, *x*_m_ and *x_k_* are the randomly selected solutions. The difference of these solutions is used and local exploitation is performed. The second exploitation strategy uses the unit descent direction vector given by Equation (30) and is implemented by Equations (31) and (32). In this strategy, solutions generated according to the descent direction of the objective function value are used.

(29) $$x_{i}\;\left(t+1\right)=\begin{array}{c} x_{i}\left(t\right)+\left[{\left.\alpha \cdot r_{6\left(i\right)}\cdot \left(x_{m}\left(t\right)-x_{k}\left(t\right)\right)\right|}_{m\neq k}\right]\\ \left\{\begin{array}{c} r_{6\left(i\right)}\;∼\;U\left(0,1\right)\\ x_{i}\left(t\right),x_{m}\left(t\right),x_{k}\left(t\right)\;\varepsilon \;N_{\mathrm{WLS}}\;\left(i\neq m\neq k\right) \end{array}\right. \end{array}$$

(30) $$v=\left(x_{\mathrm{base}}\left(t\right)-x_{i}\left(t\right)\right)/\mathrm{abs}\left(x_{\mathrm{base}}\left(t\right)-x_{i}\left(t\right)\right)\;$$

(31) $$x_{i}\;\left(t+1\right)=\begin{array}{c} x_{i}\left(t\right)+\alpha \cdot r_{7\left(i\right)}\cdot v\cdot \mathrm{abs}\left(c_{i}\cdot x_{i}\left(t\right)-x_{\mathrm{base}}\left(t\right)\right)\\ \left\{\begin{array}{c} r_{7\left(i\right)\;}∼\;U\left(0,1\right)\\ x_{i}\left(t\right)\neq x_{\mathrm{base}}\left(t\right)\\ x_{i}\left(t\right)\;\varepsilon \;N_{\mathrm{WLS}} \end{array}\right. \end{array}$$

(32) $$x_{i}\;\left(t+1\right)=\begin{array}{c} x_{i}\left(t\right)+\alpha \cdot r_{7\left(i\right)}\cdot \mathrm{ab}s\left(c_{i}\cdot x_{i}\left(t\right)-x_{\mathrm{base}}\left(t\right)\right)\\ \left\{\begin{array}{c} r_{7\left(i\right)\;}∼\;U\left(0,1\right)\\ x_{i}\left(t\right)=x_{\mathrm{base}}\left(t\right)\\ x_{i}\left(t\right)\;\varepsilon \;N_{\mathrm{WLS}} \end{array}\right. \end{array}$$

where, *r*_6(*i*)_ is the random number uniformly distributed in the range of 0 to 1, *x*_m_(*t*) and *x*_k_(*t*) are the *i* ^th^ random solutions in the *t^th^* generation, *v* is the unit descent direction of the objective function value, and *r*_7(*i*)_ is the random number uniformly distributed in the range of 0 to 1.^[^ ^53^ ^]^

### 3.2 Brief Summary of SWO, SGA, RECAA, FFA, KOA, and RIME Algorithms

In addition to the detailed description of the WLS algorithm, SWO, SGA, RECAA, FFA, KOA, and RIME algorithms are also used in this study. The sources of inspiration for these metaheuristic algorithms, the motivation for their development, and brief summaries of the control parameters that affect the performance of the algorithms are given in the subsections and are also presented in **Table** **2**.

**Table 2:** Brief summary of WLS, SWO, SGA, RECAA, FFA, KOA, and RIME algorithms.

Algorithm	Inspiration	Motivation	Control Parameter	Value
WLS	Maximum likelihood estimation and set of leader solutions in the population	It acts on leader solutions in the population and uses a weighted‐leader mechanism.	*α*	[1,0]
SWO	Female spider wasp	Mimics the searching, following and escaping, nesting, and mating behaviors of female spider wasps.	*TR* *CR* $N_{\mathrm{SW}O_{m}}$	0.3 0.2 20
SGA	Shrimp and goby fish	Shrimps and goby fishes are inspired by the mutual partnership relationship of shelter and safety for mutual benefit.	*α*_SGA_	[2,1]
RECAA	Reversible cellular automata	Reversible cellular automata are inspired by their dynamic behavior.	*Prop* *Lower*_r_ *Upper*_r_ *n*_el_	1.7 2 6 2
FFA	Fennec fox	Fennec foxes are inspired by their digging and escaping from predator attacks.	*N*_FFA_ *T*_FFA_	50 10000
KOA	Kepler's laws of planetary motion	Inspired by the Kepler's laws of planetary motion.	*N*_KOA_ ${\bar{T}}_{\mathrm{KOA}}$ *μ*_0_ *γ*	50 3 0.1 15
RIME	Rime‐ice	Inspired by the growth mechanism of rime‐ice, which comes in two different forms: soft‐rime and hard‐rime.	*w*	5

#### 3.2.1 SWO

The SWO algorithm was developed in 2023 by Abdel‐Basset et al. inspired by female spider wasps. In this meta‐heuristic algorithm, the searching, following and escaping, nesting, and mating behaviors of female spider wasps are emulated. With searching behavior, it focuses on exploration, with following and escaping behavior, it manages the balance between exploration and exploitation, and finally with nesting behavior, it focuses on exploitation. The SWO has three control parameters, namely, the *TR* tradeoff rate, the *CR* crossover probability, and the $N_{\mathrm{SW}O_{m}}$ minimum population size. The values given in the original article,^[^ ^54^ ^]^ 0.3 for *TR*, 0.2 for *CR*, and 20 for $N_{\mathrm{SW}O_{m}}$ are used.

#### 3.2.2 SGA

The SGA algorithm was developed in 2023 by Sang‐To et al. and inspired by the relationship between shrimps and goby fishes. Goby fishes use the burrows dug by shrimps as a shelter/resting place. Shrimps are almost blind. Therefore, shrimps use goby fishes as an alarm system to be aware of predators. This metaheuristic algorithm mimics this shelter and security relationship between shrimps and goby fishes. The SGA algorithm is modeled in two stages that consist of a signal transmission and alarm system against predators and the shelter and inside activity. The first phase focuses on exploration and the second on exploitation. The SGA has only one control parameter, denoted as *α*_*SGA*_. The study uses the value that ranging from 2 to 1 as suggested in the original article.^[^ ^55^ ^]^

#### 3.2.3 RECAA

The RECAA algorithm was developed in 2022 by Seck‐Tuoh‐Mora et al. and inspired by reversible cellular automata. This metaheuristic algorithm mimics the reversible behavior of elementary cellular automata. The RECAA algorithm operates with three rules: rules for exploration using two smart‐cells, exploitation using the information of a smart‐cell, and rounding values in a smart‐cell. The first rule focuses on exploration and the second on exploitation. The RECAA has four control parameters including *prop* proportion, *lower*_r_ lower rounding, *upper*_r_ upper rounding, *n*_el_ number of elitist solutions. *prop*, *lower*_*r*_, *uppe* *r*_*r*_, and *n*_el_ are taken as 1.7, 2, 6, and 2, respectively, which are the values given in the original article.^[^ ^56^ ^]^

#### 3.2.4 FFA

The FFA algorithm was developed in 2022 by Trojovska et al. and inspired by the fennec foxes. This metaheuristic algorithm mimics the digging and escaping from the predator attacks of fennec foxes. The FFA algorithm is executed in two phases: digging under the sand to find prey and escaping predator attacks. The first phase focuses on exploitation and the second on exploration. The FFA has two control parameters, *N*_FFA_ number of fennec foxes and *T*_FFA_ total number of iterations.^[^ ^57^ ^]^ *N*_FFA_ and *T*_FFA_ are taken as 50 and 10000, respectively.

#### 3.2.5 KOA

The KOA algorithm was developed in 2023 by Abdel‐Basset et al. and inspired by Kepler's laws of planetary motion. In this metaheuristic algorithm, the sun and the planets orbiting the sun in an elliptical orbit are imitated. The KOA algorithm is executed in seven steps including initialization, defining the gravitational force, calculating an object' velocity, escaping from the local optimum, updating objects' positions, updating distance with the sun, and elitism. It focuses on exploration and exploitation in the steps of updating objects' positions and updating distance with the sun. The KOA has three control parameters including ${\bar{T}}_{\mathrm{KOA}}$ the number of cycles, *μ*_0_ constant number, and *γ* an initial value. ${\bar{T}}_{\mathrm{KOA}}$, *μ*_0_, and *γ* are taken as 3, 0.1, and 15, respectively, these are the values provided in the original article.^[^ ^58^ ^]^

#### 3.2.6 RIME

The RIME algorithm was developed in 2023 by Su et al. and inspired by the rime‐ice. This metaheuristic algorithm mimics the growth mechanism of rime‐ice, which takes two distinct forms: soft‐rime and hard‐rime. The RIME algorithm is executed in four steps: the initialization of rime clusters, the soft‐rime strategy, the hard‐rime mechanism, and the greedy selection mechanism. The RIME has one control parameter *w*, which is a constant number. In this study, *w* is taken as 5, the value given in the original article.^[^ ^59^ ^]^

## 4 Results and Evaluation of Defined PV Parameter Extraction Problem

The section presents the results, evaluation, and sensitivity analysis of the algorithms after they were run for 10 000 iterations and 30 runs. The results of this optimization are presented in the following paragraphs under nine subsections consisting of specifications of the computer, selection of PV cells and modules, definition of boundaries of decision variables, definition of control parameters of algorithms, results of PV parameter extraction, evaluation metrics‐based assessment, statistical tests‐based assessment, convergence curves, and sensitivity analysis.

### 4.1 Specifications of the Computer

All of the algorithms have been coded as M‐files in MATLAB software, and the computer used to run them has an Intel(R) Core (TM) i7‐4790 CPU@3.60GHz RAM 24GB.

### 4.2 Selection of PV Cells and Modules

For the PV parameter extraction problem, 2 PV cells and 6 PV modules were selected, and the required information about these devices is given in **Table** **3**. It is worth noting that, in this study, the experimentally measured data (current and voltage) of PV cells consisting of R.T.C France^[^ ^60^ ^]^ and PVM 752,^[^ ^61^ ^]^ and the PV modules consisting of Schutten Solar STM6‐40/36,^[^ ^62^ ^]^ Leibold Solar Module (LSM 20),^[^ ^61^ ^]^ Photowatt‐PWP 201,^[^ ^60^ ^]^ Schutten Solar STP6‐120/36,^[^ ^62^ ^]^ Kyocera KC200GT 215,^[^ ^63^ ^]^ and ESP‐160 PPW^[^ ^64^ ^]^ are used. These data were obtained through experimental studies and can be accessed directly from the given references.

**Table 3:** Information of PV cells and modules.

PV model			PV brand	PV type	Number of series‐connected PV cells	PV test conditions	
SDM‐C1	and	DDM‐C1	R.T.C France	Silicon	*N_s_* = 1	*G* = 1000 W/m^2^	*T* = 33 °C
SDM‐C2	and	DDM‐C2	PVM 752	Thin film	*N_s_* = 1	*G* = 1000 W/m^2^	*T* = 25 °C
SDM‐M1	and	DDM‐M1	Schutten Solar STM6‐40/36	Mono‐crystalline	*N_s_* = 36	*G* = 1000 W/m^2^	*T* = 51 °C
SDM‐M2	and	DDM‐M2	Leibold Solar Module (LSM 20)	Mono‐crystalline	*N_s_* = 20	*G* = 360 W/m^2^	*T* = 24 °C
SDM‐M3	and	DDM‐M3	Photowatt‐PWP 201	Poly‐crystalline	*N_s_* = 36	*G* = 1000 W/m^2^	*T* = 45 °C
SDM‐M4	and	DDM‐M4	Schutten Solar STP6‐120/36	Poly‐crystalline	*N_s_* = 36	*G* = 1000 W/m^2^	*T* = 55 °C
SDM‐M5	and	DDM‐M5	Kyocera KC200GT 215	Multi‐crystalline	*N_s_* = 54	*G* = 1000 W/m^2^	*T* = 25 °C
SDM‐M6	and	DDM‐M6	ESP‐160 PPW	Poly‐crystalline	*N_s_* = 72	N/A	*T* = 45 °C

### 4.3 Definition of Boundaries of Decision Variables

As a result of solving the problem, there are 5 parameters to be estimated as *I*_ph_, *I_o_*, *α*, *R*_s_, and *R*_sh_ for SDM, 7 parameters to be estimated as *I*_ph_, *I*_o1_, *I*_o2_, *α*_1_, *α*_2_, *R*_s_, and *R*_sh_ for DDM, and 9 parameters to be estimated as *I*_ph_, *I*_*o*1_, *I*_o2_, *I*_o3_, *α*_1_, *α*_2_, *α*_3_, *R*_s_, and *R_sh_* for TDM. The lower and upper bounds of the decision variables of SDM, DDM, and TDM are given in **Tables** 4, 5, 6, respectively.

**Table 4:** Boundaries of decision variables for SDM.

PV model	Lower bound					Upper bound				
	*I*_ph_ [A]	*I*_o_ [µA]	*α* (‐)	*R_s_* (Ω)	*R_sh_* (Ω)	*I_ph_* (A)	*I_o_* (µA)	*α* (‐)	*R_s_* (Ω)	*R_sh_* (Ω)
SDM‐C1	0	0	1	0	0	1	10	2	1	1000
SDM‐C2	0	0	1	0	0	1	10	2	1	1000
SDM‐M1	0	0	1	0	0	2	50	60	0.36	1000
SDM‐M2	0	0	1	0	0	1	10	2	10	5000
SDM‐M3	0	0	1	0	0	2	50	50	2	2000
SDM‐M4	0	0	1	0	0	8	50	50	0.36	1500
SDM‐M5	0	0	1	0	0	10	10	128	0.5	500
SDM‐M6	0	0	1	0	0	8	10000	3	0.55	1000

**Table 5:** Boundaries of decision variables for DDM.

PV model	Lower bound							Upper bound						
	*I*_ph_ [A]	*I*_o1_ [µA]	*I*_o2_ [µA]	*α*_1_ (‐)	*α*_2_ (‐)	*R*_s_ [Ω]	*R*_sh_ [Ω]	*I*_ph_ [A]	*I*_o1_ [µA]	*I*_o2_ [µA]	*α*_1_ (‐)	*α*_2_ (‐)	*R*_s_ [Ω]	*R*_sh_ [Ω]
DDM‐C1	0	0	0	1	1	0	0	1	10	10	2	2	1	1000
DDM‐C2	0	0	0	1	1	0	0	1	10	10	2	2	1	1000
DDM‐M1	0	0	0	1	1	0	0	2	50	50	60	60	0.36	1000
DDM‐M2	0	0	0	1	1	0	0	1	10	10	2	2	10	5000
DDM‐M3	0	0	0	1	1	0	0	2	50	50	50	50	2	2000
DDM‐M4	0	0	0	1	1	0	0	8	50	50	50	50	0.36	1500
DDM‐M5	0	0	0	1	1	0	0	10	10	10	128	128	0.5	500
DDM‐M6	0	0	0	1	1	0	0	8	10000	10000	3	3	0.55	1000

**Table 6:** Boundaries of decision variables for TDM.

PV model	Lower bound									Upper bound								
	*I*_ph_ [A]	*I*_o1_ [µA]	*I*_o2_ [µA]	*I*_o3_ [µA]	*α*_1_ (‐)	*α*_2_ (‐)	*α*_3_ (‐)	*R*_s_ [Ω]	*R*_sh_ [Ω]	*I*_ph_ [A]	*I*_*o*1_ [µA]	*I*_*o*2_ [µA]	*I*_*o*3_ [µA]	*α*_1_ (‐)	*α*_2_ (‐)	*α*_3_ (‐)	*R*_s_ [Ω]	*R*_sh_ [Ω]
TDM‐C1	0	0	0	0	1	1	1	0	0	1	10	10	10	2	2	2	1	1000
TDM‐C2	0	0	0	0	1	1	1	0	0	1	10	10	10	2	2	2	1	1000
TDM‐M1	0	0	0	0	1	1	1	0	0	2	50	50	50	60	60	60	0.36	1000
TDM‐M2	0	0	0	0	1	1	1	0	0	1	10	10	10	2	2	2	10	5000
TDM‐M3	0	0	0	0	1	1	1	0	0	2	50	50	50	50	50	50	2	2000
TDM‐M4	0	0	0	0	1	1	1	0	0	8	50	50	50	50	50	50	0.36	1500
TDM‐M5	0	0	0	0	1	1	1	0	0	10	10	10	10	128	128	128	0.5	500
TDM‐M6	0	0	0	0	1	1	1	0	0	8	10000	10000	10000	3	3	3	0.55	1000

### 4.4 Definition of Control Parameters of Algorithms

The studied algorithms, WLS, SWO, SGA, RECAA, FFA, KOA, and RIME, have 1, 3, 1, 4, 2, 4, and 1 control parameters, respectively, and these parameters and their values are given in Section 3.2 also in Table 2.

### 4.5 Results of PV Parameter Extraction

Optimization results of the studied algorithms are presented in six PV model categories, namely SDM‐C, DDM‐C, TDM‐C, SDM‐M, DDM‐M, and TDM‐M. The discussions are presented in the following paragraphs for each model category.

#### 4.5.1 SDM‐C

**Figure 5:**
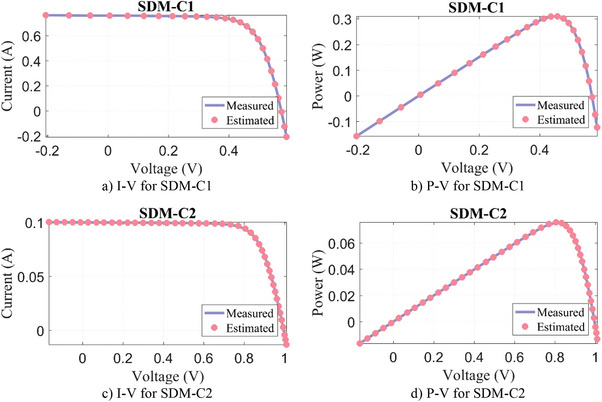
Experimental measurement data versus estimated by WLS algorithm for SDM‐C.

In this article, two PV cells, Silicon R.T.C France and Thin film PVM 752, are studied. The parameter extraction results of WLS, SWO, SGA, RECAA, FFA, KOA, and RIME algorithms for SDM‐C1 and SDM‐C2 are given in **Table** **7**. Table 7 presents the results of the objective function/RMSE and the estimations made for the five parameters (*I*_ph_, *I*_o_, *α*, *R*_s_, and *R*_sh_) after successfully solving the problem for SDM. In these two PV models, the WLS algorithm has achieved the most successful result compared to other algorithms with the lowest RMSE value. In addition, **Figure** **5** compares two data, the experimentally measured current and voltage data, and the results estimated by the WLS algorithm using these real measurements. It is seen that the measured and the estimated data are successfully matching each other.

**Table 7:** PV parameter extraction results for SDM‐C1 and SDM‐C2.

PV model	Algorithm	*I*_ph_ [A]	*I_o_* [µA]	*R*_sh_ [Ω]	*R*_s_ [Ω]	*α*	RMSE	RMSE rank
SDM‐C1	**WLS**	0.7607755	0.3230208	53.7185247	0.0363771	1.4811855	**9.8602188E‐04** **0.00098602187789154100**	**1**
	SWO	0.7598549	2.2617735	664.2370291	0.0271573	1.7073372	4.0784489E‐03 0.00407844894279840000	4
	SGA	0.7597399	0.9261941	183.1142021	0.0320091	1.5951898	2.3128464E‐03 0.00231284637418911000	2
	RECAA	0.7650000	1.5800000	63.8460000	0.0290000	1.6610000	4.2735430E‐03 0.00427354300487877000	5
	FFA	0.7584934	0.6566938	1000.0000000	0.0340455	1.5555040	2.3556140E‐03 0.00235561396667319000	3
	KOA	0.7609740	4.3993000	580.2597903	0.0225304	1.8017992	5.7057256E‐03 0.00570572557210128000	6
	RIME	0.7624483	5.9605910	1000.0000000	0.0210906	1.8479554	6.3463249E‐03 0.00634632487146614000	7
SDM‐C2	**WLS**	0.1000248	0.0000063	682.3940530	0.6485687	1.6510944	**2.3670455E‐04** **0.00023670455391884000**	**1**
	SWO	0.1000449	0.0001554	975.1353153	0.5541752	1.9114751	5.6515823E‐04 0.00056515822938108100	4
	SGA	0.0999858	0.0000128	808.9689125	0.6314877	1.7018869	2.7457722E‐04 0.00027457721820209300	2
	RECAA	0.1000000	0.0000420	800.2800000	0.5940000	1.7958500	4.0877471E‐04 0.00040877470956110500	3
	FFA	0.1137620	0.0000000	14.5885596	0.0000000	1.3045975	2.5399581E‐02 0.02539958065712840000	6
	KOA	0.1137862	0.0000000	14.5802186	0.0000000	1.3501296	2.5399585E‐02 0.02539958507883550000	7
	RIME	0.1001064	0.0002496	1000.0000000	0.5383395	1.9571050	6.3311214E‐04 0.00063311213905694900	5

#### 4.5.2 DDM‐C

**Figure 6:**
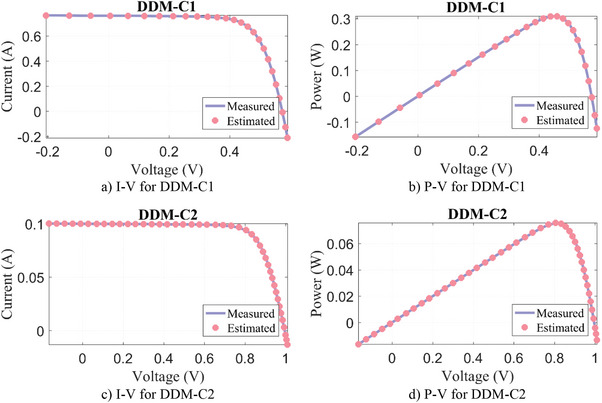
Experimental measurement data versus estimated by WLS algorithm for DDM‐C.

The parameter extraction results of our algorithms for DDM‐C1 and DDM‐C2 are given in **Table** **8**. It shows the results of the parameter estimations for the seven variables (*I*_ph_, *I*_o1_, *I*_o2_, *α*_1_, *α*_2_, *R*_s_, and *R*_sh_) and the objective function/RMSE. In DDM‐C1 and DDM‐C2 PV models, the WLS algorithm has again achieved the most successful result compared to other algorithms with the lowest RMSE value. Moreover, the results estimated by the WLS against the experimentally measured data are given in **Figure** **6**. It is seen that the measured and estimated data are overlapping successfully.

**Table 8:** PV parameter extraction results for DDM‐C1 and DDM‐C2.

PV model	Algorithm	*I*_ph_ [A]	*I*_*o*1_ [µA]	*I*_*o*2_ [µA]	*R*_sh_ [Ω]	*R*_s_ [Ω]	*α*_1_	*α*_2_	RMSE	RMSE rank
DDM‐C1	**WLS**	0.7596539	0.2198994	0.3837244	82.6857733	0.0342132	1.6704112	1.5130274	**1.5026880E‐03** **0.00150268797016254000**	**1**
	SWO	0.7599772	0.0037201	1.5600970	586.4555569	0.0293828	1.9801155	1.6585517	3.2613084E‐03 0.00326130840998663000	5
	SGA	0.7601164	0.0000202	1.1809596	181.9483519	0.0308777	1.1179704	1.6251292	2.7029722E‐03 0.00270297216920708000	3
	RECAA	0.7600000	0.9800000	1.6758400	903.6006614	0.0269000	1.6500942	1.8271916	4.7350399E‐03 0.00473503990556186000	6
	FFA	0.7577715	0.0000000	0.6204797	999.9953492	0.0341646	1.9999907	1.5493278	2.3676813E‐03 0.00236768132557370000	2
	KOA	0.7642362	3.1184357	3.3567719	495.6636138	0.0196128	1.9289750	1.8179702	7.0622957E‐03 0.00706229568833485000	7
	RIME	0.7598715	2.9996914	0.1082427	933.9510296	0.0308623	1.8491492	1.4408732	3.2607376E‐03 0.00326073760070069000	4
DDM‐C2	**WLS**	0.0999992	0.0000148	0.0000001	828.8228193	0.6277444	1.7131459	1.9434341	**2.8637646E‐04** **0.00028637646262133700**	**1**
	SWO	0.1000836	0.0000315	0.0002265	996.3091047	0.5368612	1.9447721	1.9628304	6.3854789E‐04 0.00063854788857137400	6
	SGA	0.0999465	0.0000520	0.0000000	998.6946261	0.5901763	1.8135946	1.0468672	4.1445118E‐04 0.00041445118468021500	2
	RECAA	0.1001000	0.0000000	0.0000700	856.3630000	0.5863800	1.5794000	1.8394000	4.7805541E‐04 0.00047805540581174700	3
	FFA	0.1137620	0.0000000	0.0000000	14.5885596	0.0000000	2.0000000	1.0000000	2.5399581E‐02 0.02539958065712840000	7
	KOA	0.0993257	0.0000022	0.0000000	921.6061158	0.6839679	1.5807053	1.6735777	5.6962345E‐04 0.00056962345385616000	4
	RIME	0.1000971	0.0002128	0.0000000	1000.0000000	0.5442074	1.9414377	1.2860988	6.0876143E‐04 0.00060876143360495600	5

#### 4.5.3 TDM‐C

**Figure 7:**
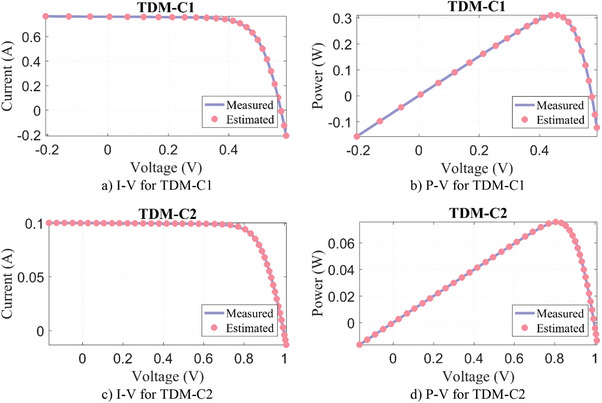
Experimental measurement data versus estimated by WLS algorithm for TDM‐C.

The parameter extraction results of our algorithms for TDM‐C1 and TDM‐C2 are given in **Table** **9**. This table shows the results of estimations for nine parameter values (*I*_ph_, *I*_o1_, *I*_o2_, *I*_o3_, *α*_1_, *α*_2_, *α*_3_, *R*_s_, and *R*_sh_) and the objective function/RMSE value. In TDM‐C1 and TDM‐C2 PV models, the WLS algorithm once more has achieved the most successful result compared to other algorithms with the lowest RMSE result. The results estimated by WLS against the experimental measurement data are given in **Figure** **7**. It can be seen that the measured and estimated data are compatible to each other.

**Table 9:** PV parameter extraction results for TDM‐C1 and TDM‐C2.

PV model	Algorithm	*I*_ph_ [A]	*I*_o1_ [µA]	*I*_*o*2_ [µA]	*I*_*o*3_ [µA]	*R*_sh_ [Ω]	*R*_s_ [Ω]	*α*_1_	*α*_2_	*α*_3_	RMSE	RMSE rank
TDM‐C1	**WLS**	0.7607736	0.0065272	0.3227564	0.0075301	53.9336692	0.0363543	1.8235016	1.4812836	1.9990132	**9.8607655E‐04** **0.00098607655456071300**	**1**
	SWO	0.7604827	0.6686398	3.6509133	0.0118871	967.2318487	0.0280848	1.5912321	1.9999617	1.7421566	3.9776329E‐03 0.00397763288442748000	6
	SGA	0.7597696	1.6249394	0.0325579	0.0003401	182.6941257	0.0334629	1.7351446	1.3528741	1.2659691	2.2354564E‐03 0.00223545641730287000	2
	RECAA	0.7600000	0.3094220	1.0400000	0.3700000	616.2998210	0.0300000	1.8719600	1.6300000	1.7540000	3.2905871E‐03 0.00329058712094085000	5
	FFA	0.7571118	0.0000000	0.0000000	0.9477693	999.9941444	0.0315379	1.8403061	1.9998272	1.5979831	2.9401338E‐03 0.00294013380945741000	4
	KOA	0.7666875	1.4800522	2.6243417	1.0646784	370.4524151	0.0238513	1.8012514	1.8104408	1.9382857	7.0757165E‐03 0.00707571650627115000	7
	RIME	0.7597031	2.9731764	0.5157347	0.0676712	831.6171573	0.0333288	2.0000000	1.6846853	1.3926628	2.5730988E‐03 0.00257309884228817000	3
TDM‐C2	**WLS**	0.0999351	0.0000000	0.0000138	0.0000002	922.2758749	0.6296543	1.8507960	1.7076934	1.9524188	**2.8178337E‐04** **0.00028178337212571100**	**1**
	SWO	0.1001247	0.0002840	0.0000050	0.0000326	999.0060417	0.5297551	1.9945333	1.8055957	1.9730290	6.6306655E‐04 0.00066306654546055600	5
	SGA	0.1000423	0.0000000	0.0001091	0.0000000	997.3063544	0.5674947	1.4085096	1.8786647	1.2054420	5.1121953E‐04 0.00051121953369510300	3
	RECAA	0.1000000	0.0001700	0.0000000	0.0000000	989.1000000	0.5500000	1.9200000	1.7843000	1.2189000	5.8025147E‐04 0.00058025147493018900	4
	FFA	0.1137620	0.0000000	0.0000000	0.0000000	14.5885596	0.0000000	1.0000000	2.0000000	1.3710891	2.5399581E‐02 0.02539958065712840000	6
	KOA	0.1136534	0.0000000	0.0000000	0.0000000	14.6091291	0.0000000	1.3767267	1.6492602	1.1816597	2.5399663E‐02 0.02539966296711100000	7
	RIME	0.1000063	0.0000685	0.0000000	0.0000000	999.8565447	0.5827386	1.8371573	1.1586014	1.2778124	4.4762247E‐04 0.00044762247174180300	2

#### 4.5.4 SDM‐M

In this paper, six PV modules consisting of mono‐crystalline Schutten Solar STM6‐40/36, mono‐crystalline Leibold Solar Module (LSM 20), poly‐crystalline Photowatt‐PWP 201, poly‐crystalline Schutten Solar STP6‐120/36, multi‐crystalline Kyocera KC200GT 215, and poly‐crystalline ESP‐160 PPW are studied. The parameter extraction results of WLS, SWO, SGA, RECAA, FFA, KOA, and RIME algorithms for SDM‐M1‐M6 are given in **Table** **10**, where five parameters estimated for SDM and the objective function/RMSE results are tabulated. In SDM‐M1‐M5 PV models, the WLS algorithm obtained the most successful result compared to other algorithms with the lowest RMSE result. On the other hand, in SDM‐M6, the most successful result is obtained with the FFA algorithm. However, when the result calculated with the WLS algorithm is compared with the FFA, it is seen that there is a difference of 3.85E‐13%, which is negligibly small. In addition, the actual current and voltage data and the estimated ones by the WLS algorithm are given in **Figure** **8**. When the results in Figure 8 are analyzed, it is seen that the matching between the measured and estimated data is quite successful.

**Table 10:** PV parameter extraction results for SDM‐M1‐M6.

PV model	Algorithm	*I*_ph_ [A]	*I_o_* [µA]	*R*_sh_ [Ω]	*R*_s_ [Ω]	*α*	RMSE	RMSE Rank
SDM‐M1	**WLS**	1.6639048	1.7386570	15.9282946	0.0042738	1.5203049	**1.7298137E‐03** **0.00172981370994069000**	**1**
	SWO	1.6604568	7.5837919	44.5194969	0.0000020	1.7011763	4.7222977E‐03 0.00472229774430756000	5
	SGA	1.6611333	5.4298727	25.0050285	0.0002257	1.6566782	3.2383131E‐03 0.00323831307777973000	4
	RECAA	1.6626000	2.2000000	19.5100000	0.0040000	1.5470000	2.8632104E‐03 0.00286321042018527000	3
	FFA	1.6650802	1.8504952	15.0785548	0.0039895	1.5272926	1.8359290E‐03 0.00183592896888397000	2
	KOA	1.6734587	23.9484166	677.7815163	0.0000000	1.8755071	2.0197226E‐02 0.02019722635492900000	6
	RIME	1.6791291	39.3280177	1000.0000000	0.0000000	1.9659490	2.8817340E‐02 0.02881733997494010000	7
SDM‐M2	**WLS**	0.1534531	0.0363567	489.8610871	0.2745738	1.4889284	**1.7982385E‐03** **0.00179823850069240000**	**1**
	SWO	0.1537348	0.4880459	2400.9305542	0.2010657	1.7930656	2.0298831E‐03 0.00202988307779873000	6
	SGA	0.1535920	0.3224992	4643.1546384	0.2142812	1.7362333	1.9690243E‐03 0.00196902427878768000	4
	RECAA	0.1530000	0.1000000	2951.3949299	0.2422000	1.5940000	1.8913763E‐03 0.00189137630302373000	3
	FFA	0.1532778	0.0742973	4996.5052030	0.2613227	1.5614823	1.8374668E‐03 0.00183746679559971000	2
	KOA	0.1537541	1.0171935	4814.2450780	0.1687343	1.9028761	2.1471621E‐03 0.00214716207716184000	7
	RIME	0.1536257	0.3295340	5000.0000000	0.2142774	1.7390753	1.9714252E‐03 0.00197142522725712000	5
SDM‐M3	**WLS**	1.0322098	3.3932327	23.5774096	0.0334139	1.3485179	**2.3860126E‐03** **0.00238601264329261000**	**1**
	SWO	1.0312977	24.8494543	620.4349889	0.0259595	1.5998880	6.9435931E‐03 0.00694359305566732000	5
	SGA	1.0342300	2.0028472	16.6132099	0.0348788	1.2948587	2.8209323E‐03 0.00282093232035669000	2
	RECAA	1.0279900	7.0200000	83.5258540	0.0311100	1.4300000	3.3050286E‐03 0.00330502864581989000	3
	FFA	1.0260521	6.2778266	131.1222381	0.0317950	1.4167146	3.3450319E‐03 0.00334503193923105000	4
	KOA	1.0420931	25.5001356	129.0360792	0.0251215	1.6004051	1.3504989E‐02 0.01350498889909180000	7
	RIME	1.0344387	50.0000000	2000.0000000	0.0222927	1.7122951	9.7905120E‐03 0.00979051198557911000	6
SDM‐M4	**WLS**	7.4725299	2.3349950	22.2199026	0.0045946	1.2756548	**1.6600603E‐02** **0.01660060312508540000**	**1**
	SWO	7.5003756	16.4662024	540.2262356	0.0034680	1.4661303	3.6821042E‐02 0.03682104241883530000	5
	SGA	7.4981861	13.0167859	1073.9968697	0.0036230	1.4399265	3.2581180E‐02 0.03258117964383290000	4
	RECAA	7.4800000	4.0150000	42.0260000	0.0043000	1.3230000	1.9203703E‐02 0.01920370319416280000	3
	FFA	7.4630067	3.3504072	1499.9764026	0.0044202	1.3069827	1.7406803E‐02 0.01740680336440250000	2
	KOA	7.5110773	44.8169436	1088.4307804	0.0027335	1.5846742	7.4332058E‐02 0.07433205838037850000	7
	RIME	7.5395041	49.7231363	1500.0000000	0.0026985	1.6008421	5.5252987E‐02 0.05525298675534460000	6
SDM‐M5	**WLS**	8.1604540	0.0142900	3.3438535	0.0039640	1.1843522	**4.4418783E‐02** **0.04441878267792130000**	**1**
	SWO	8.1100921	4.7691641	252.0183774	0.0012964	1.6592302	9.7360370E‐02 0.09736037015137990000	5
	SGA	8.1290140	0.2603373	7.1325488	0.0028992	1.3820795	6.9396311E‐02 0.06939631079515020000	4
	RECAA	8.0800000	0.0480000	21.4488000	0.0037400	1.2600000	6.2901100E‐02 0.06290109997546620000	3
	FFA	8.0663002	0.0241355	499.8700403	0.0039309	1.2154682	6.1087081E‐02 0.06108708095904500000	2
	KOA	8.1336755	9.1766720	500.0000000	0.0000000	1.7364469	1.3856416E‐01 0.13856415817707900000	7
	RIME	8.1167132	9.6971210	500.0000000	0.0008333	1.7451966	1.0480408E‐01 0.10480408290581000000	6
SDM‐M6	WLS	5.4877624	162.6430249	999.9999999	0.0005993	1.0000000	7.5783327E‐02 0.07578332684230280000	2
	SWO	5.4942921	313.8816099	290.8844414	0.0000867	1.0668874	7.7003116E‐02 0.07700311604628350000	6
	SGA	5.4933952	307.1597344	856.6149872	0.0000864	1.0645005	7.6960008E‐02 0.07696000764722620000	5
	RECAA	5.5000000	197.9690000	702.4279185	0.0005000	1.0190000	7.6230378E‐02 0.07623037779585930000	4
	**FFA**	5.4877624	162.6430253	1000.0000000	0.0005993	1.0000000	**7.5783327E‐02** **0.07578332684230250000**	**1**
	KOA	5.5038948	401.7938465	769.9464363	0.0000332	1.0947961	7.9346513E‐02 0.07934651303131220000	7
	RIME	5.4876475	162.6427352	1000.0000000	0.0005989	1.0000000	7.5783367E‐02 0.07578336660198460000	3

Figure 8Experimental measurement data versus estimated by WLS algorithm for SDM‐M.

**Figure:**
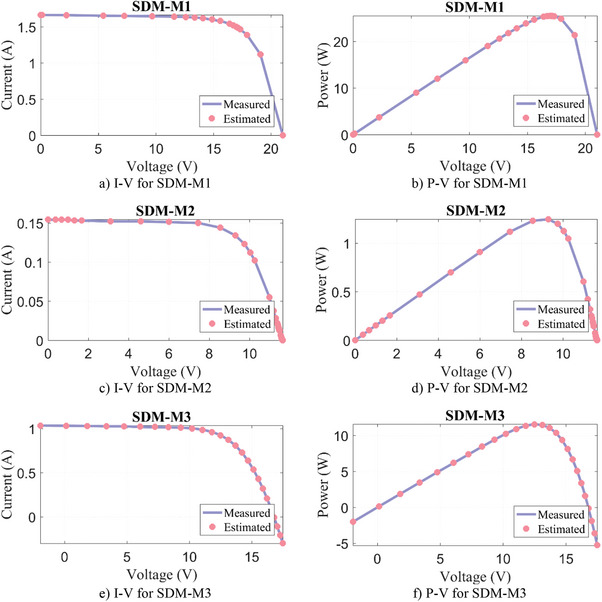

**Figure:**
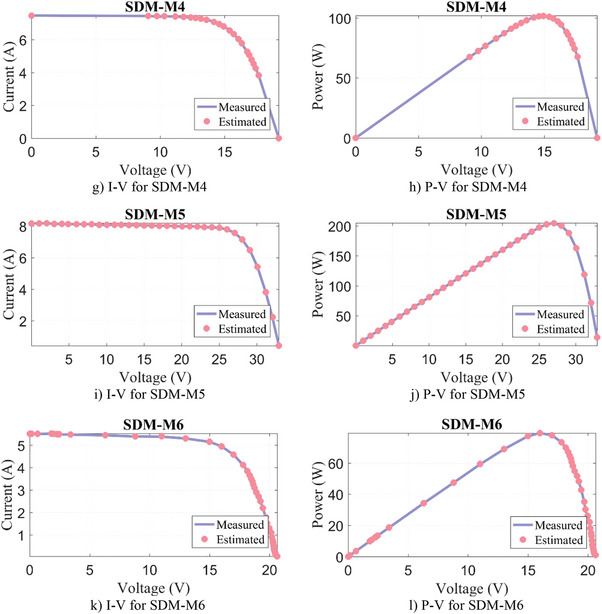

#### 4.5.5 DDM‐M

Similarly, the parameter extraction results for DDM‐M1‐M6 are given in **Table** **11**, where the estimated parameters for seven variables and the objective function/RMSE results for DDM are presented. Except for DDM‐M6, the lowest RMSE result for DDM‐M1‐M5 PV models was produced by the WLS algorithm. In DDM‐M6, the FFA algorithm calculated the most successful result again with a negligibly small difference of 3.11E‐06%. Moreover, it can be seen from **Figure** **9** that a very good match is obtained between the results estimated by the WLS and the measured data.

**Table 11:** PV parameter extraction results for DDM‐M1‐M6.

PV model	Algorithm	*I*_ph_ [A]	*I*_*o*1_ [µA]	*I*_*o*2_ [µA]	*R*_sh_ [Ω]	*R*_s_ [Ω]	*α*_1_	*α*_2_	RMSE	RMSE rank
DDM‐M1	**WLS**	1.6637173	1.3604914	12.3714146	16.7081202	0.0045733	1.4956496	2.6651905	**1.7172727E‐03** **0.00171727274314260000**	**1**
	SWO	1.6600667	6.5198206	44.5386683	34.0457735	0.0001461	1.6808932	45.9694163	3.8633634E‐03 0.00386336342888082000	6
	SGA	1.6611214	0.0432482	5.2432939	24.8507047	0.0003414	1.6435620	1.6532811	3.1814780E‐03 0.00318147797847068000	4
	RECAA	1.6620000	1.9000000	0.3000000	19.3000000	0.0046000	1.5300000	59.1000000	2.4437331E‐03 0.00244373311775140000	2
	FFA	1.6616335	0.0000000	4.5469038	20.2742419	0.0003412	60.0000000	1.6340082	3.3499608E‐03 0.00334996083470441000	5
	KOA	1.6613572	3.3821116	24.1108086	320.0212831	0.0000000	8.2904876	1.8867547	2.6941333E‐02 0.02694133309777930000	7
	RIME	1.6612767	3.7694202	14.1406474	24.4780117	0.0014759	1.6114495	2.9502557	2.7798190E‐03 0.00277981896705749000	3
DDM‐M2	**WLS**	0.1533142	0.0003271	0.0493465	998.4440835	0.2690334	1.8064970	1.5191142	**1.8131122E‐03** **0.00181311224929325000**	**1**
	SWO	0.1535954	0.1045585	0.1171374	2413.2791543	0.2329281	1.8427682	1.6307017	1.9116675E‐03 0.00191166754905774000	2
	SGA	0.1539132	1.1708342	0.0007109	4712.1553710	0.1679658	1.9289534	1.4819623	2.1679539E‐03 0.00216795388413204000	6
	RECAA	0.1530000	0.0970000	0.1970000	4941.1120000	0.2160000	1.8000000	1.7000000	1.9812436E‐03 0.00198124362000250000	4
	FFA	0.1528320	0.1356060	0.0000010	4998.9059889	0.2336152	1.6293747	1.1201596	1.9261844E‐03 0.00192618437052597000	3
	KOA	0.1549617	0.0000010	1.3139982	3754.6970885	0.1435644	1.3854771	1.9453309	2.9853024E‐03 0.00298530241292956000	7
	RIME	0.1536523	0.4686506	0.0000010	4831.9592004	0.2005171	1.7873179	1.2301144	2.0204715E‐03 0.00202047149008826000	5
DDM‐M3	**WLS**	1.0322098	3.3932330	0.0000000	23.5774096	0.0334139	1.3485179	33.1751703	**2.3860126E‐03** **0.00238601264329269000**	**1**
	SWO	1.0315481	15.5781516	33.9636572	1113.1344147	0.0239834	44.0381815	1.6480662	8.3268694E‐03 0.00832686936863486000	5
	SGA	1.0337685	41.3754091	1.9571746	19.4985611	0.0345924	2.8013404	1.2940238	2.7072443E‐03 0.00270724430101520000	2
	RECAA	1.0268870	4.4100000	49.9871001	64.8130230	0.0329996	1.3766000	5.5522578	3.0954748E‐03 0.00309547475554793000	3
	FFA	1.0267597	49.9995876	7.3484070	1999.9835023	0.0313350	49.9995876	1.4350748	3.5592427E‐03 0.00355924273211639000	4
	KOA	1.0429873	50.0000000	50.0000000	1209.8520229	0.0179306	1.7135554	6.8230809	2.2605228E‐02 0.02260522824949120000	7
	RIME	1.0375009	49.6414253	50.0000000	2000.0000000	0.0186891	1.9590902	1.7719089	1.2636816E‐02 0.01263681622166210000	6
DDM‐M4	**WLS**	7.4725299	0.0000000	2.3349952	22.2199182	0.0045946	40.8507937	1.2756548	**1.6600603E‐02** **0.01660060312508560000**	**1**
	SWO	7.4925957	26.7721041	9.3994695	883.2078339	0.0038516	33.8001696	1.4057421	2.7741644E‐02 0.02774164406542010000	4
	SGA	7.4979914	11.8979152	1.3002343	808.6859932	0.0036992	1.4303434	6.3773512	3.1142751E‐02 0.03114275140960080000	5
	RECAA	7.4700000	1.9300000	43.2931244	28.9095600	0.0047000	1.2600000	3.0600000	1.7199151E‐02 0.01719915138449140000	2
	FFA	7.4580095	4.6697133	49.9982929	1499.4690944	0.0041495	1.3370562	49.9825569	2.3552293E‐02 0.02355229341013970000	3
	KOA	7.4947766	24.4898705	41.3421417	1231.6683675	0.0028429	41.4634881	1.5785420	6.1288152E‐02 0.06128815198389030000	6
	RIME	7.5568343	48.9388503	50.0000000	1500.0000000	0.0023308	1.8880433	1.6249289	6.4693801E‐02 0.06469380066544590000	7
DDM‐M5	**WLS**	8.1586202	0.0164558	6.2132419	3.5317798	0.0039446	1.1926490	21.0318915	**4.5653502E‐02** **0.04565350177253300000**	**1**
	SWO	8.1162590	3.1053821	4.4105239	375.5364734	0.0017573	1.6135700	89.1770827	9.6226507E‐02 0.09622650737581370000	5
	SGA	8.1271849	0.4390872	1.0629896	8.1176079	0.0026585	1.4249608	79.9441846	7.4170292E‐02 0.07417029166118460000	4
	RECAA	8.1000000	0.1963000	9.8139600	15.6100561	0.0030440	1.3600000	16.7964100	6.8709983E‐02 0.06870998299108410000	3
	FFA	8.0898284	0.0000010	0.0860224	499.5382568	0.0034112	1.0000108	1.2987822	6.5762630E‐02 0.06576263008602560000	2
	KOA	8.2091586	4.0561249	10.0000000	123.6709752	0.0000000	128.0000000	1.7363696	1.8813316E‐01 0.18813316234575600000	7
	RIME	8.1218479	10.0000000	9.8271861	500.0000000	0.0003945	1.7813933	1.9634805	1.1169153E‐01 0.11169152571106100000	6
DDM‐M6	WLS	5.4877624	162.6430168	0.0100000	999.9999999	0.0005993	1.0000000	3.0000000	7.5783329E‐02 0.07578332920045290000	2
	SWO	5.4984520	215.8231459	43.3829894	200.9398572	0.0002633	1.0368231	1.1195716	7.6566547E‐02 0.07656654707723740000	5
	SGA	5.4877607	0.0100027	162.6296402	925.2285253	0.0005997	1.0005431	1.0000000	7.5783451E‐02 0.07578345091547710000	3
	RECAA	5.5000000	178.0000000	200.1034000	97.9133529	0.0004973	1.0100000	1.7420000	7.6440766E‐02 0.07644076634690150000	4
	**FFA**	5.4877624	0.0100000	162.6330250	1000.0000000	0.0005993	1.0000000	1.0000000	**7.5783327E‐02** **0.07578332684230280000**	**1**
	KOA	5.3963575	744.6102157	135.1756232	130.0263262	0.0001011	1.4956053	1.0000000	1.0411741E‐01 0.10411741239588100000	7
	RIME	5.4946412	0.0978967	280.8076231	999.9555164	0.0001851	1.0067001	1.0549768	7.6675961E‐02 0.07667596121962360000	6

Figure 9Experimental measurement data versus estimated by WLS algorithm for DDM‐M.

**Figure:**
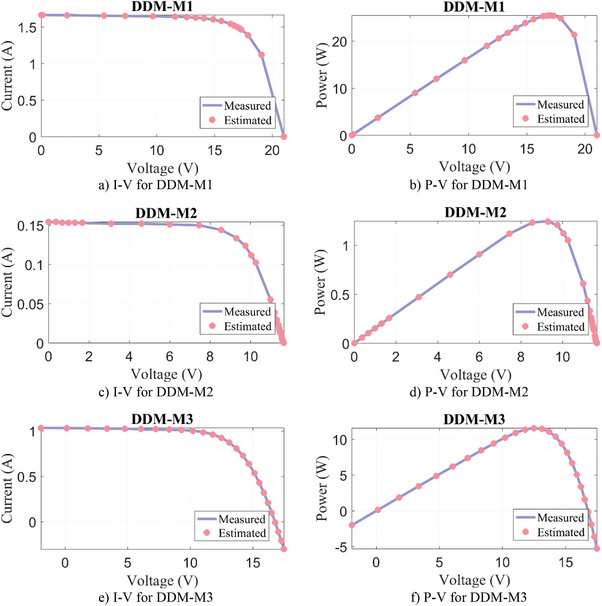

**Figure:**
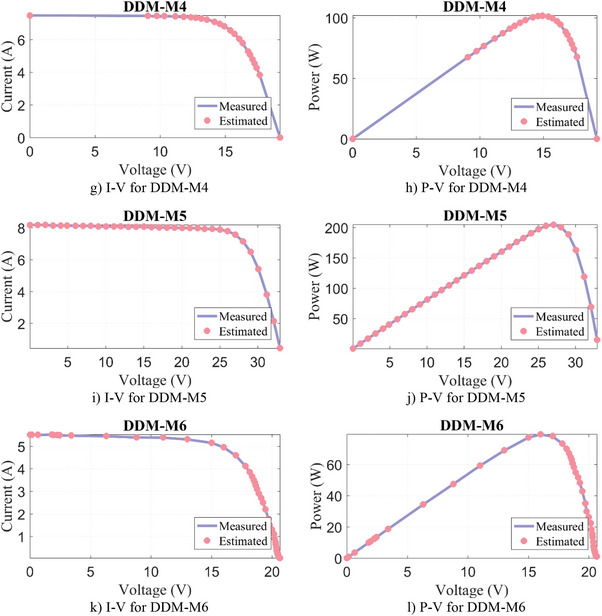

#### 4.5.6 TDM‐M

The parameter extraction results of TDM‐M1‐M6 are given in **Table** **12**, which presents the estimated results for nine variables and the objective function/RMSE value for TDM. In TDM‐M1‐M4 PV models, the lowest RMSE result was produced with the WLS. While the most successful result in TDM‐M5 is calculated with the RECAA algorithm, it is obtained by the FFA in TDM‐M6. When the result calculated with the WLS algorithm is compared with the RECAA and FFA, it is seen that there is a difference of 1.65E+01 and 8.44E‐02, respectively. The comparison of the measured and estimated data is given in **Figure** **10**, which indicates a good match again.

**Table 12:** PV parameter extraction results for TDM‐M1‐M6.

PV model	Algorithm	*I*_ph_ [A]	*I*_*o*1_ [µA]	*I*_*o*2_ [µA]	*I*_*o*3_ [µA]	*R*_sh_ [Ω]	*R_s_* [Ω]	*α*_1_	*α*_2_	*α*_3_	RMSE	RMSE rank
TDM‐M1	**WLS**	1.6637398	1.4889801	21.1529296	18.6523502	16.6634581	0.0044907	1.5043725	24.6861583	3.0767227	**1.7202020E‐03** **0.00172020201278825000**	**1**
	SWO	1.6583434	15.9978402	34.8452323	6.9878944	45.9962659	0.0000350	18.2384626	40.6605005	1.6901524	4.1872946E‐03 0.00418729459076107000	6
	SGA	1.6608946	5.4109940	0.9585294	0.0000408	25.4703051	0.0003234	1.6567466	7.2637016	1.1045638	3.2232019E‐03 0.00322320189565988000	4
	RECAA	1.6600000	15.7401174	45.6700000	3.4700000	26.6100000	0.0022000	5.4000000	6.2200000	1.6000000	2.7033603E‐03 0.00270336034447264000	3
	FFA	1.6628387	0.0000000	0.0000000	2.5224771	20.2501339	0.0035708	1.0000000	59.9985272	1.5621554	2.4492483E‐03 0.00244924831284717000	2
	KOA	1.6569577	34.6002116	30.5413829	18.1976551	390.0260858	0.0000000	31.5772691	10.0694458	1.8340027	1.8011778E‐02 0.01801177820764410000	7
	RIME	1.6583059	11.8687112	0.1385816	32.9559021	43.7991237	0.0007153	1.8375721	1.3661111	48.7926580	4.0036878E‐03 0.00400368782476879000	5
TDM‐M2	**WLS**	0.1531875	0.0030300	0.0066775	0.0274719	3080.4044138	0.2765851	1.6661450	1.4595588	1.4911241	**1.8073165E‐03** **0.00180731651184192000**	**1**
	SWO	0.1536107	0.5266247	0.2386287	0.0681598	4838.8495970	0.1861159	1.9909188	1.7532368	1.9735104	2.0886029E‐03 0.00208860285302760000	6
	SGA	0.1534534	0.2219640	0.0272735	0.0730751	4424.2646765	0.2318716	1.9575323	1.5789287	1.6301756	1.9212881E‐03 0.00192128806370094000	3
	RECAA	0.1540000	0.1340000	0.0690000	0.1600000	2775.7054908	0.2130000	1.6730000	1.7900000	1.8850000	2.0024374E‐03 0.00200243736016851000	4
	FFA	0.1531960	0.0820271	0.0000010	0.0000010	3976.4659008	0.2509343	1.5720626	1.6862969	1.9993517	1.8565617E‐03 0.00185656173112009000	2
	KOA	0.1520480	0.0000022	0.0000036	0.8631004	2295.9399484	0.2010436	1.0000000	1.4133279	1.8931275	3.2263972E‐03 0.00322639719294191000	7
	RIME	0.1535524	0.4327202	0.0000010	0.0000010	3465.1354595	0.2019291	1.7761535	1.6429826	1.4934112	2.0121518E‐03 0.00201215177044652000	5
TDM‐M3	**WLS**	1.0322098	0.0000001	3.3932328	0.0000000	23.5774111	0.0334139	38.0755608	1.3485179	26.9582547	**2.3860126E‐03** **0.00238601264329288000**	**1**
	SWO	1.0318655	28.9373069	17.8523474	13.3609773	470.5466580	0.0277422	39.4514951	1.5511972	10.6865402	5.8215380E‐03 0.00582153804465219000	6
	SGA	1.0332176	0.1932885	0.0168666	6.3404177	21.9735056	0.0359763	1.7128379	1.0046742	1.4767498	2.9473426E‐03 0.00294734263651377000	2
	RECAA	1.0300000	49.7960119	7.4200000	49.3110000	61.8570000	0.0310098	34.4337774	1.4368000	39.5966396	3.3195178E‐03 0.00331951783627347000	3
	FFA	1.0288245	49.9949844	0.0000000	7.7452869	1999.7985446	0.0311475	37.6204708	49.9949764	1.4411988	3.9755199E‐03 0.00397551992985051000	5
	KOA	0.9681853	24.4879758	23.0510609	35.5245512	1410.9018018	0.0129323	7.7373671	12.4170243	1.6760203	5.2672093E‐02 0.05267209276077310000	7
	RIME	1.0278080	10.1424019	43.9052602	0.2525894	1999.3560088	0.0349031	43.0410675	1.9093261	1.1454069	3.5175188E‐03 0.00351751882291623000	4
TDM‐M4	**WLS**	7.4725299	0.0000004	0.0000008	2.3349948	22.2199176	0.0045946	41.9941350	44.3862263	1.2756547	**1.6600603E‐02** **0.01660060312508790000**	**1**
	SWO	7.5087703	42.1595496	20.7301967	45.1963919	1425.0814291	0.0033188	29.4988556	1.4923604	18.8389824	4.0462433E‐02 0.04046243293176870000	6
	SGA	7.4877277	0.0077077	0.0319672	15.4452646	1058.3203591	0.0044588	2.0550038	1.0392551	1.5203181	2.4262370E‐02 0.02426236995333600000	4
	RECAA	7.4600000	48.1400000	49.7030800	2.4200000	210.9468008	0.0045300	3.2500000	3.2590000	1.2790000	1.7787877E‐02 0.01778787742471250000	2
	FFA	7.4595329	0.0000000	0.0000000	3.5619217	893.4838254	0.0043429	49.9954855	49.9954855	1.3123402	1.8851797E‐02 0.01885179703975780000	3
	KOA	7.5194738	16.3267832	0.2286392	47.5304866	522.3139218	0.0040082	24.1494213	3.2393657	1.5984919	2.1773480E‐01 0.21773480392582900000	7
	RIME	7.5015254	44.8892003	0.2560429	47.5403596	1303.3505200	0.0047055	1.9683020	1.1268300	1.9457465	2.4281503E‐02 0.02428150278545280000	5
TDM‐M5	WLS	8.1275023	8.5290245	7.7376715	0.1768705	6.6023523	0.0030515	54.5473430	89.4586246	1.3520151	6.6005074E‐02 0.06600507429694810000	2
	SWO	8.1173513	5.6239719	3.8343422	6.8407265	26.5159985	0.0015199	65.9112909	1.6354779	12.7749167	9.5313953E‐02 0.09531395320837790000	5
	SGA	8.1245193	0.0255760	0.0000010	0.5729802	8.9989804	0.0025156	4.8248132	1.0078750	1.4479642	7.6646250E‐02 0.07664625018474330000	4
	**RECAA**	8.0900000	9.7042000	8.0448890	0.0099000	7.7428311	0.0040000	18.7521989	8.5169000	1.1620000	**5.6669806E‐02** **0.05666980577037910000**	**1**
	FFA	8.1133287	9.9988883	0.0000010	0.2054523	19.9502696	0.0032926	46.1331146	127.9857702	1.3640716	7.2098375E‐02 0.07209837462722390000	3
	KOA	7.7069898	2.2871101	9.0237169	0.0000010	500.0000000	0.0000000	128.0000000	1.7571162	53.2151658	4.2060631E‐01 0.42060630961460100000	7
	RIME	8.1278043	10.0000000	9.8358696	10.0000000	499.9849440	0.0000000	1.8881906	1.9805539	1.8616378	1.1763480E‐01 0.11763480056879800000	6
TDM‐M6	WLS	5.4878567	2.4313856	0.0103163	174.5116018	929.6203372	0.0005480	2.0233697	2.8514568	1.0067993	7.5847251E‐02 0.07584725097214020000	2
	SWO	5.5002731	0.3831098	4.4557029	408.3992781	883.2254820	0.0000015	2.9014394	2.5639234	1.0966757	7.9200689E‐02 0.07920068932127080000	7
	SGA	5.4925030	0.0741948	265.6784660	0.0104353	953.5249911	0.0002135	1.0343541	1.0490949	1.5365422	7.6552808E‐02 0.07655280799213060000	3
	RECAA	5.4980000	77.9000000	182.1170000	265.7910000	336.5990478	0.0003200	1.2650000	1.0161240	2.6906234	7.6946282E‐02 0.07694628178795020000	4
	**FFA**	5.4877636	162.6231113	0.0100000	0.0100000	1000.0000000	0.0005993	1.0000000	1.0000000	1.0000000	**7.5783327E‐02** **0.07578332685323580000**	**1**
	KOA	5.4978341	400.8054174	240.9373223	185.3981383	470.4778320	0.0004590	2.5639841	1.6632355	1.0157018	7.7932823E‐02 0.07793282272336880000	6
	RIME	5.4964256	321.0978213	0.0100000	28.3003822	1000.0000000	0.0000371	1.0905880	2.9999599	1.0000000	7.7325150E‐02 0.07732514994790620000	5

Figure 10Experimental measurement data versus estimated by WLS algorithm for TDM‐M.

**Figure:**
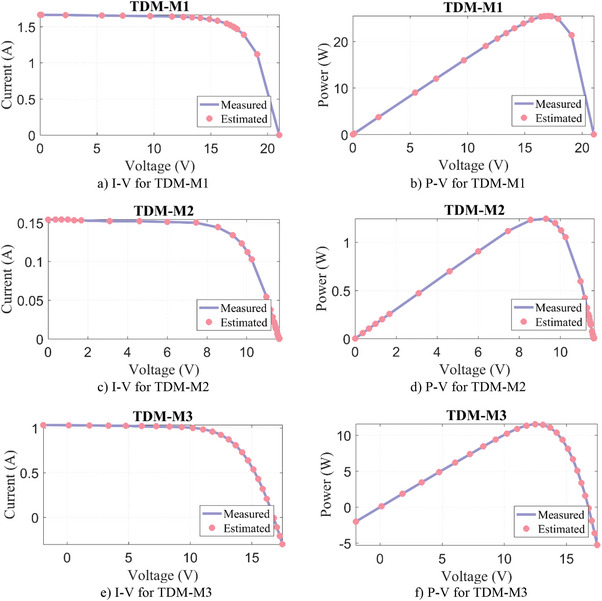

**Figure:**
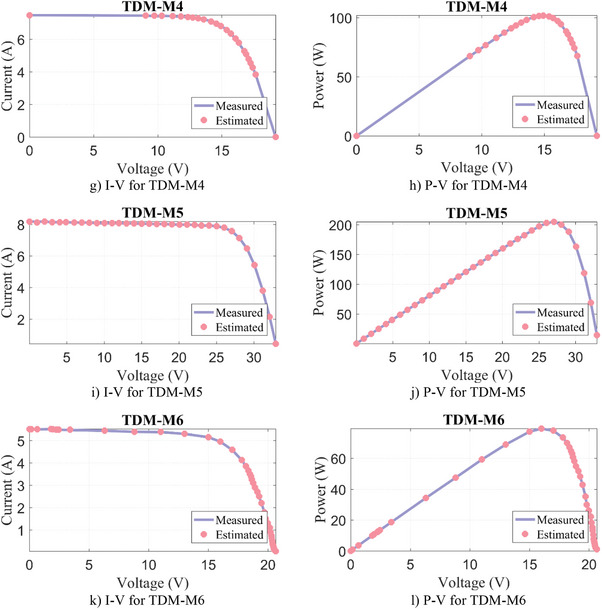

### 4.6 Evaluation Metrics‐Based Assessment

For the statistical assessment of PV parameter extraction performed so far, the algorithms have been run for 10000 iterations and 30 runs. The results of these 30 runs have been evaluated in terms of average, maximum, minimum, standard deviation evaluation metrics and presented under two subsections below: computational accuracy and computational time.

#### 4.6.1 Computational Accuracy

The performance of seven algorithms (WLS, SWO, SGA, RECAA, RECAA, FFA, KOA, and RIME) was evaluated on a total of 24 models consisting of two SDM‐C, two DDM‐C, two TDM‐C, six SDM‐M, six DDM‐M, and six TDM‐M. Hence, the computational accuracy (RMSE) results for 30 runs are given in **Table** **13**. In addition to these detailed results, a general evaluation is given in the bottom part of Table 13. For 24 models, the average, maximum, minimum, and standard deviation evaluation metrics are ranked from smallest to largest according to the results obtained by the algorithms. The smallest value, that is, the most successful result, is given the order 1 and the largest value is given the order 7. The rank orders obtained from the results produced by the algorithms were taken, and these ranks were averaged for each evaluation metric. This overall evaluation allows us to comment on the average performance of the algorithms in achieving the smallest RMSE in each evaluation metric. Accordingly, the WLS algorithm achieved the smallest, namely, the most successful RMSE value in all four‐evaluation metrics. According to both the individual results and the overall evaluation results, it is seen that the WLS algorithm exhibits a stable and successful behavior with the results it produces for the solution of the problem.

**Table 13:** Computational accuracy results based on evaluation metrics (average, maximum, minimum, standard deviation) for 30 runs.

PV model	Algorithm	Average	Rank	Maximum	Rank	Minimum	Rank	Standard deviation	Rank
SDM‐C1	WLS	9.8602188E‐04	1	9.8602188E‐04	1	9.8602188E‐04	1	2.5973141E‐17	1
	SWO	2.8645122E‐03	3	4.4864786E‐03	3	1.1945634E‐03	4	9.4693627E‐04	6
	SGA	3.1529808E‐03	4	4.9243593E‐03	4	1.1672911E‐03	3	8.6419079E‐04	4
	RECAA	3.9681335E‐03	5	5.2347974E‐03	5	1.8383538E‐03	6	8.6835604E‐04	5
	FFA	2.3251542E‐03	2	2.5436382E‐03	2	1.5837163E‐03	5	1.7828459E‐04	2
	KOA	5.1623687E‐03	6	6.3846673E‐03	6	3.7806581E‐03	7	6.0765056E‐04	3
	RIME	5.9356607E‐03	7	7.7247067E‐03	7	1.1543237E‐03	2	1.7810859E‐03	7
SDM‐C2	WLS	2.3861506E‐04	1	2.4697146E‐04	1	2.2888264E‐04	1	4.7931794E‐06	1
	SWO	5.3190111E‐04	3	6.2377434E‐04	3	2.8701953E‐04	7	8.4607603E‐05	3
	SGA	4.6414920E‐03	4	2.5399581E‐02	5	2.3164078E‐04	3	9.4438811E‐03	4
	RECAA	4.0480257E‐04	2	4.8220583E‐04	2	2.6379806E‐04	6	6.1344166E‐05	2
	FFA	1.9538889E‐02	7	2.5399581E‐02	5	2.3115723E‐04	2	1.0805025E‐02	6
	KOA	6.2940080E‐03	5	2.5399836E‐02	7	2.4753936E‐04	5	1.0721290E‐02	5
	RIME	8.7859100E‐03	6	2.5389997E‐02	4	2.4525177E‐04	4	1.1857179E‐02	7
DDM‐C1	WLS	1.0642178E‐03	1	1.7237478E‐03	1	9.8492457E‐04	1	1.8996123E‐04	1
	SWO	3.5579924E‐03	4	6.0388066E‐03	5	1.4689848E‐03	3	1.1712658E‐03	6
	SGA	2.4665061E‐03	2	4.5262486E‐03	3	1.0457107E‐03	2	9.5146028E‐04	4
	RECAA	4.3723045E‐03	6	5.8403733E‐03	4	2.8100525E‐03	5	8.1244953E‐04	3
	FFA	2.5681490E‐03	3	4.0245170E‐03	2	2.0438739E‐03	4	3.7452715E‐04	2
	KOA	6.8313257E‐03	7	8.7547891E‐03	6	3.9968795E‐03	7	1.0924578E‐03	5
	RIME	4.2684392E‐03	5	8.7707323E‐03	7	2.9625826E‐03	6	1.3552609E‐03	7
DDM‐C2	WLS	2.5943946E‐04	1	3.4437278E‐04	1	2.2572566E‐04	1	2.6944019E‐05	1
	SWO	6.0427338E‐04	4	6.9362277E‐04	4	3.7019032E‐04	7	8.8246710E‐05	3
	SGA	4.7302284E‐04	2	6.8730794E‐04	3	2.4085298E‐04	3	1.3641629E‐04	4
	RECAA	4.7715074E‐04	3	6.0251559E‐04	2	2.8176757E‐04	5	7.9941757E‐05	2
	FFA	1.8698554E‐02	7	2.5399581E‐02	6	2.2996822E‐04	2	1.1302372E‐02	6
	KOA	7.6468578E‐03	5	2.5401032E‐02	7	3.0436648E‐04	6	1.0949938E‐02	5
	RIME	7.8986400E‐03	6	2.5304165E‐02	5	2.4845280E‐04	4	1.1461858E‐02	7
TDM‐C1	WLS	1.1267464E‐03	1	2.2509815E‐03	1	9.8357850E‐04	1	2.7021291E‐04	1
	SWO	4.0662259E‐03	6	6.6966920E‐03	6	1.3837265E‐03	3	1.3324559E‐03	7
	SGA	1.7314253E‐03	2	3.3536174E‐03	2	1.1064728E‐03	2	5.6352105E‐04	4
	RECAA	3.6047497E‐03	5	5.6631077E‐03	5	1.5978908E‐03	5	1.0230062E‐03	6
	FFA	2.9963816E‐03	3	4.3544117E‐03	3	2.3352563E‐03	6	4.9494227E‐04	2
	KOA	7.9305050E‐03	7	9.0153031E‐03	7	6.6539123E‐03	7	5.5516944E‐04	3
	RIME	3.4992913E‐03	4	5.0087863E‐03	4	1.4297456E‐03	4	9.0894210E‐04	5
TDM‐C2	WLS	2.8424985E‐04	1	3.6277263E‐04	1	2.4124296E‐04	3	3.2220466E‐05	1
	SWO	6.2783874E‐04	4	7.4002172E‐04	4	4.6376535E‐04	7	6.8151079E‐05	2
	SGA	4.9535989E‐04	2	6.7818172E‐04	3	2.2786571E‐04	1	1.4826890E‐04	4
	RECAA	5.0684496E‐04	3	6.5967242E‐04	2	3.1418250E‐04	6	8.1551502E‐05	3
	FFA	2.1211688E‐02	7	2.5399581E‐02	6	2.4351849E‐04	4	9.5245042E‐03	5
	KOA	1.3983926E‐02	6	2.5404512E‐02	7	2.7972752E‐04	5	1.2416752E‐02	7
	RIME	7.0331268E‐03	5	2.5296608E‐02	5	2.3162754E‐04	2	1.1014416E‐02	6
SDM‐M1	WLS	1.7298137E‐03	1	1.7298137E‐03	1	1.7298137E‐03	1	4.6095633E‐18	1
	SWO	4.4601130E‐03	5	6.8195657E‐03	4	2.9468989E‐03	5	9.4514474E‐04	4
	SGA	3.4280624E‐03	4	1.4374434E‐02	5	1.8744285E‐03	4	2.1042131E‐03	5
	RECAA	2.4611922E‐03	3	2.9341259E‐03	3	1.7995003E‐03	3	2.5041529E‐04	3
	FFA	1.8718841E‐03	2	2.2500190E‐03	2	1.7448235E‐03	2	1.0453619E‐04	2
	KOA	2.1273235E‐02	6	3.4480421E‐02	7	1.0064476E‐02	6	7.5250588E‐03	7
	RIME	2.9023063E‐02	7	3.1417615E‐02	6	1.6464152E‐02	7	2.8507102E‐03	6
SDM‐M2	WLS	1.7902521E‐03	1	1.8001259E‐03	1	1.7750971E‐03	1	6.6558993E‐06	1
	SWO	2.0219275E‐03	6	2.1329341E‐03	4	1.8890738E‐03	5	6.7548895E‐05	4
	SGA	2.0012226E‐03	4	2.1554678E‐03	5	1.8206914E‐03	3	7.2248099E‐05	5
	RECAA	1.9235565E‐03	3	2.0238427E‐03	2	1.8227006E‐03	4	5.8122892E‐05	3
	FFA	1.8810812E‐03	2	2.0308476E‐03	3	1.7964968E‐03	2	7.4633811E‐05	6
	KOA	2.1819973E‐03	7	2.2596116E‐03	7	2.0970752E‐03	7	4.5771196E‐05	2
	RIME	2.0202478E‐03	5	2.2522734E‐03	6	1.8935118E‐03	6	1.0342248E‐04	7
SDM‐M3	WLS	2.3860126E‐03	1	2.3860126E‐03	1	2.3860126E‐03	1	1.5295031E‐17	1
	SWO	5.2326503E‐03	4	7.0801915E‐03	4	2.8721849E‐03	4	1.1256596E‐03	5
	SGA	6.3172833E‐03	5	1.1533018E‐02	6	2.8209323E‐03	3	2.7803732E‐03	6
	RECAA	3.2815692E‐03	2	4.2314837E‐03	3	2.6816374E‐03	2	3.9054188E‐04	4
	FFA	3.4840010E‐03	3	3.6799110E‐03	2	3.1377787E‐03	5	1.1271886E‐04	2
	KOA	2.0224297E‐02	7	4.3271774E‐02	7	7.7749715E‐03	6	9.1685904E‐03	7
	RIME	9.7327828E‐03	6	9.7972509E‐03	5	8.5789813E‐03	7	2.3603218E‐04	3
SDM‐M4	WLS	1.6600603E‐02	1	1.6600603E‐02	1	1.6600603E‐02	1	7.1747557E‐17	1
	SWO	3.5051658E‐02	5	4.9395016E‐02	4	1.6662281E‐02	2	7.0163237E‐03	5
	SGA	2.9008442E‐02	4	5.0060552E‐02	5	1.7304043E‐02	5	7.1544543E‐03	6
	RECAA	1.8656848E‐02	3	2.1537230E‐02	3	1.6892205E‐02	4	1.1336193E‐03	3
	FFA	1.7348309E‐02	2	1.9388136E‐02	2	1.6797416E‐02	3	5.0883689E‐04	2
	KOA	1.0670173E‐01	7	2.8031525E‐01	7	4.9032159E‐02	7	5.5941754E‐02	7
	RIME	5.4407308E‐02	6	5.5345451E‐02	6	4.8926975E‐02	6	1.4947081E‐03	4
SDM‐M5	WLS	4.2386278E‐02	1	4.9181571E‐02	1	3.6198065E‐02	3	3.4216011E‐03	1
	SWO	9.1580759E‐02	5	1.0500234E‐01	5	8.1843288E‐02	6	5.9460045E‐03	2
	SGA	7.2645746E‐02	4	8.4845530E‐02	4	5.3594453E‐02	5	7.0645448E‐03	4
	RECAA	5.8232998E‐02	2	7.1425050E‐02	3	4.4601903E‐02	4	6.3540044E‐03	3
	FFA	5.8573999E‐02	3	6.3945820E‐02	2	1.7600576E‐02	1	8.2528237E‐03	5
	KOA	2.8779186E‐01	7	1.4164604E+00	7	1.0174571E‐01	7	3.3127943E‐01	7
	RIME	1.0219026E‐01	6	1.0516901E‐01	6	3.0156819E‐02	2	1.3643844E‐02	6
SDM‐M6	WLS	7.5783327E‐02	1	7.5783327E‐02	1	7.5783327E‐02	1	1.2505950E‐16	1
	SWO	7.6556622E‐02	4	7.7656248E‐02	5	7.5897957E‐02	6	4.8975836E‐04	4
	SGA	7.6619438E‐02	5	7.7266635E‐02	4	7.5783327E‐02	1	5.8840890E‐04	5
	RECAA	7.6100066E‐02	3	7.6351702E‐02	3	7.5869540E‐02	5	1.0658768E‐04	3
	FFA	7.5783327E‐02	1	7.5783327E‐02	1	7.5783327E‐02	1	1.2635385E‐16	2
	KOA	7.7644008E‐02	6	8.0482003E‐02	6	7.6166761E‐02	7	1.0736712E‐03	6
	RIME	9.4957633E‐02	7	1.2215507E‐01	7	7.5783331E‐02	4	2.0509445E‐02	7
DDM‐M1	WLS	1.7190173E‐03	1	1.7297552E‐03	1	1.7148900E‐03	1	4.4767263E‐06	1
	SWO	6.4663813E‐03	5	2.2121364E‐02	5	3.1490602E‐03	6	4.6659215E‐03	5
	SGA	2.9998842E‐03	4	3.5547726E‐03	4	1.7609700E‐03	2	4.6823819E‐04	3
	RECAA	2.5132408E‐03	2	3.1802450E‐03	2	1.9831637E‐03	4	3.0307784E‐04	2
	FFA	2.5769012E‐03	3	3.3499608E‐03	3	1.8557498E‐03	3	5.2044620E‐04	4
	KOA	2.5669449E‐02	7	4.5820632E‐02	7	7.6713940E‐03	7	8.0977719E‐03	6
	RIME	9.0362085E‐03	6	3.0695126E‐02	6	2.7798190E‐03	5	8.5591656E‐03	7
DDM‐M2	WLS	1.8184558E‐03	1	1.8749207E‐03	1	1.7792298E‐03	1	2.1955033E‐05	1
	SWO	2.0680390E‐03	6	2.1862764E‐03	5	1.9116675E‐03	5	7.7016896E‐05	4
	SGA	2.0066562E‐03	4	2.1679539E‐03	4	1.7920213E‐03	2	1.0153914E‐04	6
	RECAA	1.9427147E‐03	3	2.0147270E‐03	2	1.8567608E‐03	4	4.2048485E‐05	2
	FFA	1.8703774E‐03	2	2.0805976E‐03	3	1.8189351E‐03	3	5.3030580E‐05	3
	KOA	2.4326753E‐03	7	3.1162081E‐03	7	2.1250324E‐03	7	2.7423502E‐04	7
	RIME	2.0337168E‐03	5	2.2517492E‐03	6	1.9156827E‐03	6	9.6922004E‐05	5
DDM‐M3	WLS	2.3860145E‐03	1	2.3860669E‐03	1	2.3860126E‐03	1	9.8970011E‐09	1
	SWO	6.1680312E‐03	5	8.6426535E‐03	5	3.1494655E‐03	5	1.4591768E‐03	5
	SGA	4.4120339E‐03	4	6.3755646E‐03	4	2.7072443E‐03	3	9.5743762E‐04	4
	RECAA	3.2858804E‐03	2	3.8377418E‐03	2	2.6370611E‐03	2	3.2276871E‐04	3
	FFA	3.6019692E‐03	3	4.0873787E‐03	3	2.9846409E‐03	4	2.5537095E‐04	2
	KOA	2.1176029E‐02	7	4.0344437E‐02	7	6.4441067E‐03	7	8.7761355E‐03	7
	RIME	1.0714113E‐02	6	1.2947556E‐02	6	3.6350819E‐03	6	3.0182782E‐03	6
DDM‐M4	WLS	1.6614711E‐02	1	1.7023843E‐02	1	1.6600603E‐02	1	7.7272735E‐05	1
	SWO	3.4862107E‐02	5	4.7518066E‐02	5	1.6846545E‐02	2	9.2722628E‐03	5
	SGA	2.7951997E‐02	4	3.5993920E‐02	4	1.9887920E‐02	6	4.3493994E‐03	4
	RECAA	1.8723296E‐02	3	2.2198327E‐02	2	1.6977955E‐02	4	1.3520320E‐03	2
	FFA	1.8484465E‐02	2	2.4729271E‐02	3	1.6893442E‐02	3	1.8621205E‐03	3
	KOA	1.5011993E‐01	7	3.3992189E‐01	7	6.1288152E‐02	7	6.8590979E‐02	7
	RIME	5.7825133E‐02	6	6.8193261E‐02	6	1.7491099E‐02	5	1.6062825E‐02	6
DDM‐M5	WLS	4.6043687E‐02	1	6.4324532E‐02	1	3.6849339E‐02	2	6.0588393E‐03	3
	SWO	9.1589283E‐02	5	1.0440219E‐01	5	7.0361754E‐02	5	6.4326084E‐03	4
	SGA	6.8024181E‐02	4	8.6815462E‐02	4	1.7600576E‐02	1	1.5339320E‐02	6
	RECAA	6.2278623E‐02	2	7.6118211E‐02	2	4.6575917E‐02	3	6.4699083E‐03	5
	FFA	6.6454381E‐02	3	8.0095709E‐02	3	5.9278189E‐02	4	4.8777774E‐03	1
	KOA	2.3884407E‐01	7	7.0036132E‐01	7	1.0688156E‐01	7	1.6006750E‐01	7
	RIME	1.0943175E‐01	6	1.1300837E‐01	6	9.3164273E‐02	6	4.9456736E‐03	2
DDM‐M6	WLS	7.5783329E‐02	1	7.5783329E‐02	1	7.5783329E‐02	3	2.7896389E‐16	1
	SWO	7.7565043E‐02	6	8.4593399E‐02	6	7.6115052E‐02	6	1.8148216E‐03	6
	SGA	7.6894357E‐02	5	7.7287355E‐02	4	7.5783327E‐02	1	5.0144341E‐04	4
	RECAA	7.6226494E‐02	3	7.6688225E‐02	3	7.5940241E‐02	5	1.9875889E‐04	3
	FFA	7.5787076E‐02	2	7.5893201E‐02	2	7.5783327E‐02	1	2.0046845E‐05	2
	KOA	8.3333857E‐02	7	1.0677419E‐01	7	7.6339276E‐02	7	7.4792572E‐03	7
	RIME	7.6862004E‐02	4	7.9181672E‐02	5	7.5783362E‐02	4	7.2095245E‐04	5
TDM‐M1	WLS	1.7813544E‐03	1	2.3308755E‐03	1	1.7169302E‐03	1	1.6204365E‐04	1
	SWO	9.3736913E‐03	6	2.5536206E‐02	6	1.7667726E‐03	2	7.1907852E‐03	6
	SGA	2.9868232E‐03	3	4.3705881E‐03	3	1.7859577E‐03	3	5.6942430E‐04	3
	RECAA	2.5110293E‐03	2	2.9031343E‐03	2	1.8906864E‐03	5	2.7704759E‐04	2
	FFA	3.1674993E‐03	4	4.8073476E‐03	4	1.8454235E‐03	4	5.8921513E‐04	4
	KOA	2.8708752E‐02	7	5.9772368E‐02	7	1.7324402E‐02	7	8.9854892E‐03	7
	RIME	4.5398480E‐03	5	1.2159974E‐02	5	2.2624639E‐03	6	1.7391127E‐03	5
TDM‐M2	WLS	1.8238242E‐03	1	1.8831434E‐03	1	1.7920960E‐03	2	2.2404670E‐05	1
	SWO	2.0904584E‐03	6	2.2005477E‐03	6	1.9124076E‐03	6	7.3045817E‐05	5
	SGA	1.9529337E‐03	3	2.1560007E‐03	4	1.7584459E‐03	1	1.0928933E‐04	6
	RECAA	1.9588495E‐03	4	2.0670611E‐03	3	1.8234774E‐03	4	6.2247422E‐05	2
	FFA	1.9057686E‐03	2	2.0450185E‐03	2	1.8045572E‐03	3	6.7798303E‐05	3
	KOA	2.4637644E‐03	7	3.2263972E‐03	7	2.1925184E‐03	7	2.5671558E‐04	7
	RIME	2.0207567E‐03	5	2.1956571E‐03	5	1.8999514E‐03	5	7.0537403E‐05	4
TDM‐M3	WLS	2.4319503E‐03	1	3.3867935E‐03	1	2.3860126E‐03	1	1.8636377E‐04	1
	SWO	6.3048260E‐03	5	9.0271826E‐03	5	3.4218606E‐03	6	1.2084531E‐03	5
	SGA	4.0938385E‐03	4	6.1682987E‐03	4	2.4303651E‐03	2	7.6791043E‐04	4
	RECAA	3.1758255E‐03	2	3.9501657E‐03	2	2.6734969E‐03	3	3.1162741E‐04	2
	FFA	4.0285482E‐03	3	5.0529445E‐03	3	3.3961427E‐03	5	4.4510879E‐04	3
	KOA	3.2218745E‐02	7	5.7343254E‐02	7	1.1263924E‐02	7	1.5258636E‐02	7
	RIME	9.7663645E‐03	6	1.4719275E‐02	6	2.9298052E‐03	4	4.0726553E‐03	6
TDM‐M4	WLS	1.6668656E‐02	1	1.8642175E‐02	1	1.6600603E‐02	1	3.7273824E‐04	1
	SWO	3.9607469E‐02	5	5.4873253E‐02	5	1.9184582E‐02	6	8.9185878E‐03	5
	SGA	2.5750714E‐02	4	3.3841604E‐02	4	1.8747656E‐02	4	4.0356819E‐03	4
	RECAA	1.8680504E‐02	2	2.1213714E‐02	2	1.7110874E‐02	2	9.7950379E‐04	2
	FFA	2.1664307E‐02	3	3.2358820E‐02	3	1.7178330E‐02	3	3.7885795E‐03	3
	KOA	1.6891730E‐01	7	3.2254448E‐01	7	7.3254469E‐02	7	7.0494475E‐02	7
	RIME	5.3235165E‐02	6	7.5031646E‐02	6	1.8828416E‐02	5	2.1363630E‐02	6
TDM‐M5	WLS	5.6892450E‐02	1	7.9769179E‐02	2	4.3077130E‐02	2	1.0680717E‐02	5
	SWO	9.3554541E‐02	5	1.0352004E‐01	5	8.0100903E‐02	6	5.9698671E‐03	3
	SGA	6.8245078E‐02	3	8.3494347E‐02	4	2.5350975E‐02	1	1.1445918E‐02	6
	RECAA	6.2366027E‐02	2	7.2791144E‐02	1	5.3618682E‐02	3	5.8243712E‐03	2
	FFA	6.9676440E‐02	4	7.9916102E‐02	3	6.1050761E‐02	4	4.9150033E‐03	1
	KOA	2.4237847E‐01	7	6.0986548E‐01	7	1.0888056E‐01	7	1.1476230E‐01	7
	RIME	1.0961922E‐01	6	1.1763480E‐01	6	7.7299152E‐02	5	9.4538929E‐03	4
TDM‐M6	WLS	7.579677E‐02	2	7.612136E‐02	2	7.578333E‐02	1	6.240355E‐05	2
	SWO	7.918257E‐02	6	1.027358E‐01	6	7.623392E‐02	6	4.794106E‐03	6
	SGA	7.696470E‐02	5	7.787428E‐02	4	7.578333E‐02	1	5.138257E‐04	4
	RECAA	7.638932E‐02	3	7.703902E‐02	3	7.614259E‐02	5	2.277995E‐04	3
	FFA	7.578464E‐02	1	7.580437E‐02	1	7.578333E‐02	1	4.344718E‐06	1
	KOA	8.637212E‐02	7	1.203102E‐01	7	7.733189E‐02	7	1.067023E‐02	7
	RIME	7.673248E‐02	4	7.813753E‐02	5	7.578334E‐02	4	6.912357E‐04	5

PV model	Algorithm	Mean rank	Total rank	Mean rank	Total rank	Mean rank	Total rank	Mean rank	Total rank
SDM, DDM, and TDM	**WLS**	**1.0417**	**1**	**1.0833**	**1**	**1.3750**	**1**	**1.2917**	**1**
	SWO	4.9167	5	4.7917	5	5.0000	6	4.5833	5
	SGA	3.7083	4	4.0000	4	2.5833	2	4.5417	4
	RECAA	2.9167	2	2.6250	2	4.1250	4	2.9167	2
	FFA	3.0833	3	2.8750	3	3.1250	3	3.0000	3
	KOA	6.6667	7	6.8750	7	6.7083	7	6.1250	7
	RIME	5.6250	6	5.6667	6	4.7917	5	5.5417	6

In addition to the overall evaluation, we also evaluated the minima of the computational accuracy (RMSE) results calculated by the algorithms for each evaluation metric within the 24 PV models. The first evaluation metric is the average of the computational accuracy (RMSE) results over 30 runs. Among the algorithms, the minimum average was calculated by the WLS algorithm with 2.3861506E‐04. The WLS is followed by RECAA with 4.0480257E‐04, SGA with 4.7302284E‐04, SWO with 5.3190111E‐04, FFA with 1.8703774E‐03, RIME with 2.0202478E‐03, and KOA with 2.1819973E‐03. The WLS algorithm outperforms the closest RECAA by 69.65% and the farthest KOA by 814.44%. The second evaluation metric is the maximum, which calculates the highest value of the RMSE results within 30 runs. It is worth noting that the lowest maximums are considered during the evaluation. Therefore, among the algorithms, the lowest of the maximum is calculated by the WLS with 2.4697146E‐04. The WLS is followed by the RECAA with 4.8220583E‐04, then the SWO with 6.2377434E‐04, SGA with 6.7818172E‐04, FFA with 2.0308476E‐03, RIME with 2.1956571E‐03, and the KOA with 2.2596116E‐03. Correspondingly, the WLS is 95.25% more successful than RECAA, which is the closest, and 814.93% more successful than KOA, which is the farthest. The third evaluation metric is the minimum, which means the smallest value of the RMSE calculated by the algorithms within 30 runs. Here, the lowest minimums are also considered among the minimum results. Hence, among the algorithms, the lowest minimum result is calculated by the WLS with 2.2572566E‐04. This is then followed by SGA with 2.2786571E‐04, FFA with 2.2996822E‐04, RIME with 2.3162754E‐04, KOA with 2.4753936E‐04, RECAA with 2.6379806E‐04, and SWO with 2.8701953E‐04. In brief, the WLS is 0.95% more successful than SGA, which is the closest, and 27.15% more successful than SWO, which is the farthest algorithm. The last evaluation metric is the standard deviation. Among the algorithms, the most consistent and stable result is calculated by the WLS with 4.6095633E‐18. Later, the WLS is followed by SWO with 6.7548895E‐05, SGA with 7.2248099E‐05, RECAA with 4.2048485E‐05, FFA with 1.2635385E‐16, KOA with 4.5771196E‐05, and RIME with 7.0537403E‐05. To summarize, the WLS is 1.47E+15% more stable than SWO, which is the closest, and 1.53E+15% more stable than RIME, which is the farthest.

#### 4.6.2 Computational Time

The mathematical background of the WLS algorithm is presented in Section 3.1 and the inspirations for the SWO, SGA, RECAA, FFA, KOA, and RIME algorithms are presented in Section 3.2. The motivation for each of these seven algorithms is unique and the mathematical backgrounds for which they are designed are different. Therefore, these meta‐heuristic algorithms also differ in the time it takes to solve our PV parameter extraction problem. The time utilization of WLS, SWO, RECAA, FFA, KOA, and RIME algorithms are given by Equations (33)–(38), respectively. The computational time depends on the number of populations (*N*_WLS_) and number of dimensions (*D*) in WLS algorithm,^[^ ^53^ ^]^ number of populations (*N*_SWO_), number of dimensions (*D*), and *t*_SWO,max_ the maximum number of functions evaluation, in SWO algorithm,^[^ ^54^ ^]^ number of populations (*N*_RECAA_), number of dimensions (*D*), *n*_s_ number of smart‐cells, and *n*_ne_ the number of neighbors per smart cell in RECAA algorithm,^[^ ^56^ ^]^ number of populations (*N*_FFA_), number of dimensions (*D*), and *T*_FFA_ maximum number of iterations in FFA algorithm,^[^ ^57^ ^]^ number of populations (*N*_KOA_) and *T*_KOA_ maximum number of iterations in KOA algorithm,^[^ ^58^ ^]^ and lastly the number of populations (*N*_RIME_) in RIME algorithm.^[^ ^59^ ^]^ The computational time results in seconds for 30 runs in solving the PV parameter extraction problem are given in **Table** **14**. It is seen that the SWO algorithm ranks first in average, maximum, minimum, standard deviation evaluation metrics, and this algorithm finds the fastest result. However, when we look at the computational accuracy (RMSE) results of this algorithm, we see that it ranks 5^th^ in average, 5^th^ in maximum, 6^th^ in minimum, and 5^th^ in standard deviation among the seven algorithms. Therefore, computational time is not the only criterion for evaluating algorithms. Both computational accuracy and computational time should be considered together, and a good tradeoff should be achieved. From this point of view, the WLS algorithm, which produces the most successful results in computational accuracy, reaches these results in fairly reasonable times, as seen in Table 14.

(33) $$O\left(\mathrm{WLS}\right)=\;O\left(N_{\mathrm{WLS}}\cdot D\right)$$

(34) $$O\left(\mathrm{SWO}\right)=\;O\left(N_{\mathrm{SWO}}\cdot D\cdot t_{\mathrm{SWO},\max}\right)$$

(35) $$O\left(\mathrm{RECAA}\right)=\;O\left(N_{\mathrm{RECAA}}\cdot D\cdot n_{s}\cdot n_{\mathrm{ne}}\right)$$

(36) $$O\left(\mathrm{FFA}\right)=\;O\left(N_{\mathrm{FFA}}\cdot D\cdot \left(1+3\cdot T_{\mathrm{FFA}}\right)\right)$$

(37) $$O\left(\mathrm{KOA}\right)=\;O\left(N_{\mathrm{KOA}}\cdot T_{\mathrm{KOA}}\right)$$

(38) $$O\left(\mathrm{RIME}\right)=\;O\left(\left(N_{\mathrm{RIME}}+\log N_{\mathrm{RIME}}\right)\cdot N_{\mathrm{RIME}}\right)$$

**Table 14:** Computational time results in seconds based on evaluation metrics (average, maximum, minimum, standard deviation) for 30 runs.

PV model	Algorithm	Average ()	Rank	Maximum	Rank	Minimum	Rank	Standard deviation	Rank
SDM‐C1	WLS	4.7701803E+01	3	5.4656210E+01	3	2.7213969E+01	3	4.6615002E+00	3
	SWO	9.1223755E‐01	1	1.2700557E+00	1	7.7717830E‐01	1	1.1698488E‐01	2
	SGA	5.3778896E+01	4	8.0419978E+01	5	3.2120904E+01	4	1.0044213E+01	5
	RECAA	5.7157886E+02	7	7.2651560E+02	7	5.5624078E+02	7	3.4025303E+01	7
	FFA	1.6484842E+02	6	2.1472556E+02	6	1.5279351E+02	6	1.5090803E+01	6
	KOA	1.1895190E+00	2	1.4463325E+00	2	1.0987678E+00	2	8.4496279E‐02	1
	RIME	6.0282038E+01	5	7.1379189E+01	4	5.0175003E+01	5	5.2964267E+00	4
SDM‐C2	WLS	5.8487762E+01	3	6.5770778E+01	3	4.0885845E+01	3	4.8290566E+00	3
	SWO	1.2717466E+00	1	1.5384729E+00	1	1.1074129E+00	1	1.0795390E‐01	1
	SGA	6.5616508E+01	4	9.4801836E+01	5	4.3296679E+01	4	1.1067395E+01	5
	RECAA	6.0338059E+02	7	7.7470625E+02	7	4.3292699E+02	7	5.1345440E+01	7
	FFA	2.0274060E+02	6	2.7501439E+02	6	1.8421809E+02	6	2.6578259E+01	6
	KOA	1.6181256E+00	2	2.0270166E+00	2	1.3900635E+00	2	1.5654418E‐01	2
	RIME	6.9069236E+01	5	8.3833626E+01	4	5.9438710E+01	5	6.1773380E+00	4
DDM‐C1	WLS	5.4761948E+01	3	6.8734575E+01	3	4.3472071E+01	5	5.5288706E+00	3
	SWO	8.1187043E‐01	1	1.2061072E+00	1	6.8129050E‐01	1	1.0825803E‐01	1
	SGA	6.0803522E+01	5	9.9348266E+01	5	3.8607944E+01	4	1.7555020E+01	5
	RECAA	7.3968715E+02	7	7.9812021E+02	7	6.5668416E+02	7	4.8170215E+01	7
	FFA	2.0143335E+02	6	2.4224396E+02	6	1.6216165E+02	6	1.9126509E+01	6
	KOA	1.3689693E+00	2	1.6599307E+00	2	1.0883777E+00	2	1.6247027E‐01	2
	RIME	5.9424430E+01	4	7.6104649E+01	4	3.7572761E+01	3	6.5380382E+00	4
DDM‐C2	WLS	6.4631715E+01	3	8.6984007E+01	3	3.6995499E+01	3	1.2791995E+01	4
	SWO	1.2967243E+00	1	1.7333710E+00	1	9.4935110E‐01	1	2.5263558E‐01	1
	SGA	8.3986390E+01	5	1.2416736E+02	5	4.6207272E+01	4	1.5753272E+01	5
	RECAA	7.5063365E+02	7	8.9110318E+02	7	4.4241972E+02	7	1.4210011E+02	7
	FFA	2.5851584E+02	6	3.0151342E+02	6	1.9901672E+02	6	2.0273167E+01	6
	KOA	2.4785438E+00	2	3.0645512E+00	2	1.8517110E+00	2	3.6474211E‐01	2
	RIME	7.6205256E+01	4	9.0892596E+01	4	5.6055187E+01	5	7.3811047E+00	3
TDM‐C1	WLS	5.9711722E+01	3	8.5485564E+01	4	5.2789964E+01	4	5.6715765E+00	4
	SWO	8.7662318E‐01	1	1.1866855E+00	1	6.7178500E‐01	1	1.3986157E‐01	1
	SGA	6.6766125E+01	5	9.6460813E+01	5	5.7896255E+01	5	6.4753724E+00	5
	RECAA	7.0939262E+02	7	8.1961588E+02	7	6.1194410E+02	7	2.8007962E+01	7
	FFA	1.9019870E+02	6	2.4642025E+02	6	1.5725006E+02	6	1.9812814E+01	6
	KOA	1.0149442E+00	2	1.4524714E+00	2	7.3984220E‐01	2	1.8659169E‐01	2
	RIME	5.9799777E+01	4	7.1089395E+01	3	4.2772272E+01	3	5.4045872E+00	3
TDM‐C2	WLS	7.2029700E+01	4	1.0278273E+02	4	4.4598687E+01	4	1.3609909E+01	4
	SWO	1.3677260E+00	1	1.9806626E+00	1	9.9492080E‐01	1	2.6629006E‐01	1
	SGA	7.8843548E+01	5	1.2230427E+02	5	5.0649866E+01	5	1.4360454E+01	5
	RECAA	7.6326438E+02	7	9.1491336E+02	7	4.6424186E+02	7	9.6006016E+01	7
	FFA	2.2509759E+02	6	3.2076296E+02	6	1.3888086E+02	6	4.4874417E+01	6
	KOA	1.5576582E+00	2	2.1473199E+00	2	1.0362817E+00	2	3.2904743E‐01	2
	RIME	7.1650473E+01	3	9.1211746E+01	3	4.4233199E+01	3	1.2954981E+01	3
SDM‐M1	WLS	4.4249757E+01	4	5.0948389E+01	4	3.0531129E+01	4	2.9752491E+00	4
	SWO	1.0092337E+00	2	1.2529801E+00	2	7.8407680E‐01	2	1.3765151E‐01	2
	SGA	5.3188668E+01	5	6.2724667E+01	5	4.6655817E+01	5	2.5369457E+00	3
	RECAA	6.5883953E+02	7	8.6482405E+02	7	5.1151752E+02	7	7.8765879E+01	7
	FFA	7.5877978E+01	6	9.9047007E+01	6	7.2147909E+01	6	5.8485539E+00	6
	KOA	5.8140598E‐01	1	6.6889300E‐01	1	5.4286280E‐01	1	3.5763139E‐02	1
	RIME	2.6780936E+01	3	3.6423723E+01	3	2.1711850E+01	3	3.4466762E+00	5
SDM‐M2	WLS	5.4047155E+01	3	6.2583200E+01	3	4.4291606E+01	3	3.1082552E+00	3
	SWO	1.4207239E+00	1	1.9645020E+00	1	1.0105924E+00	1	2.2932039E‐01	1
	SGA	6.1721266E+01	4	8.7668335E+01	5	5.1751050E+01	4	1.0033777E+01	5
	RECAA	5.8867252E+02	7	7.5523134E+02	7	4.9155127E+02	7	4.0121078E+01	7
	FFA	1.7648834E+02	6	2.1415938E+02	6	1.6695954E+02	6	1.0602293E+01	6
	KOA	1.5157384E+00	2	2.4041021E+00	2	1.2640146E+00	2	2.6420592E‐01	2
	RIME	6.4435788E+01	5	8.0150690E+01	4	5.3437469E+01	5	6.9466752E+00	4
SDM‐M3	WLS	4.8438181E+01	4	5.5149789E+01	4	4.3074905E+01	4	1.9890615E+00	4
	SWO	1.1945357E+00	2	1.4089360E+00	2	8.9205620E‐01	2	1.3015940E‐01	2
	SGA	5.5734752E+01	5	6.3736412E+01	5	4.7786690E+01	5	2.4872643E+00	5
	RECAA	6.9032007E+02	7	8.2757280E+02	7	5.7619769E+02	7	7.0089669E+01	7
	FFA	7.6669681E+01	6	9.6615177E+01	6	7.2944027E+01	6	5.9817656E+00	6
	KOA	6.1187110E‐01	1	7.0005930E‐01	1	5.6357230E‐01	1	3.7520207E‐02	1
	RIME	2.3856956E+01	3	2.6717989E+01	3	2.2088292E+01	3	1.2999132E+00	3
SDM‐M4	WLS	4.7346552E+01	3	5.3299761E+01	3	4.2629993E+01	3	1.9556095E+00	3
	SWO	1.1469808E+00	1	1.5920878E+00	2	9.5769320E‐01	1	1.3401832E‐01	2
	SGA	5.3007431E+01	4	7.3593345E+01	5	4.8311643E+01	4	8.7462800E+00	5
	RECAA	5.6262564E+02	7	7.1127253E+02	7	5.4479512E+02	7	3.6331350E+01	7
	FFA	1.5837552E+02	6	2.0069211E+02	6	1.4721054E+02	6	1.4712722E+01	6
	KOA	1.2860023E+00	2	1.5018860E+00	1	1.0784556E+00	2	1.1744848E‐01	1
	RIME	5.8105451E+01	5	6.9006215E+01	4	4.8367069E+01	5	5.5863455E+00	4
SDM‐M5	WLS	5.3629154E+01	3	5.9158844E+01	3	4.5412870E+01	3	2.5804877E+00	3
	SWO	1.0798107E+00	1	1.1884967E+00	1	9.7006790E‐01	1	5.9346035E‐02	1
	SGA	6.1866801E+01	4	8.4813773E+01	5	5.4835777E+01	5	9.3282844E+00	5
	RECAA	5.9549521E+02	7	7.4261138E+02	7	4.7157363E+02	7	4.4878540E+01	7
	FFA	1.7600859E+02	6	2.1214779E+02	6	1.6668139E+02	6	1.1327390E+01	6
	KOA	1.3517630E+00	2	1.7142478E+00	2	1.2294459E+00	2	1.0224744E‐01	2
	RIME	6.4938021E+01	5	7.7960863E+01	4	5.3532145E+01	4	6.8826033E+00	4
SDM‐M6	WLS	7.0792061E+01	5	8.4328296E+01	4	5.8986579E+01	5	5.2937514E+00	3
	SWO	1.3983197E+00	1	1.4730137E+00	1	1.3280953E+00	2	3.9444251E‐02	1
	SGA	5.3684332E+01	3	8.8758009E+01	5	4.1367386E+01	4	1.5263117E+01	5
	RECAA	7.5499918E+02	7	8.3960997E+02	7	5.5540957E+02	7	7.6320880E+01	7
	FFA	2.1359657E+02	6	2.5401685E+02	6	1.7196503E+02	6	2.0900177E+01	6
	KOA	1.5978616E+00	2	1.9945658E+00	2	1.2894022E+00	1	1.6754344E‐01	2
	RIME	6.3316712E+01	4	7.9352806E+01	3	3.4234186E+01	3	7.9723885E+00	4
DDM‐M1	WLS	4.9993625E+01	4	6.1750302E+01	3	4.3864635E+01	5	4.4215335E+00	3
	SWO	9.6443799E‐01	1	1.3665931E+00	1	7.1190080E‐01	1	1.8866128E‐01	1
	SGA	4.7320575E+01	3	7.2229410E+01	5	3.8218199E+01	4	1.0443781E+01	5
	RECAA	7.2746940E+02	7	8.0159756E+02	7	6.5370464E+02	7	3.8357418E+01	6
	FFA	1.3481086E+02	6	2.2437298E+02	6	8.4101006E+01	6	4.6139289E+01	7
	KOA	1.4970136E+00	2	2.0646804E+00	2	1.1304145E+00	2	2.2594770E‐01	2
	RIME	5.3006300E+01	5	6.4625329E+01	4	3.7119285E+01	3	4.9813134E+00	4
DDM‐M2	WLS	5.9246258E+01	3	7.7035210E+01	3	4.0612611E+01	3	7.5411423E+00	4
	SWO	1.4389469E+00	1	2.0238092E+00	2	9.8648330E‐01	1	2.4347421E‐01	2
	SGA	7.1170018E+01	5	1.1292076E+02	5	4.9404908E+01	4	1.0900527E+01	5
	RECAA	7.4903216E+02	7	8.4215917E+02	7	5.1141271E+02	7	8.5229565E+01	7
	FFA	2.2414824E+02	6	2.6060914E+02	6	1.7497546E+02	6	2.0132025E+01	6
	KOA	1.5977548E+00	2	1.9981154E+00	1	1.3717026E+00	2	1.8428726E‐01	1
	RIME	6.6787451E+01	4	8.2404585E+01	4	5.5964063E+01	5	6.0624444E+00	3
DDM‐M3	WLS	5.6121144E+01	3	7.7812213E+01	4	4.1456433E+01	3	7.0590754E+00	4
	SWO	1.2268643E+00	1	1.4825475E+00	1	1.0268683E+00	1	1.2974580E‐01	1
	SGA	7.4732245E+01	5	1.1111650E+02	5	6.3373461E+01	5	8.5251000E+00	5
	RECAA	7.3408031E+02	7	8.0646256E+02	7	6.5782753E+02	7	5.1176857E+01	7
	FFA	1.9767851E+02	6	2.3475731E+02	6	1.6918988E+02	6	1.7278286E+01	6
	KOA	1.5312045E+00	2	2.0915155E+00	2	1.3026141E+00	2	1.8196259E‐01	2
	RIME	5.7959507E+01	4	7.0830247E+01	3	5.1077521E+01	4	4.3857925E+00	3
DDM‐M4	WLS	5.3452667E+01	3	6.7947184E+01	3	4.6003019E+01	3	5.6577017E+00	4
	SWO	1.3191802E+00	1	1.7795579E+00	1	1.0721450E+00	1	1.6299588E‐01	1
	SGA	7.0495055E+01	5	9.7149993E+01	5	5.9473258E+01	5	7.0141568E+00	5
	RECAA	7.3188754E+02	7	7.8741202E+02	7	6.6497058E+02	7	4.6451286E+01	7
	FFA	1.9522191E+02	6	2.3229296E+02	6	1.5485958E+02	6	1.6558447E+01	6
	KOA	1.4232051E+00	2	1.7827367E+00	2	1.1107483E+00	2	1.9963605E‐01	2
	RIME	5.7814463E+01	4	6.8588016E+01	4	5.0263380E+01	4	4.7257008E+00	3
DDM‐M5	WLS	5.9641067E+01	3	8.5675392E+01	4	4.0562162E+01	3	9.1131687E+00	5
	SWO	1.4612077E+00	1	1.8990891E+00	1	9.7515800E‐01	1	2.4424489E‐01	2
	SGA	7.8690249E+01	5	1.0638189E+02	5	6.2260244E+01	5	8.7350830E+00	4
	RECAA	7.4821967E+02	7	8.4057142E+02	7	5.0105636E+02	7	8.9165632E+01	7
	FFA	2.2954539E+02	6	2.6391740E+02	6	1.7764301E+02	6	2.0865152E+01	6
	KOA	1.6403162E+00	2	1.9836064E+00	2	1.4335783E+00	2	1.5088091E‐01	1
	RIME	6.9249354E+01	4	8.2471715E+01	3	5.9073250E+01	4	5.6144190E+00	3
DDM‐M6	WLS	5.3711251E+01	3	7.9599934E+01	3	4.1448554E+01	3	1.2700065E+01	4
	SWO	1.9430861E+00	2	2.7769586E+00	2	1.4653674E+00	1	3.3767416E‐01	2
	SGA	8.8667435E+01	4	1.0927146E+02	4	7.6241240E+01	5	8.9243286E+00	3
	RECAA	6.8936335E+02	7	8.5804832E+02	7	4.3551552E+02	7	1.6441701E+02	7
	FFA	2.6561664E+02	6	2.8658369E+02	6	1.9147278E+02	6	2.0167625E+01	6
	KOA	1.7554516E+00	1	2.1774359E+00	1	1.4886747E+00	2	1.6325186E‐01	1
	RIME	8.9199138E+01	5	1.2763841E+02	5	7.4192667E+01	4	1.5663348E+01	5
TDM‐M1	WLS	5.5046156E+01	4	7.7040542E+01	4	5.2702379E+01	4	5.1888434E+00	4
	SWO	1.0095272E+00	1	1.3994708E+00	1	7.6569520E‐01	1	1.8603776E‐01	1
	SGA	6.1844086E+01	5	9.2680376E+01	5	5.9319534E+01	5	6.3643887E+00	5
	RECAA	6.8515710E+02	7	7.9934605E+02	7	6.7066534E+02	7	2.2387458E+01	7
	FFA	1.7468337E+02	6	2.2466094E+02	6	1.6117401E+02	6	1.6801684E+01	6
	KOA	1.1357939E+00	2	1.5034896E+00	2	8.5045620E‐01	2	2.0619782E‐01	2
	RIME	5.4413203E+01	3	6.3240281E+01	3	4.5782737E+01	3	4.1549525E+00	3
TDM‐M2	WLS	6.5760908E+01	4	9.3549290E+01	4	4.7579000E+01	4	8.9279443E+00	4
	SWO	1.4079677E+00	1	1.8059622E+00	1	1.0983086E+00	1	1.8533270E‐01	1
	SGA	7.2550707E+01	5	1.1201051E+02	5	5.2076588E+01	5	1.0360669E+01	5
	RECAA	7.3325326E+02	7	8.6400752E+02	7	5.2869286E+02	7	5.3217929E+01	7
	FFA	2.0656586E+02	6	2.8087595E+02	6	1.5048338E+02	6	2.8659330E+01	6
	KOA	1.5542443E+00	2	1.9121637E+00	2	1.1509049E+00	2	2.3852113E‐01	2
	RIME	6.5176497E+01	3	7.8843488E+01	3	4.6755655E+01	3	7.7920290E+00	3
TDM‐M3	WLS	6.0062125E+01	4	8.4752308E+01	4	5.2347215E+01	4	6.2592862E+00	4
	SWO	1.2859645E+00	1	1.5481752E+00	1	9.7522150E‐01	1	1.5548762E‐01	1
	SGA	6.6932541E+01	5	9.8646795E+01	5	5.5400336E+01	5	7.0240353E+00	5
	RECAA	7.0315673E+02	7	8.2759914E+02	7	6.4409843E+02	7	2.6448500E+01	7
	FFA	1.8918335E+02	6	2.5042922E+02	6	1.5791859E+02	6	2.0015319E+01	6
	KOA	1.4218495E+00	2	1.6848482E+00	2	1.0409639E+00	2	1.8290060E‐01	2
	RIME	5.9767457E+01	3	6.9129392E+01	3	4.9738782E+01	3	4.6598088E+00	3
TDM‐M4	WLS	5.8138118E+01	4	8.1107777E+01	4	4.9647590E+01	4	6.1920043E+00	4
	SWO	1.1914925E+00	1	1.6600825E+00	2	9.8592890E‐01	1	2.0854273E‐01	2
	SGA	6.5253389E+01	5	9.5233624E+01	5	5.6155480E+01	5	6.5159258E+00	5
	RECAA	6.9675188E+02	7	8.1200173E+02	7	6.6348840E+02	7	2.3668178E+01	7
	FFA	1.8527515E+02	6	2.4892002E+02	6	1.5916407E+02	6	1.8691924E+01	6
	KOA	1.3328581E+00	2	1.5222992E+00	1	1.1818823E+00	2	7.7401058E‐02	1
	RIME	5.8088719E+01	3	6.7999355E+01	3	4.9586249E+01	3	4.2788174E+00	3
TDM‐M5	WLS	6.6184765E+01	4	9.5762377E+01	4	4.7719337E+01	4	9.6347289E+00	4
	SWO	1.3914892E+00	1	1.8421007E+00	1	8.5862590E‐01	1	2.3751860E‐01	1
	SGA	7.2587969E+01	5	1.0199551E+02	5	4.9819563E+01	5	1.0226260E+01	5
	RECAA	7.3338760E+02	7	8.6605097E+02	7	5.3043296E+02	7	5.3988279E+01	7
	FFA	2.0718460E+02	6	2.7895102E+02	6	1.4878028E+02	6	2.9880281E+01	6
	KOA	1.5832986E+00	2	1.9538636E+00	2	9.8937460E‐01	2	2.5086812E‐01	2
	RIME	6.5620716E+01	3	7.9411187E+01	3	4.6768761E+01	3	8.6962494E+00	3
TDM‐M6	WLS	7.2240433E+01	4	1.1189946E+02	5	4.5058179E+01	4	1.4560438E+01	5
	SWO	1.3496385E+00	1	1.9549905E+00	1	9.3276610E‐01	1	3.1155586E‐01	2
	SGA	7.5049555E+01	5	8.7554378E+01	3	4.9109632E+01	5	1.3306716E+01	3
	RECAA	7.6205777E+02	7	9.1608864E+02	7	4.7229015E+02	7	9.2534447E+01	7
	FFA	2.2601041E+02	6	3.1365666E+02	6	1.3742375E+02	6	4.5407722E+01	6
	KOA	1.5830870E+00	2	1.9640978E+00	2	1.0479733E+00	2	2.8792782E‐01	1
	RIME	7.1618169E+01	3	9.3289751E+01	4	4.2227715E+01	3	1.4096102E+01	4

PV model	Algorithm	Mean rank	Total rank	Mean rank	Total rank	Mean rank	Total rank	Mean rank	Total rank
SDM, DDM, and TDM	WLS	3.5000	3	3.5833	4	3.6667	3	3.7500	4
	**SWO**	**1.1250**	**1**	**1.2500**	**1**	**1.1250**	**1**	**1.3750**	**1**
	SGA	4.5833	5	4.8750	5	4.6250	5	4.7083	5
	RECAA	7.0000	7	7.0000	7	7.0000	7	6.9583	7
	FFA	6.0000	6	6.0000	6	6.0000	6	6.0417	6
	KOA	1.8750	2	1.7500	2	1.8750	2	1.6250	2
	RIME	3.9167	4	3.5417	3	3.7083	4	3.5417	3

### 4.7 Statistical Tests‐Based Assessment

After evaluating the results of algorithms for PV parameter extraction in 10000 iterations and 30 runs with evaluation metrics, we now evaluate them statistically. This statistical evaluation reveals the order of success of the algorithms in solving this problem and then allows a pairwise comparison between the most successful and the other algorithms. Statistical tests are presented in the following two subsections: the Friedman test and the Wilcoxon signed‐rank test.

#### 4.7.1 Friedman Test

Friedman test is a test used in a group of two or more algorithms to determine whether there is a significant difference between the algorithms and to emphasize the statistical significance of an algorithm. This nonparametric test is used to determine the performance of algorithms. It investigates whether there is a significant difference between algorithms, and then it compares and ranks them.^[^ ^65^, ^66^ ^]^ Within this concept, our WLS, SWO, SGA, RECAA, FFA, KOA, and RIME algorithms have been run for 10000 iterations, and this was repeated 30 times. **Table** **15** shows the results of Friedman test for 30 runs for the PV models. It includes the algorithm, the mean rank, the rank where the mean rank is ordered from the smallest to the largest, the *p*‐value, and the conclusion information. The p‐value is investigated for a significance level of 0.05 (5%). In this case, a *p*‐value less than 0.05 means that there is a significant difference between that group of algorithms. Based on this information, the p‐value comes up as less than 0.05 in all 24 PV models, suggesting that there is a significant difference between these seven algorithms in 24 groups. Next, the calculated mean rank is ordered from the smallest to the largest. Therefore, the algorithm with the smallest mean rank is considered the most successful algorithm in that group. The ranking of algorithms according to the mean rank in the Friedman test for 30 runs is given in **Table** **16**. In this table, the algorithm with the smallest mean rank value among the algorithms in 24 groups (24 PV models) is given a value of 1, and the algorithm with the largest value is given a value of 7. At the bottom of the table, the algorithms are averaged for the 24 models. According to this information, the WLS algorithm performed the best among the seven algorithms. The WLS is followed by RECAA, FFA, SGA, SWO, RIME, and KOA. In conclusion, the statistical significance of the WLS algorithm among the seven algorithms is emphasized through Friedman test results.

**Table 15:** Friedman test results for 30 runs.

Model	Alg.	Mean rank	Rank	*p*‐value	Conclusion (Is there a significant difference between the performances of the seven algorithms at 5% significance level?)	Model	Alg.	Mean rank	Rank	*p*‐value	Conclusion (Is there a significant difference between the performances of the seven algorithms at 5% significance level?)
SDM‐C1	WLS	1.0000	1	3.3295E‐27	*p*‐value < 0.05 3.3295E‐27 < 0.05 Yes.	SDM‐C2	WLS	1.0667	1	9.2220e‐17	*p*‐value < 0.05 9.2220e‐17< 0.05 Yes.
	SWO	3.3333	3				SWO	4.4000	4		
	SGA	3.8333	4				SGA	4.2000	3		
	RECAA	4.6667	5				RECAA	3.0667	2		
	FFA	2.7333	2				FFA	5.5333	7		
	KOA	6.0667	6				KOA	4.6333	5		
	RIME	6.3667	7				RIME	5.1000	6		
DDM‐C1	WLS	1.0333	1	1.8332e‐27	*p*‐value < 0.05 1.8332e‐27 < 0.05 Yes.	DDM‐C2	WLS	1.1000	1	6.4171e‐18	*p*‐value < 0.05 6.4171e‐18 < 0.05 Yes.
	SWO	4.2000	4				SWO	4.8000	5		
	SGA	2.9000	2				SGA	3.3333	3		
	RECAA	5.1667	6				RECAA	3.2667	2		
	FFA	2.9000	2				FFA	5.3333	6		
	KOA	6.8333	7				KOA	5.5000	7		
	RIME	4.9667	5				RIME	4.6667	4		
TDM‐C1	WLS	1.0667	1	3.9528e‐28	*p*‐value < 0.05 3.9528e‐28< 0.05 Yes.	TDM‐C2	WLS	1.4000	1	6.6013e‐19	*p*‐value < 0.05 6.6013e‐19 < 0.05 Yes.
	SWO	5.0333	6				SWO	4.5333	5		
	SGA	2.2000	2				SGA	3.2000	3		
	RECAA	4.4667	5				RECAA	3.1333	2		
	FFA	3.9000	3				FFA	5.6333	6		
	KOA	6.9667	7				KOA	6.0333	7		
	RIME	4.3667	4				RIME	4.0667	4		
SDM‐M1	WLS	1.0000	1	7.8034e‐35	*p*‐value < 0.05 7.8034e‐35< 0.05) Yes.	SDM‐M2	WLS	1.0000	1	2.0063e‐27	*p*‐value < 0.05 (2.0063e‐27 < 0.05 Yes.
	SWO	4.8667	5				SWO	4.8667	6		
	SGA	4.0000	4				SGA	4.7333	5		
	RECAA	3.0667	3				RECAA	3.2333	3		
	FFA	2.0667	2				FFA	2.6333	2		
	KOA	6.2333	6				KOA	6.9000	7		
	RIME	6.7667	7				RIME	4.6333	4		
SDM‐M3	WLS	1.0000	1	7.1638e‐31	*p*‐value < 0.05 7.1638e‐31< 0.05 Yes.	SDM‐M4	WLS	1.0000	1	3.1292e‐34	*p*‐value < 0.05 3.1292e‐34 < 0.05 Yes.
	SWO	4.3667	4				SWO	4.6000	5		
	SGA	4.3667	4				SGA	4.3000	4		
	RECAA	2.5667	2				RECAA	2.9667	3		
	FFA	2.9667	3				FFA	2.1667	2		
	KOA	6.8667	7				KOA	6.9000	7		
	RIME	5.8667	6				RIME	6.0667	6		
SDM‐M5	WLS	1.0667	1	2.9161e‐33	*p*‐value < 0.05 2.9161e‐33 < 0.05 Yes.	SDM‐M6	WLS	1.8833	2	9.8316e‐26	*p*‐value < 0.05 9.8316e‐26 < 0.05 Yes.
	SWO	5.1000	5				SWO	5.0000	5		
	SGA	3.9000	4				SGA	4.7000	4		
	RECAA	2.4333	2				RECAA	3.9000	3		
	FFA	2.7333	3				FFA	1.1167	1		
	KOA	6.9333	7				KOA	6.0333	7		
	RIME	5.8333	6				RIME	5.3667	6		
DDM‐M1	WLS	1.0000	1	2.4959e‐32	*p*‐value<0.05 2.4959e‐32< 0.05 Yes.	DDM‐M2	WLS	1.1333	1	8.7550e‐29	*p*‐value < 0.05 8.7550e‐29 < 0.05 Yes.
	SWO	5.4000	5				SWO	5.2000	6		
	SGA	3.5667	4				SGA	4.3667	4		
	RECAA	2.7333	2				RECAA	3.4000	3		
	FFA	2.8333	3				FFA	2.2667	2		
	KOA	6.9667	7				KOA	7.0000	7		
	RIME	5.5000	6				RIME	4.6333	5		
DDM‐M3	WLS	1.0000	1	7.7164e‐32	*p*‐value < 0.05 7.7164e‐32< 0.05 Yes.	DDM‐M4	WLS	1.0000	1	7.8246e‐32	*p*‐value < 0.05 7.8246e‐32< 0.05 Yes.
	SWO	4.9000	5				SWO	4.7333	5		
	SGA	3.9333	4				SGA	4.3667	4		
	RECAA	2.3667	2				RECAA	2.7333	3		
	FFA	3.1000	3				FFA	2.5667	2		
	KOA	6.8333	7				KOA	6.9667	7		
	RIME	5.8667	6				RIME	5.6333	6		
DDM‐M5	WLS	1.0667	1	3.4474e‐33	*p*‐value < 0.05 3.4474e‐33 < 0.05 Yes.	DDM‐M6	WLS	1.8333	2	2.1752e‐28	*p*‐value < 0.05 2.1752e‐28< 0.05 Yes.
	SWO	4.9667	5				SWO	5.1667	6		
	SGA	3.3000	4				SGA	4.8000	5		
	RECAA	2.6000	2				RECAA	3.5333	3		
	FFA	3.0667	3				FFA	1.2333	1		
	KOA	6.9667	7				KOA	6.6667	7		
	RIME	6.0333	6				RIME	4.7667	4		
TDM‐M1	WLS	1.0333	1	1.4170e‐30	*p*‐value<0.05 1.4170e‐30< 0.05 Yes.	TDM‐M2	WLS	1.2667	1	9.4243e‐27	p‐value<0.05 9.4243e‐27< 0.05) Yes.
	SWO	5.7000	6				SWO	5.4333	6		
	SGA	3.2333	3				SGA	3.5333	3		
	RECAA	2.5333	2				RECAA	3.5333	3		
	FFA	3.7000	4				FFA	2.6667	2		
	KOA	6.9333	7				KOA	7.0000	7		
	RIME	4.8667	5				RIME	4.5667	5		
TDM‐M3	WLS	1.0000	1	1.2920e‐31	*p*‐value<0.05 1.2920e‐31< 0.05 Yes.	TDM‐M4	WLS	1.0667	1	4.7508e‐31	*p*‐value<0.05 4.7508e‐31< 0.05 Yes.
	SWO	5.1000	5				SWO	5.1333	5		
	SGA	3.7000	4				SGA	4.1333	4		
	RECAA	2.1667	2				RECAA	2.4000	2		
	FFA	3.6667	3				FFA	2.9667	3		
	KOA	7.0000	7				KOA	7.0000	7		
	RIME	5.3667	6				RIME	5.3000	6		
TDM‐M5	WLS	1.7000	1	8.9511e‐31	*p*‐value<0.05 8.9511e‐31< 0.05) Yes.	TDM‐M6	WLS	1.8333	2	2.1805e‐29	*p*‐value<0.05 2.1805e‐29< 0.05 Yes.
	SWO	5.1000	5				SWO	5.7000	6		
	SGA	3.0000	3				SGA	4.5333	5		
	RECAA	2.2000	2				RECAA	3.6333	3		
	FFA	3.1000	4				FFA	1.3000	1		
	KOA	6.9333	7				KOA	6.7667	7		
	RIME	5.9667	6				RIME	4.2333	4		

**Table 16:** Ranking of algorithms according to the mean rank in the Friedman test for 30 runs.

PV model/algorithm	WLS	SWO	SGA	RECAA	FFA	KOA	RIME
SDM‐C1	1	3	4	5	2	6	7
SDM‐C2	1	4	3	2	7	5	6
DDM‐C1	1	4	2	6	2	7	5
DDM‐C2	1	5	3	2	6	7	4
TDM‐C1	1	6	2	5	3	7	4
TDM‐C2	1	5	3	2	6	7	4
SDM‐M1	1	5	4	3	2	6	7
SDM‐M2	1	6	5	3	2	7	4
SDM‐M3	1	4	4	2	3	7	6
SDM‐M4	1	5	4	3	2	7	6
SDM‐M5	1	5	4	2	3	7	6
SDM‐M6	2	5	4	3	1	7	6
DDM‐M1	1	5	4	2	3	7	6
DDM‐M2	1	6	4	3	2	7	5
DDM‐M3	1	5	4	2	3	7	6
DDM‐M4	1	5	4	3	2	7	6
DDM‐M5	1	5	4	2	3	7	6
DDM‐M6	2	6	5	3	1	7	4
TDM‐M1	1	6	3	2	4	7	5
TDM‐M2	1	6	3	3	2	7	5
TDM‐M3	1	5	4	2	3	7	6
TDM‐M4	1	5	4	2	3	7	6
TDM‐M5	1	5	3	2	4	7	6
TDM‐M6	2	6	5	3	1	7	4
Mean rank	1.1250	5.0833	3.7083	2.7917	2.9167	6.8333	5.4167
Total rank	**1**	5	4	2	3	7	6

#### 4.7.2 Wilcoxon Signed‐Rank Test

The second statistical test in addition to the Friedman test, in which the order of performance of algorithms in a group is obtained, is the Wilcoxon signed‐rank test. In this nonparametric test, algorithms are compared in pairs. As a result of this comparison, it is determined whether one algorithm is superior to the other algorithm.^[^ ^14^ ^]^ According to the Friedman test results shown in Tables 15 and 16, the statistical superiority of the WLS algorithm among the seven algorithms is emphasized and statistically proven to be the most successful algorithm. This information, which is obtained from the Friedman test that the WLS algorithm ranked first among the seven algorithms, is used in the Wilcoxon signed‐rank test. Wilcoxon signed‐rank test is used to compare the WLS with SWO, SGA, RECAA, FFA, KOA, and RIME algorithms. Results of Wilcoxon signed‐rank test for 30 runs are given in **Table** **17**. 24 PV models and 6 algorithms were compared pairwise in each PV model. In total, 144 algorithms were compared pairwise. The p‐value for the significance level of 0.05 (5%) is investigated here, too. A *p*‐value that is less than 0.05 means that there is a significant difference between WLS and the other compared algorithms. In all 144 comparisons, the p‐value is less than 0.05, suggesting that the WLS is superior to the other six algorithms in all 24 models. Next, the calculated p‐value for each group of PV models is ranked from the smallest to the largest. The algorithm with the smallest p‐value is considered the most successful algorithm in that comparison pair. The ranking of algorithms according to the p‐value in the Wilcoxon signed‐rank test for 30 runs is given in **Table** **18**. In this table, the algorithm with the smallest p‐value among the algorithms in the 24 PV models has a value of 1, and the algorithm with the largest *p*‐value has a value of 6. At the end of the table, the average of the algorithms for the 24 PV models is taken. According to the information obtained from 144 algorithm comparisons, the WLS algorithm performs the best, followed by SWO and KOA, RIME, RECAA, FFA, and SGA. Here, SWO and KOA are the closest algorithms to the performance of the WLS algorithm, while SGA produced the farthest result. According to the Wilcoxon signed‐rank results, the WLS outperforms the competing algorithm in 144 out of 144 pairwise algorithm comparisons, emphasizing the statistical superiority of the WLS over other algorithms.

**Table 17:** Wilcoxon signed‐rank test results for 30 runs.

Model	WLS vs compared Alg.	p‐value	H	Zval	Ranksum	IS/IS NOT	Rank	Model	WLS vs compared Alg.	p‐value	H	Zval	Ranksum	IS/IS NOT	Rank
SDM‐C1	SWO	3.0179668E‐11	1	−6.6457	465	IS	1	SDM‐C2	SWO	3.0198594E‐11	1	−6.6456	465	IS	1
	SGA	3.0179668E‐11	1	−6.6457	465	IS	1		SGA	6.0877555E‐10	1	−6.1881	496	IS	4
	RECAA	3.0179668E‐11	1	−6.6457	465	IS	1		RECAA	3.0198594E‐11	1	−6.6456	465	IS	1
	FFA	3.0179668E‐11	1	−6.6457	465	IS	1		FFA	2.8250456E‐10	1	−6.3081	500	IS	3
	KOA	3.0179668E‐11	1	−6.6457	465	IS	1		KOA	3.0198594E‐11	1	−6.6456	465	IS	1
	RIME	3.0179668E‐11	1	−6.6457	465	IS	1		RIME	4.0771648E‐11	1	−6.6012	468	IS	2
DDM‐C1	SWO	4.5043221E‐11	1	−6.5865	469	IS	2	DDM‐C2	SWO	3.0198594E‐11	1	−6.6456	465	IS	1
	SGA	3.4741966E‐10	1	−6.2760	490	IS	3		SGA	7.3802859E‐10	1	−6.1577	498	IS	5
	RECAA	3.0198594E‐11	1	−6.6456	465	IS	1		RECAA	4.0771648E‐11	1	−6.6012	468	IS	3
	FFA	3.0198594E‐11	1	−6.6456	465	IS	1		FFA	2.5168393E‐08	1	−5.5721	547	IS	6
	KOA	3.0198594E‐11	1	−6.6456	465	IS	1		KOA	3.6897259E‐11	1	−6.6160	467	IS	2
	RIME	3.0198594E‐11	1	−6.6456	465	IS	1		RIME	2.8715848E‐10	1	−6.3056	488	IS	4
TDM‐C1	SWO	4.9751664E‐11	1	−6.5717	470	IS	3	TDM‐C2	SWO	3.0198594E‐11	1	−6.6456	465	IS	1
	SGA	4.6856320E‐08	1	−5.4628	545	IS	5		SGA	5.0911735E‐06	1	−4.5610	606	IS	6
	RECAA	5.4940525E‐11	1	−6.5569	471	IS	4		RECAA	4.9751664E‐11	1	−6.5717	470	IS	2
	FFA	3.0198594E‐11	1	−6.6456	465	IS	1		FFA	3.4277131E‐08	1	−5.5181	555	IS	4
	KOA	3.0198594E‐11	1	−6.6456	465	IS	1		KOA	1.3288512E‐10	1	−6.4238	480	IS	3
	RIME	4.0771648E‐11	1	−6.6012	468	IS	2		RIME	9.8328906E‐08	1	−5.3298	554	IS	5
SDM‐M1	SWO	3.0141849E‐11	1	−6.6459	465	IS	1	SDM‐M2	SWO	3.0198594E‐11	1	−6.6456	465	IS	1
	SGA	3.0141849E‐11	1	−6.6459	465	IS	1		SGA	3.0198594E‐11	1	−6.6456	465	IS	1
	RECAA	3.0141849E‐11	1	−6.6459	465	IS	1		RECAA	3.0198594E‐11	1	−6.6456	465	IS	1
	FFA	3.0141849E‐11	1	−6.6459	465	IS	1		FFA	5.4940525E‐11	1	−6.5569	471	IS	2
	KOA	3.0141849E‐11	1	−6.6459	465	IS	1		KOA	3.0198594E‐11	1	−6.6456	465	IS	1
	RIME	3.0141849E‐11	1	−6.6459	465	IS	1		RIME	3.0198594E‐11	1	−6.6456	465	IS	1
SDM‐M3	SWO	3.0104074E‐11	1	−6.6461	465	IS	1	SDM‐M4	SWO	3.0085202E‐11	1	−6.6462	465	IS	1
	SGA	3.0104074E‐11	1	−6.6461	465	IS	1		SGA	3.0085202E‐11	1	−6.6462	465	IS	1
	RECAA	3.0104074E‐11	1	−6.6461	465	IS	1		RECAA	3.0085202E‐11	1	−6.6462	465	IS	1
	FFA	3.0104074E‐11	1	−6.6461	465	IS	1		FFA	3.0085202E‐11	1	−6.6462	465	IS	1
	KOA	3.0104074E‐11	1	−6.6461	465	IS	1		KOA	3.0085202E‐11	1	−6.6462	465	IS	1
	RIME	3.0104074E‐11	1	−6.6461	465	IS	1		RIME	3.0085202E‐11	1	−6.6462	465	IS	1
SDM‐M5	SWO	3.0198594E‐11	1	−6.6456	465	IS	1	SDM‐M6	SWO	2.9766032E‐11	1	−6.6477	465	IS	1
	SGA	3.0198594E‐11	1	−6.6456	465	IS	1		SGA	2.9766032E‐11	1	−6.6477	465	IS	1
	RECAA	8.1527445E‐11	1	−6.4978	475	IS	2		RECAA	2.9766032E‐11	1	−6.6477	465	IS	1
	FFA	6.1210394E‐10	1	−6.1873	496	IS	4		FFA	2.9282599E‐07	1	5.1280	1262	IS	2
	KOA	3.0198594E‐11	1	−6.6456	465	IS	1		KOA	2.9766032E‐11	1	−6.6477	465	IS	1
	RIME	5.5726532E‐10	1	−6.2021	495	IS	3		RIME	2.9766032E‐11	1	−6.6477	465	IS	1
DDM‐M1	SWO	3.0198594E‐11	1	−6.6456	465	IS	1	DDM‐M2	SWO	3.0198594E‐11	1	−6.6456	465	IS	1
	SGA	3.0198594E‐11	1	−6.6456	465	IS	1		SGA	3.1967402E‐09	1	−5.9212	514	IS	3
	RECAA	3.0198594E‐11	1	−6.6456	465	IS	1		RECAA	4.5043221E‐11	1	−6.5865	469	IS	2
	FFA	3.0198594E‐11	1	−6.6456	465	IS	1		FFA	3.0102616E‐07	1	−5.1228	568	IS	4
	KOA	3.0198594E‐11	1	−6.6456	465	IS	1		KOA	3.0198594E‐11	1	−6.6456	465	IS	1
	RIME	3.0198594E‐11	1	−6.6456	465	IS	1		RIME	3.0198594E‐11	1	−6.6456	465	IS	1
DDM‐M3	SWO	3.0179668E‐11	1	−6.6457	465	IS	1	DDM‐M4	SWO	3.3321431E‐11	1	−6.6311	466	IS	2
	SGA	3.0179668E‐11	1	−6.6457	465	IS	1		SGA	3.0141849E‐11	1	−6.6459	465	IS	1
	RECAA	3.0179668E‐11	1	−6.6457	465	IS	1		RECAA	3.6828530E‐11	1	−6.6163	467	IS	3
	FFA	3.0179668E‐11	1	−6.6457	465	IS	1		FFA	4.0696034E‐11	1	−6.6015	468	IS	4
	KOA	3.0179668E‐11	1	−6.6457	465	IS	1		KOA	3.0141849E‐11	1	−6.6459	465	IS	1
	RIME	3.0179668E‐11	1	−6.6457	465	IS	1		RIME	3.0141849E‐11	1	−6.6459	465	IS	1
DDM‐M5	SWO	3.0198594E‐11	1	−6.6456	465	IS	1	DDM‐M6	SWO	3.0028652E‐11	1	−6.6464	465	IS	1
	SGA	3.0810544E‐08	1	−5.5368	540	IS	4		SGA	5.5452476E‐10	1	−6.2028	495	IS	2
	RECAA	1.5465213E‐09	1	−6.0394	506	IS	3		RECAA	3.0028652E‐11	1	−6.6464	465	IS	1
	FFA	1.2056679E‐10	1	−6.4386	479	IS	2		FFA	6.3381584E‐05	1	3.9999	1186	IS	3
	KOA	3.0198594E‐11	1	−6.6456	465	IS	1		KOA	3.0028652E‐11	1	−6.6464	465	IS	1
	RIME	3.0198594E‐11	1	−6.6456	465	IS	1		RIME	3.0028652E‐11	1	−6.6464	465	IS	1
DDM‐M1	SWO	4.5043221E‐11	1	−6.5865	469	IS	3	DDM‐M2	SWO	3.0198594E‐11	1	−6.6456	465	IS	1
	SGA	1.3288512E‐10	1	−6.4238	480	IS	5		SGA	4.1127057E‐07	1	−5.0637	572	IS	3
	RECAA	1.4643069E‐10	1	−6.4090	481	IS	6		RECAA	1.4643069E‐10	1	−6.4090	481	IS	2
	FFA	5.4940525E‐11	1	−6.5569	471	IS	4		FFA	4.8010690E‐07	1	−5.0341	574	IS	4
	KOA	3.0198594E‐11	1	−6.6456	465	IS	1		KOA	3.0198594E‐11	1	−6.6456	465	IS	1
	RIME	3.3383888E‐11	1	−6.6308	466	IS	2		RIME	3.0198594E‐11	1	−6.6456	465	IS	1
DDM‐M3	SWO	3.0198594E‐11	1	−6.6456	465	IS	1	DDM‐M4	SWO	3.0179668E‐11	1	−6.6457	465	IS	1
	SGA	6.6955190E‐11	1	−6.5273	473	IS	3		SGA	3.0179668E‐11	1	−6.6457	465	IS	1
	RECAA	3.1588895E‐10	1	−6.2908	489	IS	4		RECAA	1.6122875E‐10	1	−6.3944	482	IS	3
	FFA	3.0198594E‐11	1	−6.6456	465	IS	1		FFA	6.6914678E‐11	1	−6.5274	473	IS	2
	KOA	3.0198594E‐11	1	−6.6456	465	IS	1		KOA	3.0179668E‐11	1	−6.6457	465	IS	1
	RIME	3.6897259E‐11	1	−6.6160	467	IS	2		RIME	3.0179668E‐11	1	−6.6457	465	IS	1
DDM‐M5	SWO	3.0198594E‐11	1	−6.6456	465	IS	1	DDM‐M6	SWO	3.0198594E‐11	1	−6.6456	465	IS	1
	SGA	7.6587874E‐05	1	−3.9548	647	IS	4		SGA	1.0104533E‐08	1	−5.7290	527	IS	3
	RECAA	1.3831620E‐02	1	−2.4616	748	IS	5		RECAA	3.0198594E‐11	1	−6.6456	465	IS	1
	FFA	1.0187689E‐05	1	−4.4132	616	IS	3		FFA	1.3249511E‐04	1	3.8218	1174	IS	4
	KOA	3.0198594E‐11	1	−6.6456	465	IS	1		KOA	3.0198594E‐11	1	−6.6456	465	IS	1
	RIME	3.6897259E‐11	1	−6.6160	467	IS	2		RIME	1.6132250E‐10	1	−6.3943	482	IS	2

**Table 18:** Ranking of algorithms according to the *p*‐value in the Wilcoxon signed‐rank test for 30 runs.

PV model/algorithm	SWO	SGA	RECAA	FFA	KOA	RIME
SDM‐C1	1	1	1	1	1	1
SDM‐C2	1	4	1	3	1	2
DDM‐C1	2	3	1	1	1	1
DDM‐C2	1	5	3	6	2	4
TDM‐C1	3	5	4	1	1	2
TDM‐C2	1	6	2	4	3	5
SDM‐M1	1	1	1	1	1	1
SDM‐M2	1	1	1	2	1	1
SDM‐M3	1	1	1	1	1	1
SDM‐M4	1	1	1	1	1	1
SDM‐M5	1	1	2	4	1	3
SDM‐M6	1	1	1	2	1	1
DDM‐M1	1	1	1	1	1	1
DDM‐M2	1	3	2	4	1	1
DDM‐M3	1	1	1	1	1	1
DDM‐M4	2	1	3	4	1	1
DDM‐M5	1	4	3	2	1	1
DDM‐M6	1	2	1	3	1	1
TDM‐M1	3	5	6	4	1	2
TDM‐M2	1	3	2	4	1	1
TDM‐M3	1	3	4	1	1	2
TDM‐M4	1	1	3	2	1	1
TDM‐M5	1	4	5	3	1	2
TDM‐M6	1	3	1	4	1	2
Mean rank	1.2500	2.5417	2.1250	2.5000	1.1250	1.6250
Total rank	1	5	3	4	1	2

### 4.8 Convergence Curves

The solution generation process of WLS, SWO, SGA, RECAA, FFA, KOA, and RIME algorithms for PV parameter extraction with 10000 iterations is given in **Figure** **11**. This figure shows the convergence curves for SDM, DDM, and TDM at the 30^th^ run.

Figure 11Convergence curves for SDM, DDM, and TDM at the 30^th^ runs.

**Figure:**
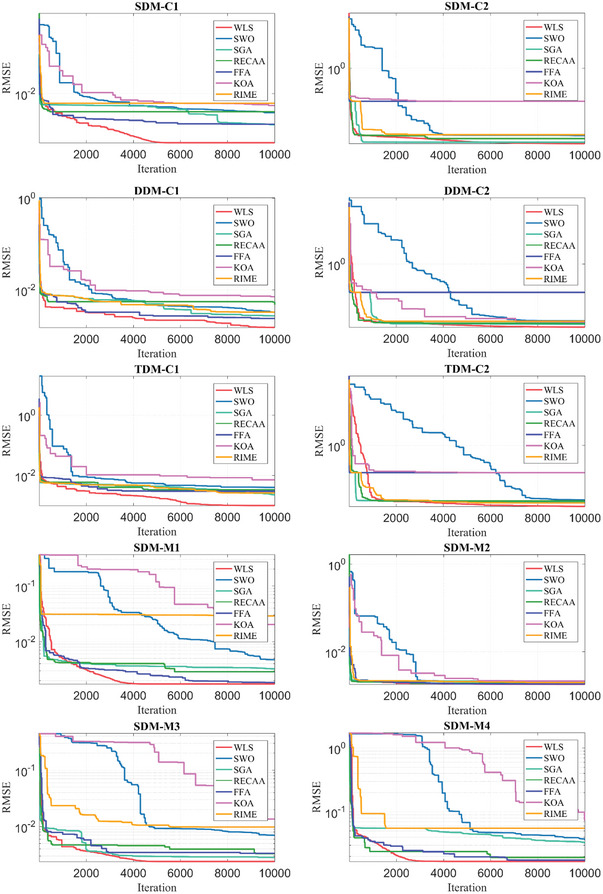

**Figure:**
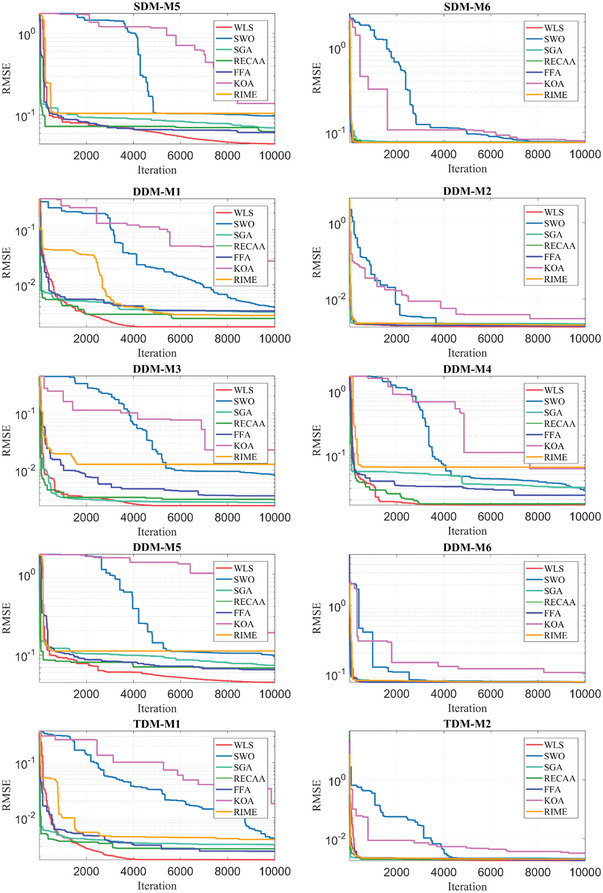

**Figure:**
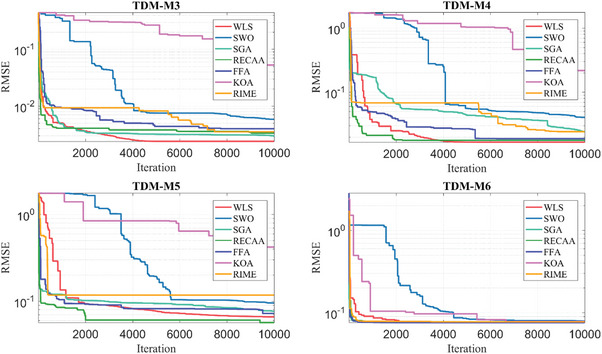

### 4.9 Sensitivity Analysis

**Figure 12:**
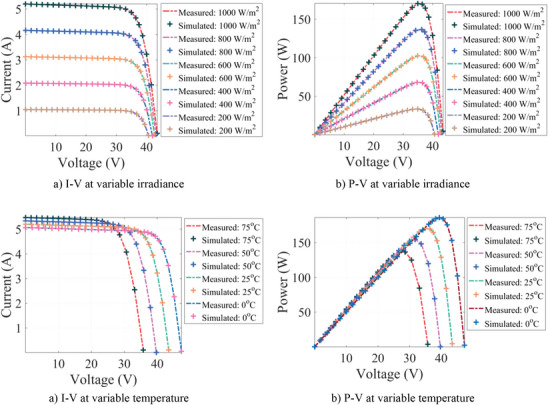
Experimental measurement data versus estimated *I*–*V* and *P*–*V* by WLS for SDM‐C under variable irradiance and temperature conditions.

Up to this point, we have demonstrated the superiority of the WLS algorithm in PV parameter extraction under constant irradiance and temperature conditions only. One can refer to Sections 4.5 and 4.6 for an overall comparison of the WLS algorithm with the other seven algorithms for the reliability aspect and Section 4.7 for statistical performance. It is also worth mentioning that parameter extraction has been made using the experimentally measured current and voltage data of PV cells consisting of R.T.C France and PVM 752, and the PV modules consisting of Schutten Solar STM6‐40/36, Leibold Solar Module (LSM 20), Photowatt‐PWP 201, Schutten Solar STP6‐120/36, Kyocera KC200GT 215, and ESP‐160 PPW under certain irradiance and temperature conditions. Now, in this section, we will analyze the accuracy of the parameter extraction performance of the WLS algorithm under varying irradiance and temperature conditions. This time, the performance tests were conducted using the experimentally measured current and voltage data under “constant irradiance‐variable temperature” and “variable irradiance‐constant temperature” conditions. The parameter extraction was made over the manufacturer values included in PV datasheets, as well as the measured current and voltage data. In brief, this analysis aims to investigate the stability of the WLS algorithm, its ability to adapt to complex conditions in real life, and its applicability to complex engineering problems. For this purpose, a polycrystalline BP 3170B module consisting of 72 series‐connected cells was selected, and the data provided by the manufacturer in the datasheet was used. This module has a maximum power of 170 W under standard test conditions of 1000 W/m^2^ and 25 °C. The voltage and the current at the maximum power point, open circuit voltage, and short circuit current are 35.6 V, 4.8 A, 43.6 V, and 5.2 A, respectively. Sensitivity analysis results at constant irradiance‐variable temperature and variable irradiance‐constant temperature conditions are given in **Table** **19**. The PV module was modeled as SDM, and the WLS algorithm was rerun 30 times with 10000 iterations. Table 19 shows the results of the parameter extraction and evaluation metric in two stages with the WLS. The first stage is the constant irradiance‐variable temperature case. In this case, the irradiance is constant at 1000 W/m^2^, and four temperature conditions are evaluated: 0 °C, 25 °C, 50 °C, and 75 °C. When the variation of the five estimated parameters with temperature is analyzed; increasing temperature has an increasing effect on *I*_ph_, *I*_o_, and *R*_s_ parameters, while it has a decreasing effect on *R*_sh_ and *α*. The second stage is the variable irradiance‐constant temperature case. In this case, the temperature was constant at 25 °C and five irradiance conditions were evaluated: 200, 400, 600, 800, and 1000 W/m^2^. When the effect of irradiation is analyzed, we can see that increasing irradiation has an increasing effect on the parameters *I*_ph_, *I*_o_, *R*_s_, and *α*, and a decreasing effect on *R*_sh_. The experimentally measured data versus the estimated *I*–*V* and *P*–*V* by WLS for SDM‐C under variable irradiance and temperature conditions are given in **Figure** **12**. When these graphs are examined, it is seen that the measured and estimated data overlap and verify the successful performance of the WLS algorithm once gain. In conclusion, the sensitivity analysis has reinforced the confidence in the WLS algorithm also under variable conditions.

**Table 19:** Sensitivity analysis results at variable irradiance and temperature conditions.

Irradiance [W/m^2^]	Temperature [°C]	*I*_ph_ [A]	*I*_o_ [µA]	*R*_sh_ [Ω]	*R*_s_ [Ω]	*α*	RMSE	RMSE average (30 runs)	RMSE standard deviation (30 runs)
1000	0	5.0430426	0.0066596	7.3937483	0.0045622	1.3690787	3.8598635E‐03	3.7092241E‐03	1.5819343E‐04
1000	25	5.1850775	0.0181747	6.2942171	0.0057069	1.2112308	2.6870223E‐03	2.6732030E‐03	1.7654898E‐04
1000	50	5.3333088	0.0846490	4.8879664	0.0065720	1.1052147	1.6012396E‐03	1.6717178E‐03	1.0722749E‐04
1000	75	5.4742434	0.6477630	4.4863553	0.0070532	1.0427069	8.7848930E‐03	5.1359275E‐03	1.2786890E‐04
200	25	1.0406985	0.0012838	20.4877621	0.0036143	1.0742218	2.2686649E‐03	2.2056358e‐03	2.8024889E‐04
400	25	2.0797437	0.0032295	11.1591129	0.0046249	1.1194972	6.3043185E‐03	6.7710989E‐03	7.9297141E‐04
600	25	3.1147598	0.0084995	9.0755246	0.0049175	1.1708325	1.3807313E‐03	1.4349394E‐03	1.0231514E‐04
800	25	4.1506405	0.0168098	7.2495808	0.0051948	1.2093873	2.0501766E‐03	1.9685734E‐03	1.1272278E‐04
1000	25	5.1863947	0.0253578	6.3581339	0.0055130	1.2322735	2.9436674E‐03	2.7806301E‐03	1.6456436E‐04

## 5 Conclusions

The way to accurately reflect the characteristics of PV systems requires the development of exact PV models. More specifically, it means that the model must work with the correct parameters. The PV parameter extraction is a challenging task due to its complexity and nonlinearity, which requires a great deal of effort by the researchers in this field. However, research is ongoing to achieve the most accurate PV model since improvements are still needed. Therefore, this article studies PV parameter extraction from a greater content. Except for KOA, the WLS, SWO, SGA, RECAA, FFA, and RIME algorithms were used for the first time in this paper to solve this optimization problem. For this purpose, PV cells and modules are modeled as SDM, DDM, and TDM. A wide range of different PV structures are used to test the developed models, including silicon, monocrystalline, monocrystalline, polycrystalline, multicrystalline, and thin. The experimental data (current and voltage) of two PV cells and six PV modules were used to test the performance of the algorithms in a total of 24 models. According to the evaluation metrics, among the seven algorithms, the lowest/successful minimum with 2.2572566E‐04, the smallest average with 2.3861506E‐04, and the smallest maximum RMSE with 2.4697146E‐04 were obtained with the WLS. With WLS, the best minimum, average, and maximum RMSE values from the closest algorithm to the farthest algorithm are 0.95% to 27.15%, 69.65% to 814.44%, and 95.25% to 814.93%, respectively. Moreover, WLS showed a very stable behavior in achieving these results with a standard deviation of 4.6095633E‐18. According to Friedman's test, it is proven that the WLS algorithm produces the most successful results among the seven algorithms. According to Wilcoxon signed‐rank test, the results of the pairwise comparison of WLS with the other six algorithms are more significant in all 144 comparisons. Then, WLS was tested for variable irradiance and temperature conditions, and sensitivity analysis was performed. As a result of the analysis performed under variable conditions, it was seen that the measured and estimated data successfully overlapped. As a result of the evaluation metrics, statistical tests, and sensitivity analysis, the WLS algorithm performed well in terms of accuracy and reliability, emphasizing its statistical importance and superiority. In extracting unknown PV parameters, the WLS algorithm is confirmed to be promising. It is concluded that it is a powerful and competitive alternative that can be used with confidently.

## Conflict of Interest

The authors declare no conflict of interest.

## Data Availability

Research data are not shared.
